# Synthesis, H_2_S releasing properties, antiviral and antioxidant activities and acute cardiac effects of nucleoside 5′-dithioacetates

**DOI:** 10.1038/s41598-025-85351-1

**Published:** 2025-01-22

**Authors:** Miklós Bege, Miklós Lovas, Dániel Priksz, Brigitta Bernát, Ilona Bereczki, Rasha Ghanem Kattoub, Richárd Kajtár, Simon Eskeif, Levente Novák, Jan Hodek, Jan Weber, Pál Herczegh, István Lekli, Anikó Borbás

**Affiliations:** 1 Department of Pharmaceutical Chemistry, Faculty of Pharmacy, University of Debrecen, Egyetem tér 1, Debrecen, 4032 Hungary; 2 HUN-REN-UD Molecular Recognition and Interaction Research Group, University of Debrecen, Egyetem tér 1, Debrecen, 4032 Hungary; 3 Institute of Healthcare Industry, University of Debrecen, Nagyerdei krt. 98, Debrecen, 4032 Hungary; 4 Department of Pharmacology and Pharmacotherapy, Faculty of Medicine, University of Debrecen, Nagyerdei krt. 98, Debrecen, 4032 Hungary; 5 HUN-REN-UD Pharmamodul Research Group, University of Debrecen, Nagyerdei krt. 98, Debrecen, 4032 Hungary; 6 National Laboratory of Virology, University of Pécs, Ifjúság útja 20, Pécs, 7624 Hungary; 7 Doctoral School of Pharmaceutical Sciences, Faculty of Pharmacy, University of Debrecen, Nagyerdei krt. 98, Debrecen, 4032 Hungary; 8 Department of Pharmacodynamics, Faculty of Pharmacy, University of Debrecen, Rex Ferenc u. 1., Debrecen, 4002 Hungary; 9 Department of Physical Chemistry, Faculty of Science and Technology, University of Debrecen, Egyetem tér 1, Debrecen, 4032 Hungary; 10 Institute of Organic Chemistry and Biochemistry, Czech Academy of Sciences, Flemingovo nam. 2, Prague, 166 10 Czech Republic

**Keywords:** Nucleoside, Dithioacetate, H_2_S donor, H_2_S release kinetics, Antioxidant, Antiviral, SARS-CoV-2, Medicinal chemistry, Pharmacology, Medicinal chemistry, Organic chemistry, Chemical synthesis, Cardiology, Chemistry

## Abstract

Hydrogen sulfide (H_2_S) is an endogenous gasotransmitter with cardioprotective and antiviral effects. In this work, new cysteine-selective nucleoside-H_2_S-donor hybrid molecules were prepared by conjugating nucleoside biomolecules with a thiol-activatable dithioacetyl group. 5′-Dithioacetate derivatives were synthesized from the canonical nucleosides (uridine, adenosine, cytidine, guanosine and thymidine), and the putative 5′-thio metabolites were also produced from uridine and adenosine. According to our measurements made with an H_2_S-specific sensor, nucleoside dithioacetates are moderately fast H_2_S donors, the guanosine derivative showed the fastest kinetics and the adenosine derivative the slowest. The antioxidant activity of 5′-thionucleosides is significantly higher than that of trolox, but lower than that of ascorbic acid, while intact dithioacetates have no remarkable antioxidant effect. In human Calu cells, the guanosine derivative showed a moderate anti-SARS-CoV-2 effect which was also confirmed by virus yield reduction assay. Dithioacetyl-adenosine and its metabolite showed similar acute cardiac effects as adenosine, however, it is noteworthy that both 5′-thio modified adenosines increased left ventricular ejection fraction or stroke volume, which was not observed with native adenosine.

**Supplementary Information:**

The online version contains supplementary material available at 10.1038/s41598-025-85351-1.

## Introduction

Hydrogen sulfide (H_2_S) is known as a highly toxic foul-smelling compound, on the other hand, it is an important endogenous gasotransmitter, that plays a critical role in a wide range of biological processes^1^. Endogenous H_2_S is largely produced from cysteine and homocysteine as a metabolite of the transsulfuration pathway by the action of three enzymes, cystathionine β-synthase, cystathionine γ-lyase and 3-mercaptopyruvate sulfur transferase. H_2_S is involved in several signaling patwhways, including the regulation of cell proliferation by inducing apoptosis through increased p53 tumorsupressor gene expression^2^. However, in some cell lines, such as endothelial cells, overproduction of H_2_S can stimulate cell proliferation through kinase activation leading to angiogenesis^3^. Low H_2_S levels can be observed in some neurological diseases (Parkinson’s or Alzheimer’s disease) demonstrating the neuromodulating effect of hydrogen sulfide^4^. H_2_S has been shown to have anti-inflammatory and immunomodulating effects, and to protect against multiple stresses, including oxidative stress^4–6^. It can induce vasodilation by activating potassium channels^7^. H_2_S shows significant activity against numerous fungi, including the human pathogen *C. albicans*^8^, and also inhibits the replication of a wide range of RNA viruses, such as the causative agent of COVID-19, SARS-CoV-2, presumably by preventing the pathogens from interacting with their receptors on the cell surface^9–12^.

Based on these various biological effects, H_2_S is a promising molecular entity in the prevention and treatment of several diseases. However, administering a highly toxic and extremely unpleasant-smelling gas poses a real challenge. Therefore, it is preferable to use H_2_S donor molecules rather than hydrogen sulfide itself. The simplest of such compounds are inorganic sulfide, polysulfide and hydrogene-sulfide salts, although the H_2_S releasing profile of these compounds is not ideal as they release a high amount of H_2_S in a short period, causing short-lived effect^13^. To overcome this problem, several small-molecule organic H_2_S donors have been developed that release hydrogen upon hydrolysis, endogenous triggers (usually thiols), or external stimuli^13,14^. Some key representatives of organic H_2_S donors include 1,2-dithiol-3-thiones, arylthioamides, aryl isothiocyanates and Lawesson reagent-based molecules such as GYY4137^4,13–15^.

**Fig. 1:**
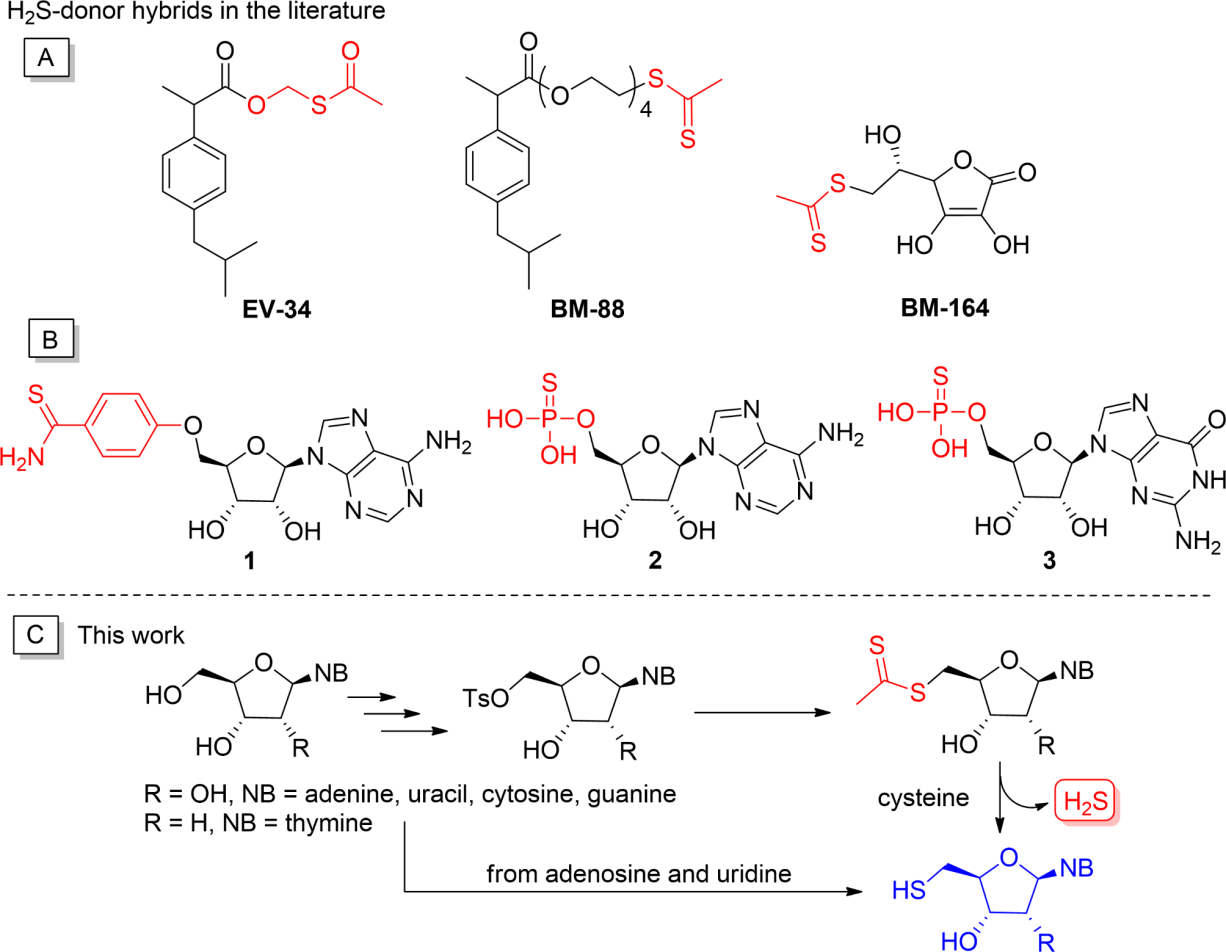
(**A**) H_2_S donors, previously synthesized by our research group; (**B**) H_2_S donor nucleosides in the literature; (**C**) this work: synthesis and characterization of cysteine selective H_2_S donor nucleosides and their metabolites.

The hydrogen sulfide-releasing structures is often conjugated to nonsteroidal anti-inflammatory or other drugs or to different biomolecules to obtain H_2_S-donor hybrid molecules that combine the beneficial therapeutic effects of the parent compound and H_2_S^16–19^. Recently, we synthesized the H_2_S-donor hybrid molecules **EV-34** and **BM-88** from the commonly used nonsteroidal anti-inflammatory drug ibuprofen (Fig. 1A), the former being a formaldehyde O,S-acylal, which is a hydrolysis-based H_2_S donor^14,20^, while **BM-88** is a dithioacetic acid ester that is activated by the endogenous trigger, cysteine^21^. We also produced a dithioacetate-containing H_2_S donor hybrid, **BM-164**, from the well-known antioxidant ascorbic acid (vitamin C)^22^. All three compounds showed excellent hydrogen sulfide-releasing properties, and **BM-88** and **BM-164** also exhibited remarkable cardioprotective effects.

Nucleosides are important biomolecules, and their synthetic analogs are widely used in the anticancer and antiviral chemotherapy^23,24^. Albeit sporadically, nucleosides have already been used to construct hydrogen sulfide donors, which always contain the H_2_S-releasing group in the 5′ position (Fig. 1B). Among them, compound **1** is an aryl-thioamide-type H_2_S donor, which is connected to adenosine with a non-hydrolyzable ether bond^25^, while derivatives **2** and **3** are nucleoside monophosphorothioates, which can be enzymatically converted into AMP and GMP, respectively, while hydrogen sulfide is released^26,27^. Adenosine **1** significantly reduced infarct size when administered at the end of sustained ischemia and showed enhanced cardioprotection compared to adenine^25^. Phosphorothioates **2** and **3** were reported to restore the anti-contractile effects of perivascular adipose tissue in obese rats through efficient H_2_S release^26^.

It is also worth mentioning that 5′-thio-modified nucleosides have significant bioactivity and therapeutic potential. Notably, 5′-thioadenosine derivatives exert significant antiprotozoal activity against *P. falciparum*^28,29^, and 5′-thiothymidine was shown to be a strongly binding allosteric ligand of the SARS-CoV-2 NSP15 endonuclease^30^. 5′-Cysteine and homocysteine derivatives of nucleosides and homonucleosides have been prepared and tested against S-adenosyl-homocysteine hydrolase and also used as monomer building blocks in the synthesis of peptide nucleic acids^31,32^. Moreover, S-adenosylmethionine (SAM) is a well-known biomolecule that serves as a methyl donor in methyl transfer reactions^33^.

Inspired by the literature results, we envisaged the synthesis of new cysteine-selective nucleoside-H_2_S-donor hybrid molecules, which release H_2_S in the body in a controllable manner, while being transformed into the corresponding 5′-thionucleoside (Fig. 1C). In this work, we describe the synthesis, H_2_S-releasing and antioxidant properties as well as antiviral effect against SARS-CoV-2 of the 5′-dithioacetate derivatives of the four canonical nucleosides (uridine, adenosine, cytidine, guanosine) and the deoxynucleoside thymidine. As reference compounds for the biological assays, the putative 5′-thio metabolites were also produced from uridine and adenosine. Given that adenosine plays a critical role in the regulation of heart function, we also studied the cardiac effects of the 5′-dithioacetyl adenosine derivative in a rat model.

## Results and discussion

### Synthesis

**Fig. 2:**
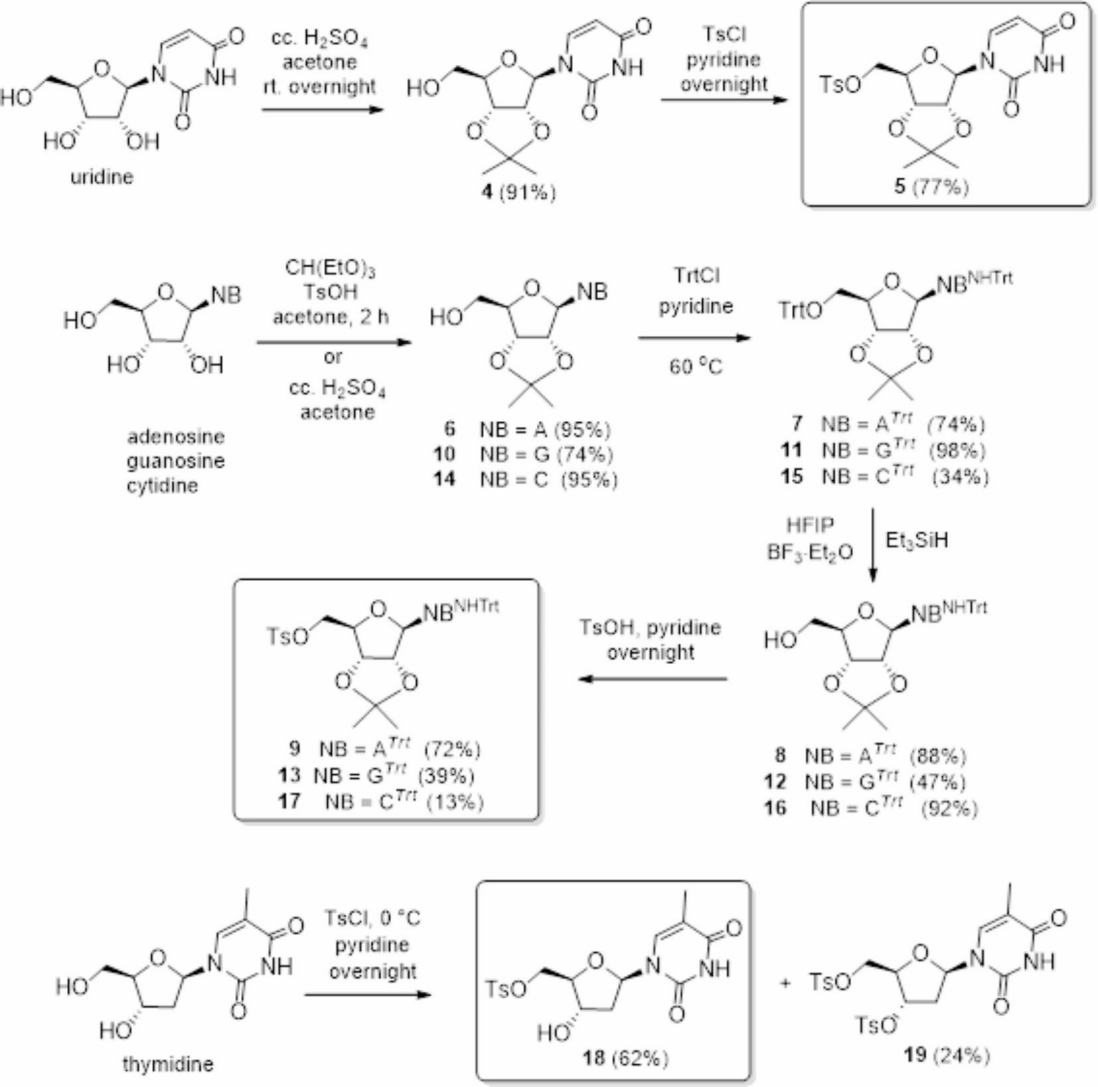
Synthesis of nucleoside 5′-tosylates.

The synthesis of nucleoside 5′-dithioacetates was performed from properly protected nucleosides containing a leaving group in the 5′-position. Tosyl (Ts) was chosen as the leaving group, isopropylidene acetal was used to protect the 2′ and 3′ hydroxyl groups, while the exocyclic amino group of adenosine, guanosine and cytidine was protected with a triphenylmethyl (Trt) group (Fig. 2). Uridine was converted to 5′-tosylate **5** in two steps by isopropylidenation (**4**) followed by treatment with TsCl in pyridine^34^. Adenosine, guanosine and cytidine were acetalated according to the literature method^35^. Compounds **6**, **10** and **14** were *O*,*N*-ditritylated, followed by selective *O*-detritylation, using a cocktail of hexafluoroisopropanol (HFIP), and boron trifluoride diethyl etherate (BF_3_.Et_2_O) as Brönsted and Lewis acids, respectively, and triethylsilane (Et_3_SiH) as reducing agent^36^. The free 5′-OH of the resulting **8**, **12** and **16** was tosylated to obtain the fully protected 5′-tosylates **9**^32^, **13** and **17**.

The required tosylate was prepared from the deoxynucleoside thymidine in one step without the use of any protecting group. By reacting thymidine with TsCl in pyridine at room temperature, only the 5′,3′-ditosylate was obtained. Repeating the reaction at 0 °C, the desired 5′-*O*-monotosyl derivative (**18**)^37^ was formed as the main product.

**Fig. 3:**
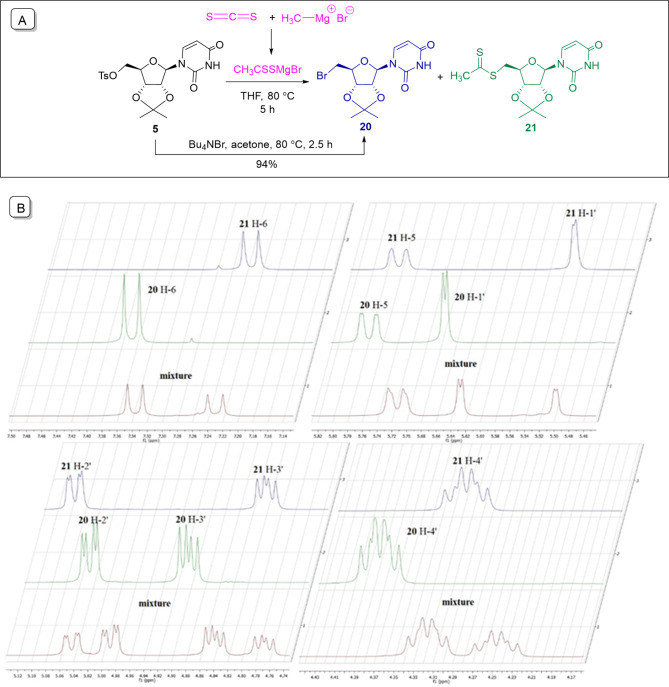
(**A**) Synthesis of the uridine dithioacetate **21** by freshly prepared dithioacetate salt CH_3_CSSMgBr; (**B**) Comparison of the characteristic NMR signals of the ^1^H spectra of dithioacetate **21**, bromouridine **20** and their mixture.

Next, the dithioacetate group was introduced into the 5′-position of the nucleoside using a one-pot reaction. First, the Mg salt of dithioacetic acid was produced in situ by the reaction of carbon disulfide and methyl magnesium bromide, using a modified version of the method described by Maeda et al.^38^ (Fig. 3A). Nucleoside tosylate was then added to the reaction mixture and reacted with the dithioacetate at 80 °C. After 5 h, completion of the reaction and the formation of a uniform product was observed by TLC. Unfortunately, however, NMR spectra showed that the chromatographically uniform product was an inseparable 1:1.4 mixture of the desired 5′-dithioacetate (**21**) and a side product. We assumed that 5′-deoxy-5′-bromouridine (**20**) is formed as an undesired product, and to prove this, the presumed bromo derivative was synthesized from **5**, using tetrabutylammonium bromide at 80 °C. By comparing the NMR spectra of the obtained 5′-bromouridine **20** and the mixture (Fig. 3B), it was confirmed that the impurity is indeed the the 5′-bromo derivative. The rapid conversion of tosylate to bromouridine (2.5 h) and the high proportion of the latter in the mixture after 5 h indicate that the bromo derivative is formed first, which is converted to dithioacetate in a slow reaction. Accordingly, the reaction was repeated with an extended reaction time of 18 h. In this case, only the desired 5′-dithioacetate was obtained with good yield (80%).

Based on the above, nucleoside 5′-tosylates **9**, **13**, **17** and **18** were reacted with 3 equiv. of dithioacetate salt in an 18-hour reaction at 80 °C to obtain the desired **23**, **28** and **30**. In the case of the guanosine derivative, only the 5′-bromoderivative **25** was formed under the above conditions, which could be converted to the desired **26** in 85% yield with a large excess of dithioacetate salt during a 28-hour reaction. Adenosine dithioacetate **23** was formed with CH_3_CSSMgBr in an exceptionally low yield of 28%, which significantly increased to 72% when the reaction was performed with potassium dithioacetate obtained in two steps from the magnesium salt.

**Fig. 4:**
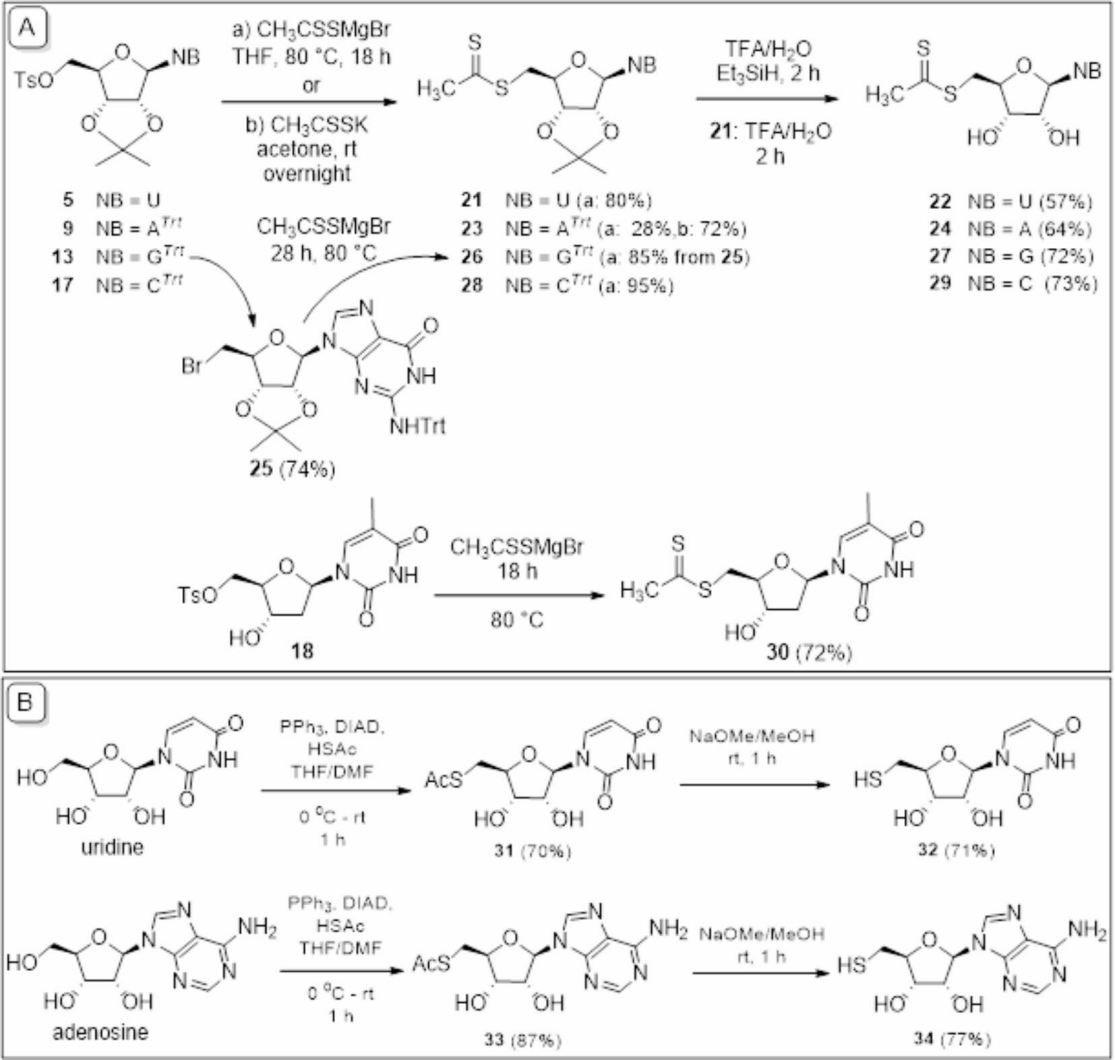
(**A**) Synthesis of nucleoside dithioacetates **22**, **24**, **27**, **29** and **30**; (**B**) synthesis of 5′-thionucleosides **32** and **34**.

The trityl and isopropylidene protecting groups were removed in one step with 90% trifluoroacetic acid, using triethylsilane as a reducing agent in the presence of trityl group (Fig. 4A).

It is assumed, that in a biological milieu, the synthesized 5′-dithioacetyl nucleosides are converted to the corresponding 5′-thionucleosides triggering by the endogenous thiol cysteine. Thus, the assumed metabolites of 5′-dithioacetyl-adenosine and -uridine were also synthesized for biological study. Uridine and adenosine were converted into 5′-acetylthio derivatives **31** and **33** in a Mitsunobu-like reaction, then the S-acetyl group was cleaved, using sodium methylate to obtain the desired thiols **32** and **34** in good yields (Fig. 4B).

### Evaluation of H_2_S releasing profile of the compounds

**Fig. 5:**
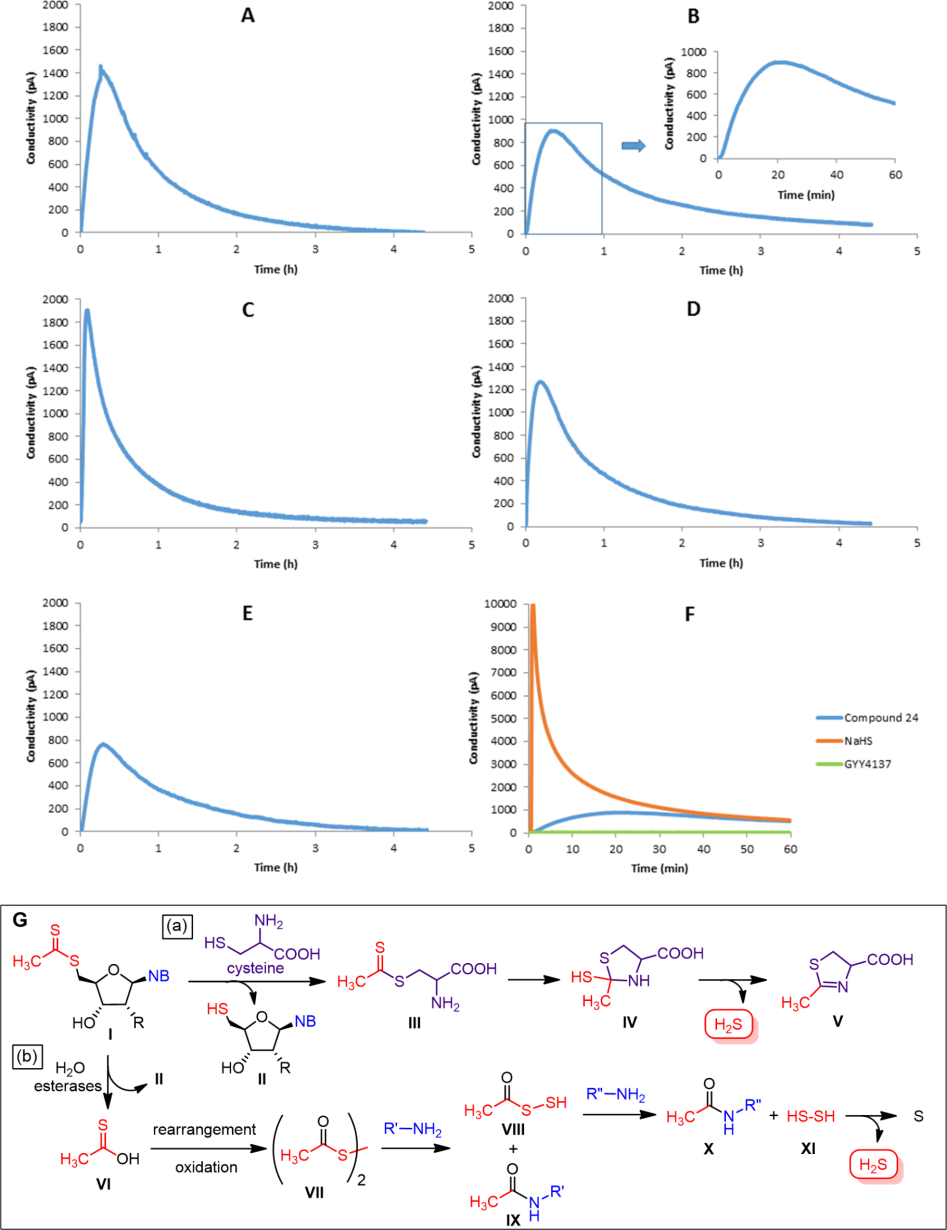
H_2_S releasing curves measured by H_2_S selective sensor (**A**–**F**, 10% w/v) and two possible routes of H_2_S release from 5′-dithioacetyl nucleosides under physiological conditions (**G**). (**A**) Compound **22** (0.31 mM); (**B**) compound **24** (0.29 mM) (inset: first 60 min); (**C**) compound **27** (0.28 mM); (**D**) compound **29** (0.32 mM); (**E**) compound **30** (0.32 mM); (**F**) the blue curve shows the H_2_S release of compound **24** (0.31 mM), the orange curve shows the H_2_S release of NaHS (0.1 mg in 5 mL of distilled water, 0.36 mM) and the green curve shows the H_2_S release of GYY4137 (0.5 mg in 5 mL of medium, 0.27 mM); (**G**) H_2_S release (a) by cysteine-mediated transesterification, (b) by aspecific esterase-catalyzed hydrolysis.

The H_2_S release of compounds **22**, **24**, **27**, **29** and **30** was detected in Dulbecco’s modified Eagle’s medium with an amperometric, H_2_S-specific sensor. The gas release reached the maximum level after 10–20 min and remained at a measurable level even after two hours. On the other hand, the base clearly also has a role in the kinetics of the process. The guanosine derivative **27** releases a large amount of H_2_S relatively quickly, while adenosine and thymidine release H_2_S more slowly than the other nucleosides, and adenosine has the longest-lasting effect (Fig. 5A–E). We also studied the H_2_S emissions of two known H_2_S donors as references, the fast-release NaHS and the slow-release GYY4137 (Fig. 5F). With the latter, there was no measurable H_2_S release, which is in line with the literature data, according to which GYY4137 requires the presence of a high concentration of cysteine (4 mM) for detectable generation of H_2_S^7^. Based on the comparison with the reference compounds, it can be concluded that nucleoside dithioacetates can be considered H_2_S donors with a medium release rate.

Based on literature results, H_2_S is released from dithioester derivatives via a native chemical ligation mechanism mediated by cysteine (Fig. 5G, route a), resulting in thiol (**II**), cysteine-derived dihydrothiazole (**V**) and H_2_S^21^. This mechanism is unlikely to be influenced by the structure of the nucleosides. However, Liu and Orgel showed that dithioacetates can undergo hydrolysis catalyzed by native aspecific esterases^20^. In this reaction pathway, in addition to the thiol, a thioacetic acid is formed, which spontaneously oxidizes to disulfide (**VIII**), and finally the disulfide reacts with native amines to produce acetamide and H_2_S (Fig. 5G, route b). The structure of the nucleosides certainly influences this enzyme-catalyzed process, which is likely to account for the slightly different kinetics of hydrogen sulfide release for different nucleoside structures.

### Antioxidant assays

Oxidative stress arises from an imbalance between increased reactive oxygen species (ROS) production and diminished antioxidant host defenses. Growing evidence suggests that this condition significantly contributes to the pathogenesis of several human diseases, including SARS-CoV-2 infection^39^. With the 5′-dithioacetate derivatives and their synthetic 5′-SH metabolites in hand, the antioxidant activities of compounds **22**, **24**, **32**, and **34** were estimated in the DPPH radical scavenging assay. Furthermore, the ability of the two former dithioacetate derivatives, as well as the H_2_S-donor GYY4137 serving as a positive control, was also evaluated after the addition of a homogenized rat heart extract.

As mentioned above, our newly designed compounds are H_2_S donor nucleoside derivative prodrugs featuring a dithioacetate moiety. These prodrugs undergo cysteine-mediated transesterification^21^ or aspecific esterase-catalyzed hydrolysis^20^, resulting in the release of H_2_S and the corresponding 5′-thionucleoside. In the present study, we utilized a freshly prepared supernatant obtained from the tissue homogenate of a frozen control rat heart in PBS buffer to replicate physiological conditions, thus inducing H_2_S release.

Endogenous antioxidants play a crucial role in enhancing cellular defense against the harmful effects of reactive oxygen species (ROS)^40^. The presence of these antioxidants in the extract used in our study may lead to misinterpretations of the radical scavenging efficacy of the compounds under investigation. To prevent such interference, the extract can only be introduced in a volume that shows no or negligible radical scavenging ability. Therefore, we tested different volumes of the extract, ranging from 50 to 5 µL, in a 200:800:1000 µL mixture of methanol, 0.1 M Tris-HCl buffer, and DPPH. The measurements were carried out immediately and after 30 min of incubation, ensuring no time-dependent behavior. The extract demonstrated inhibition ratios of 6% and 15% against the DPPH^•^ free radical when 25 µL of the extract was utilized immediately and after a 30-minute incubation period, respectively. Conversely, extract volumes ranging from 20 µL to 5 µL yielded minimal scavenging ratios of only 1.5% and 2.5%, immediately and after 30 min of incubation, respectively. Therefore, the highest volume, 20 µL, which exhibited no activity at either time point was selected for further experimentation (Fig. S1).

The resulting inhibition ratios of the compounds tested were then compared with those obtained for the precursor uridine or adenosine, as well as the positive controls. Three positive controls were employed in this study: 0.5 mM ascorbic acid solution, along with two H_2_S- releasing molecules — sodium hydrosulfide (NaHS), which releases H_2_S rapidly, and the slow-releasing organic H_2_S donor GYY4137.

**Fig. 6:**
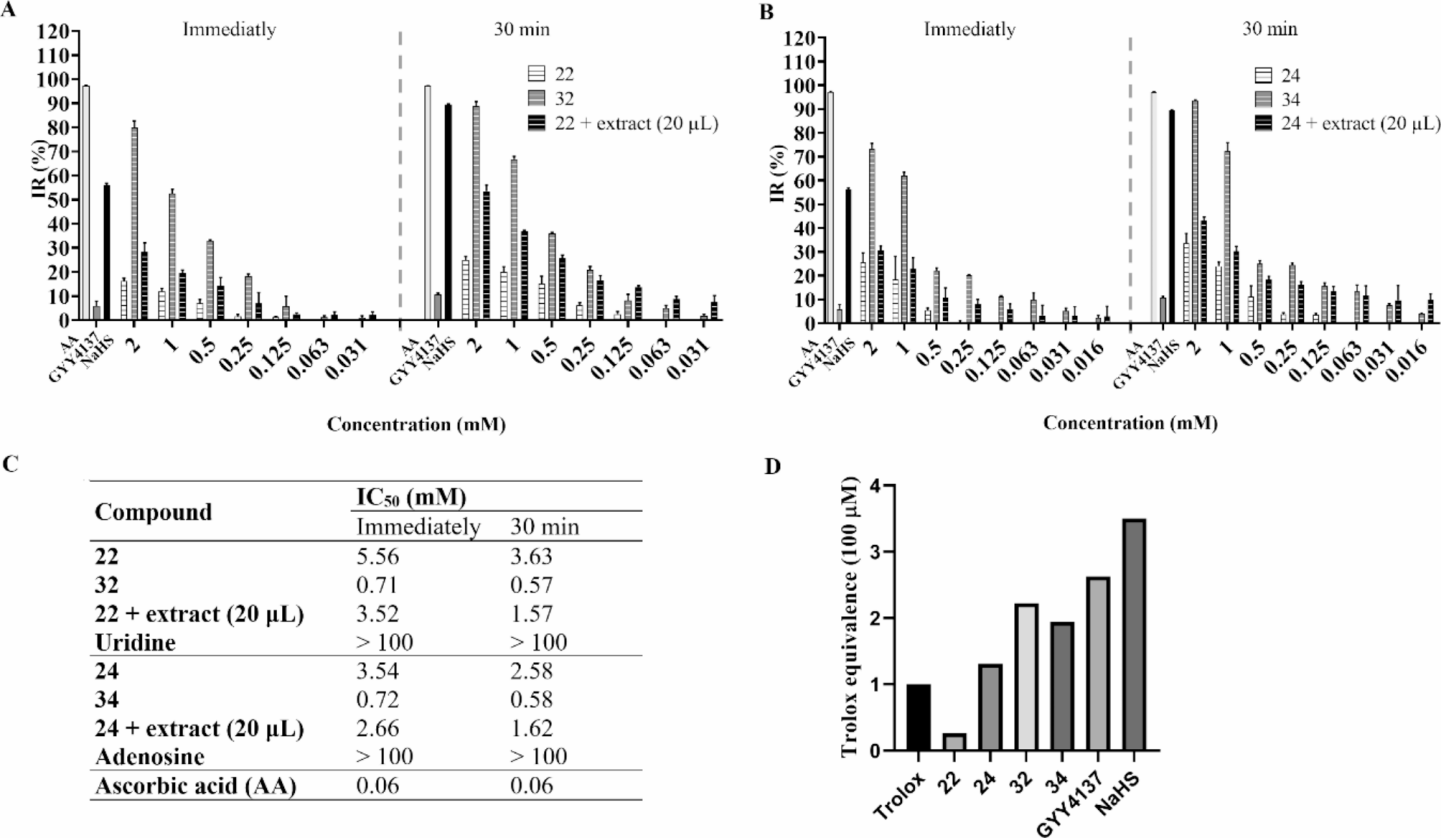
DPPH inhibition ratio of (**A**) compounds **22**, **32**, and **22 + extract (20 µL)**, and (**B**) compounds **24**, **34**, and **24 + extract (20 µL)** immediately and after 30 min of incubation, compared to the positive controls (0.5 mM ascorbic acid (AA, IR = 97.25% and 97.13%), 0.5 mM GYY4137 (IR = 5.95% and 10.63%), and 0.5 mM NaHS (IR = 56.14% and 90.03%), immediately and after 30-min incubation, respectively). Error bars symbolize the standard deviation from the mean value (*n* = 3). Results express mean ± SD. IR = inhibition ratio. (**C**) IC_50_ values (mM) of synthetic compounds compared to the corresponding unmodified nucleosides and ascorbic acid (AA). (**D**) ABTS (2,2′-azinobis-(3-ethylbenzothiazoline-6-sulfonic acid) radical cation decolorization assay of compounds **22**, **24**, **32**, **34** GYY4137, and NaHS. Values are expressed as Trolox equivalents (a vitamin E analogue). Error bars symbolize the mean ± SEM (*n* = 9).

Figure 6 depicts the correlations between the concentrations (mM) and DPPH inhibition ratios (%) of the compounds tested immediately and after 30 min of incubation. Remarkable differences in the inhibition ratios were observed in both time- and concentration-dependent manners.

As shown in Fig. 6, immediate antioxidant activity was observed in all compounds. The 5′-SH metabolites **32** and **34** demonstrated notably higher antioxidant activity than the corresponding 5′-dithioacetate derivatives (**22** and **24**), achieving an inhibition ratio of 70–80% and 90% immediately and after 30 min incubation, respectively, when employed at a concentration of 2 mM.

0.5 mM NaHS or GYY4137 resulted in the release of detectable amounts of H_2_S, as evidenced by their DPPH inhibition ratios recorded immediately and after a 30-minute incubation period. The release of H_2_S from NaHS occurred rapidly, reaching a DPPH inhibition ratio of 90% at the 30-minute mark. In contrast, the release of H_2_S from GYY4137 was significantly lower, but sustained throughout the incubation duration, as demonstrated by the increase in the inhibition ratio.

Notably, the dithioacetate derivatives **22** and **24** with 20 µL of the homogenized myocardial extract showed a higher antioxidant activity than without the extract, and surpassed that of the 5′-SH metabolite at lower concentrations after 30 min of incubation, clearly demonstrating that the DPPH inhibitory activity of the dithioacetate derivatives in the presence of the extract continuously increases during the 30-min incubation time due to the sustained concurrent release of two antioxidant molecules: the 5′-SH metabolite and H_2_S.

The IC_50_ values of the synthesized compounds deviated significantly from that belonging to the reference compounds, with the 5′-SH analogues **32** and **34** as the most active. However, the observed activity was 10 times lower than that achieved with ascorbic acid.

It should be noted that 20 µL triggering extract was also introduced to the vitamin C and compounds **24** and **34** groups. The degree of discoloration of the free radical was monitored over a duration of 30 min. The extract demonstrated negligible or only minimal negative effects on the inhibition ratios of these groups, which were primarily noted at high concentrations. This finding indicates that the extract acts mainly on the dithioacetate group.

Furthermore, we compared the antioxidant activity of dithioacetate derivatives and 5′-SH metabolites using ABTS (2,2′-azinobis-(3-ethylbenzothiazoline-6-sulfonic acid)) assay, which showed a similar activity pattern to the DPPH assay. Among the molecules the 5′-SH metabolites **32** and **34** showed higher antioxidant activity than the parent dithioacetate derivatives **22** and **24**. (Fig. 6, panel D).

### Cell viability

In order to evaluate the biocompatibility of the new compounds, MTT assays were carried out in the presence of different concentrations of 5′-dithioacetate derivatives and their synthetic 5′-SH metabolites on H9c2 cells. None of the molecules showed cell toxicity in a physiologically relevant concentration range (Fig. S2).

### Antivirals results

To determine H_2_S releasing effect of nucleoside 5′-dithioacetates on SARS-CoV-2 replication, we added compounds to Calu-3 lung epithelial cells two hours after infection and washing virus away. As the release of H_2_S is relatively short-lived we evaluated anti-SARS-CoV-2 effect of compounds already two days after infection in cytopathic-effect-based assay using sensitive CellTiter-Glo luminescent cell viability assay. Only compounds **24** and **27** exhibited moderate anti-SARS-CoV-2 activity in Calu-3 cells with an EC_50_ of 173 ± 24 µM and 158 ± 34 µM, respectively (Table 1). Next, the virus yield reduction assay was performed to ascertain if there is direct effect on the released SARS-CoV-2 virions into media. The medium after two days infection of Calu-3 cells was analyzed in Vero E6 cells using plaque assay. Only compound **27** very slightly reduced the SARS-CoV-2 titer by 0.38 log_10_ PFU/mL.

**Table 1:** Anti-SARS-CoV-2 activities (EC_50_), cytotoxicity in Calu-3 cells (CC_50_) and virus yield reduction assay for nucleoside 5′-dithioacetates.

Compound	EC_50_ ± SE [µM]	CC_50_ ± SE [µM]	SARS-CoV-2 titer reduction [log_10_ PFU/mL]
**22**	> 1000	> 1000	0.02
**24**	173 ± 24	~ 1000	0
**27**	158 ± 34	> 1000	0.38
**29**	> 1000	> 1000	0.13
**30**	621 ± 181	764 ± 82	0.01
Remdesivir	0.5 ± 0.26	> 10	7.1

### Results of in vivo experiments

The cardiac effects of 5′-dithioacetyl uridine and adenosine (**22** and **24**), 5′-thioadenosine (**34**) and adenosine as a reference compound were evaluated in rat model. Adenosine at a dose of 2 mg/kg induced heart block, and heart rate (HR) remained significantly lower 30 s post-administration compared to baseline; however, this negative chronotropic effect dissipated within approximately 60 s. None of the new compounds (**22**, **24**, and **34**) induced complete heart block or pauses, but adenosine derivatives **24** and **34** significantly reduced HR relative to baseline values (*p* = 0.0162; and *p* = 0.0010, respectively). Although the negative chronotropic effect of compounds **24** and **34** was less pronounced than that of adenosine, their duration of action was longer. The uridine-based compound **22** did not produce notable changes in HR, though a non-significant reduction was observed (*p* = 0.0562).

**Fig. 7:**
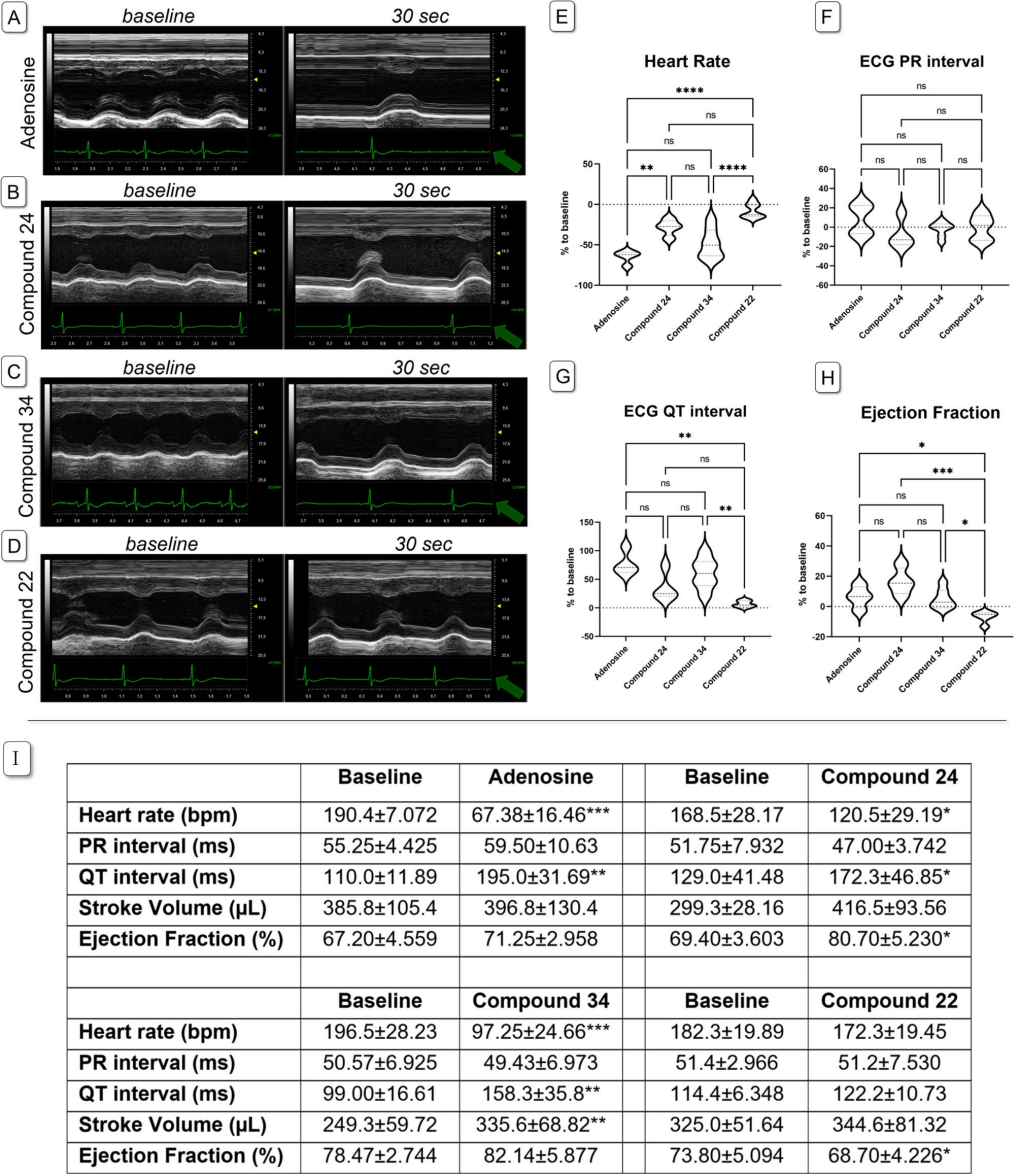
Comparison of the cardiac effects of adenosine (*n* = 4), compound **24** (*n* = 4), **34** (*n* = 7) and **22** (*n* = 5) on anaesthetized SD rats. Panels (**A**–**D)** show M-mode traces of the left ventricle, obtained from PSAX view at baseline and 30 s after drug administration (sweep speed adjusted to 60 0 Hz). Compound **24** and **34** caused similar but smaller effect than the parent molecule adenosine. Ejection fraction (panel **H**) increased after compound **24** administration but slightly decreased under the influence of compound **22**. Heart rate decreased after adenosine, compound **24** and **34** administration, but only slightly changed after compound 22 injection (panel **E**). ECG PR interval was unchanged in all cases (panel **F**). A significant QT interval prolongation on the ECG was observed after the administration of all adenosine-based compounds, but not after the uridine-based (panel **G**). One-way ANOVA with Tukey post-test was used to compare % changes of the parameters in the treatment groups. Asterisks denote the level if significance: *p < 0.05; **p < 0.01; ***p < 0.001. Green arrows denote lead II ECG traces. Panel (**I**) Effect of adenosine, compound **24**, **34** and **22** on ECG and echocardiographic parameters, at the baseline and 30 s after i.v. drug administration (2 mg/kg, i.v.). Adenosine-based compounds significantly decreased heart rate with a marked QT prolongation, while increased SV and EF. All data is presented as mean ± SD. Asterisks denote the level if significance: *p < 0.05; **p < 0.01; ***p < 0.001 (paired t-test, vs. baseline values).

Neither the new compounds nor adenosine significantly affected the ECG PR interval in anesthetized rats. Adenosine significantly prolonged the QT interval (*p* = 0.0062 vs. baseline), and both compounds **24** and **34** similarly extended the QT interval compared to their corresponding baseline values (*p* = 0.0035 and *p* = 0.0034, respectively), akin to the parent compound adenosine. Compound **22** did not alter PR or QT intervals but slightly decreased ejection fraction (EF) 30 s after administration (*p* = 0.0208 vs. baseline). Adenosine and compound **34** tended to increase EF (*p* = 0.189 and *p* = 0.0757 vs. baseline, respectively), whereas compound **24** significantly increased EF (*p* = 0.0302 vs. baseline). Interestingly, stroke volume (SV) remained unchanged following administration of adenosine or compound **22**, but both compounds **24** and **34** increased SV (*p* = 0.0811 and *p* = 0.0068, respectively; Fig. 7).

## Conclusions

By optimizing the synthesis steps, we developed an efficient route for the synthesis of new hydrogen sulfide donor nucleosides containing a dithioacetyl group. Using amperometric, H_2_S-specific sensor measurements, we established that dithioacetate nucleosides are medium-speed H_2_S donors, and nucleobases also have an effect on the kinetics of hydrogen sulfide release. DPPH and ABTS assays were used to determine the antioxidant profile of the hydrogen sulfide donor uridine and adenosine and their 5′-thio metabolites.

In the antiviral tests against SARS-CoV-2, both adenosine and guanosine derivatives **24** and **27** showed an antiviral effect, but surprisingly, only the fastest hydrogen sulfide-releasing guanosine derivative **27** measurably reduced the virus titer. According to literature results, H_2_S can suppress infections caused by enveloped viruses, including SARS-CoV-2, through several mechanisms, one of the most important of which is the induction of long-term antioxidant activity^11,41^. H_2_S can directly activate the Nrf2 transcription factor pathways, and also contributes to antioxidant activity through various indirect effects, such as maintaining increased levels of an important antioxidant compound, glutathione^41,42^ Glutathione has been shown to have therapeutic potential against COVID-19, being able to reduce disulfide bonds in the SARS-CoV-2 spike protein and its human receptor ACE-2, which disrupts the spike protein ACE2 interaction, preventing viral entry^43,44^ Until now, mostly slow-release hydrogen sulfide donors (e.g. Na_2_S_2_O_3_ and GYY4137) have been shown to have anti-SARS-CoV-2 effects^9–11^, but the role of the hydrogen sulfide release rate in the antiviral effect has not been investigated. Therefore, literature results do not provide evidence that the antiviral activity of the guanosine derivative **27** is related to its slightly faster sulfide-releasing kinetics compared to other nucleosides or is due to other factors, which requires further investigation.

In the in vivo experiments, administration of 2 mg/kg adenosine resulted in a significant reduction in heart rate (negative chronotropy) accompanied by a marked prolongation of the ECG QT interval, consistent with literature reports^45,46^. Interestingly, the effects of the novel adenosine-based compounds **24** and **34** were less pronounced in terms of negative chronotropy and QT prolongation when administered at the same dose (2 mg/kg). However, the heart rate-lowering effect of compound **34** did not significantly differ from that of adenosine. Notably, the duration of the negative chronotropic effect was longer for compounds **24** and **34** compared to the parent compound. The ECG PR interval, indicative of AV nodal conduction, remained unchanged following drug administration. The uridine-based compound **22** produced a non-significant reduction in heart rate without altering other ECG parameters. Regarding left ventricular systolic parameters, compound **22** slightly decreased ejection fraction. Interestingly, both compounds **24** and **34** increased left ventricular ejection fraction and stroke volume, an effect not observed with adenosine. Adenosine induced heart block but did not significantly affect ejection fraction or stroke volume during the rapid recovery phase, consistent with existing literature^47^. This phenomenon can be explained by the interplay of two effects: the bradycardic effect, which enhances ventricular filling and subsequently increases stroke volume via the Frank-Starling mechanism, and the minimal negative inotropic effects of adenosine, which result in a relatively unchanged ejection fraction when these effects are considered together. In addition, previous in vivo studies have also shown that adenosine alone does not alter ventricular contractility, corroborating our current findings^48^. Additionally, the bradycardic action of adenosine dissipated rapidly (in seconds), further elucidating the non-significant increases observed in ejection fraction and stroke volume. In contrast, the more pronounced increases in stroke volume and ejection fraction associated with the adenosine-based compounds **24** and **34** may be attributed to a more long-lasting reduction in heart rate. During physiological conditions, the Frank-Starling mechanism allows the heart to maintain cardiac output by increasing stroke volume at lower heart rates; thus, we propose that in the case of these two adenosine derivatives, the Frank-Starling mechanism is the predominant factor. Furthermore, according to literature, the release of hydrogen sulfide (H_2_S) demonstrates vasodilatory effects, potentially contributing to enhanced cardiac blood flow, increased venous return, and subsequent improvements in ejection fraction (EF) and stroke volume (SV)^49^. These effects, however, were not observed with the uridine-based compound **22**, which releases H_2_S rapidly (based on our current data) but probably lacks binding affinity for adenosine receptors^50^. This distinction was evident in our in vivo findings, where compound **22** did not significantly influence heart rate, atrioventricular (AV) nodal conduction, SV, or EF. Based on these results, we hypothesize that the observed alterations in EF and SV with adenosine-based compounds are likely secondary to the bradycardia elicited by their mechanism of action, consistent with the effects of the parent molecule, adenosine. Future research could involve a more detailed mechanistic evaluation, examining the compound′s effects at varying doses and dosing intervals while employing specific inhibitors of adenosine receptors and transporters. The distinct cardiac effects associated with adenosine-based compounds underscore the need for further investigation to elucidate their mechanisms and potential therapeutic applications.

## Experimental methods

### General informations

Compounds **5**^34^, **9**^32^, **10**^35^, **14**^35^ and **18**^37^ were prepared according to literature procedures. Nucleosides and reagents were purchased from Sigma-Aldrich Chemical Co. and used without further purification. The optical rotation values of the nucleoside derivatives were determined on a Perkin-Elmer 241 automatic polarimeter. The reactions were monitored by thin-layer chromatography (TLC) on Kieselgel 60 F254 (Merck), detection was done with UV light (254 nm) and by immersion in sulfuric acid ammonium molybdate solution or 5% ethanolic sulfuric acid, followed by heating. Purification was performed by flash chromatography on a silica gel 60 column (Merck, 0.040–0.063 mm). The organic solutions were dried over over anhydrous Na_2_SO_4_ or MgSO_4_, and solvents were evaporated in vacuo. The NMR and MS analysis of the compounds was performed as previously described^32,34^. Briefly, ^1^H NMR (360 and 400 MHz) and ^13^C NMR (90 and 100 MHz) spectra were recorded with Bruker DRX-360 and Bruker DRX-400 spectrometers at 25 °C, chemical shifts are referenced to Me_4_Si (0.00 ppm 1H) and residual solvent signals (CDCl_3_: 77.2, DMSO-d_6_: 39.5, CD_3_OD: 49.0 for ^13^C). Two-dimensional COSY and ^1^H–^13^C HSQC experiments were used to assist NMR assignments. MALDI-TOF MS analyses of the compounds were carried out in the positive reflectron mode using a Bruker Autoflex Speed mass spectrometer equipped with a time-of-flight (TOF) mass analyzer (Bruker, Germany). 2,5-Dihydroxybenzoic acid (DHB) was used as matrix and F_3_CCOONa as cationising agent in DMF.

### Synthesis of the compounds

**2**′,**3**′-***O*-Isopropylidene-5**′-***O*-tosyl-2-*N*-trityl-guanosine (13)**: Compound **12** (1.5 g, 2.65 mmol) was dissolved in dry pyridine (20 mL), TsCl (657 mg, 3.45 mmol, 1.3 equiv.) was added and stirred at rt overnight. Because of the low conversion, another portion of TsCl (657 mg, 3.45 mmol, 1.3 equiv.) was added and stirred for another night. Next day, the reaction mixture was evaporated, the residue was dissolved in CH_2_Cl_2_ (350 mL) and extracted with 10% aq. Na_2_HSO_4_ solution and brine. The organic phase was dried over Na_2_SO_4_, filtered and evaporated under reduced pressure. The crude product was purified by flash column chromatography (gradient elution CH_2_Cl_2_/MeOH 98:2 →97:3→95:5) to give **13** (558 mg, 29%) as a white foam. [α]_D_ = + 19.05 (c = 0.21, CHCl_3_), Rf = 0.17 (CH_2_Cl_2_/MeOH 95/5), ^1^H NMR (400 MHz, CDCl_3_) δ (ppm) 7.77 (s, 1 H), 7.64 (d, *J* = 8.3 Hz, 2 H), 7.29 (dd, *J* = 15.7, 7.8 Hz, 8 H), 7.12 (dt, *J* = 23.7, 7.1 Hz, 9 H), 6.96 (s, 1 H), 5.36 (d, *J* = 2.1 Hz, 1 H), 4.85–4.80 (m, 1 H), 4.18 (d, *J* = 4.7 Hz, 2 H), 3.85 (dd, *J* = 10.7, 3.6 Hz, 1 H), 3.74 (dd, *J* = 10.7, 6.2 Hz, 1 H), 2.41 (s, 3 H, Ts C*H*_3_), 1.43, 1.19 (2 x s, 2 × 3 H, *i*-propylidene C*H*_3_). ^13^C NMR (100 MHz, CDCl_3_) *δ* (ppm) 158.6 (1 C, C-6), 151.5, 149.7, 145.6, 144.5, 132.3 (7 C, C-2, C-4, Ts C-1, Ts C-4, 3 x Trt arom *C*_q_), 130.1, 128.9, 127.9, 127.9, 127.1 (19 C, arom.), 118.4 (1 C, C-5), 114.3 (1 C, *i*-propylidene *C*_q_), 90.7 (1 C, C-1′), 83.3, 82.3, 80.9 (3 C, C-2′, C-3′, C-4′), 71.3 (1 C, N-Trt*C*_q_), 68.9 (1 C, C-6′), 27.3, 25.9 (2 C, 2 x *i*-propylidene *C*H_3_), 21.8 (1 C, Ts *C*H_3_). MALDI ToF MS: m/z calcd for C_39_H_37_N_5_NaO_7_S [M + Na]^+^ 742.231, found 742.241.

**2**′**,3**′**-O-Isopropylidene-O, N-ditrityl-cytidine (15)**: 2′,3′-*O*-Isopropylidene-cytidine **14** (0.927 g, 3.27 mmol) was dissolved in dry pyridine (10 mL). TrtCl (3.7 g, 13.08 mmol, 4.0 equiv.) was added and the rection mixture was stirred at 60 °C for 3 days. Since the conversion was not complete, another portion of TrtCl (462 mg, 0.5 equiv.) and DMAP (20 mg, 0.05 equiv) was added and the solution was stirred at 60 °C for 4 days. The solvent was evaporated *in vacuo*. The crude product was purified by flash column chromatography (gradient elution CH_2_Cl_2_→ CH_2_Cl_2_:MeOH 98:2) to give **15** (1.30 g, 39%) as a white solid. [α]_d_ = − 8.75 (c = 0.40, CHCl_3_), Rf = 0.63 (CH_2_Cl_2_/MeOH 98/2), ^1^H NMR (400 MHz, CDCl_3_) *δ* (ppm) 8.58 (d, *J* = 4.2 Hz, 1 H, N*H*), 7.35–7.09 (m, 31 H, arom.), 6.89 (s, 1 H, H-6), 5.90 (d, *J* = 1.6 Hz, 1 H, H-1′), 4.87 (dd, *J* = 6.2, 1.7 Hz, 1 H, H-2′), 4.77 (d, *J* = 3.4 Hz, 1 H, H-5), 4.75 (d, *J* = 4.5 Hz, 1 H, H-3′), 4.29 (dd, *J* = 7.0, 4.1 Hz, 1 H, H-4′), 3.44 (dd, *J* = 10.7, 4.6 Hz, 1 H), 3.36 (dd, *J* = 10.7, 2.4 Hz, 1 H), 1.53, 1.32 (2 x s, 2 × 3 H, 2 x *i*-propylidene C*H*_3_). ^13^C NMR (100 MHz, CDCl_3_) δ (ppm) 165.65 (1 C, C-4), 155.00 (1 C, C-2), 143.92, 143.24 (6 C, arom. *C*_q_), 141.90 (1 C, C-6), 128.69, 128.66, 128.36, 127.90, 127.57, 127.21 (30 C, arom.), 113.78 (1 C, *i*-propylidene *C*_q_), 87.27 (1 C, O-Trt *C*_q_), 94.47, 93.36, 86.43, 85.76, 80.50 (5 C, C-1′, C-2′, C-3′, C-4′, C-5), 70.85 (1 C, N-Trt *C*_q_), 63.78 (1 C, C-5′), 27.32, 25.48 (2 C, 2x *i*-propylidene *C*H_3_), MALDI-ToF MS: *m/z* calcd for C_50_H_45_N_3_NaO_5_ [M + Na]^+^ 790.33, found 790.43.

**2**′**,3**′-***O*-Isopropylidene-*N*-trityl-cytidine (16)**: Compound **15** (500 mg, 0.651 mmol) was added to the mixture of HFIP (6.5 mL), Et_3_SiH (395 µL, 2.47 mmol, 3.8 equiv.) and BF_3_.Et_2_O (10.4 µL, 0.0846 mmol, 0.13 equiv). When the yellow colour of the solution was disappeared the reaction was monitored by TLC (hexane/acetone 65/35 *R*_*f*_=0.19). After complete conversion of the starting compound (1 h) the reaction was quenched by saturated aq. solution of NaHCO_3_ (0.5 mL). The solvent was evaporated in vacuo and the residue was purified by flash column chromatography (gradient elution hexane: acetone 65:35→6:4→1:1) to give **16** (315 mg = 92%) as a white solid. [α]_d_ = -46.0 (c = 0.25, CHCl_3_), Rf = 0.19 (hexane/acetone 65/35), ^1^H NMR (400 MHz, CDCl_3_) *δ* (ppm) 7.41–7.15 (m, 15 H, arom.), 7.02 (d, *J* = 7.5 Hz, 1 H, H-6), 5.09 (d, *J* = 2.7 Hz, 1 H, H-1′), 5.05 (d, *J* = 7.5 Hz, 1 H, H-5), 4.38 (s, 0.5 H), 4.27 (d, *J* = 2.2 Hz, 1 H), 3.88 (d, *J* = 12.2 Hz, 1 H), 3.75 (d, *J* = 10.6 Hz, 1 H), 1.52, 1.33 (2 x s, 2 × 3 H, 2 x *i*-propylidene C*H*_3_). ^13^C NMR (100 MHz, CDCl_3_) *δ* (ppm) 166.02 (1 C, C-4), 155.22 (1 C, C-2), 144.73 (1 C, C-6), 143.68 (3 C, arom *C*_q_), 128.67, 128.50, 127.75 (15 C, arom.), 113.84 (1 C, *i*-propylidene *C*_q_), 99.07, 94.98, 87.81, 83.32, 80.54 (C-1′, C-2′, C-3′, C-4′, C-5), 71.07 (1 C, N-Trt*C*_q_), 62.83 (1 C, C-5′), 27.27, 25.24 (2 C, 2x *i-*propylidene *C*H_3_). MALDI-ToF MS: *m/z* calcd for C_31_H_31_N_3_NaO_5_ [M + Na]^+^ 548.22, found 548.38.

**2**′,**3**′***-O*-Isopropyliden-5**′***-O*-tosyl-*N*-trityl-cytidine (17)**: Compound **16** (1.86 g, 3.5 mmol) was dissolved in dry pyridine (10 mL) and TsCl (1.33 g, 7.0 mmol, 2.0 equiv.) was added and stirred overnight. Next day, water was added to the reaction mixture and stirred for an hour. The solvent was evaporated under educed pressure and the residue was dissolved in CH_2_Cl_2_ (300 mL). The organic phase was extracted with satd. aq. NaHCO_3_, 10% aq. NaHSO_4_ and water, then dried over Na_2_SO_4_, filtered and evaporated *in vacuo*. The crude product was purified by flash chromatography (hexane: acetone 75:25) to give **17** (303 mg, 13%) as a white foam. [α]_d_ = + 37.7 (c = 0.13, CHCl_3_), Rf = 0.37 (hexane/acetone 6/4), ^1^H NMR (400 MHz, CDCl_3_) *δ* (ppm) 7.75 (d, *J* = 8.3 Hz, 1 H), 7.56 (d, *J* = 8.1 Hz, 1 H), 7.38–7.14 (m, 23 H), 7.02 (d, *J* = 8.0 Hz, 1 H), 6.92 (d, *J* = 7.6 Hz, 1 H), 5.35 (s, 1 H), 5.07 (s, 1 H), 5.00 (s, 1 H), 4.81 (d, *J* = 6.4 Hz, 1 H), 4.77 (d, *J* = 2.1 Hz, 1 H), 2.42 (s, 3 H, Ts C*H*_3_), 1.48, 1.28 (2 x s, 2 × 3 H, 2 x *i*-propylidene C*H*_3_). ^13^C NMR (100 MHz, CDCl_3_) δ 166.1 (1 C, C-4), 154.6 (1 C, C-2), 144.9 (1 C, C-6), 144.1, 143.8, 142.8, 132.9 (5 C, Ts C-1, Ts C-4, 3 x Trt arom *C*_q_), 129.9, 129.1, 128.7, 128.5, 128.4, 128.0, 127.8, 127.0, 126.2 (19 C, arom.), 113.9 (1 C, *i*-propylidene *C*_q_), 97.8, 94.8, 86.6, 84.7, 81.9 (5 C, C-1′, C-2′, C-3′, C-4′, C-5), 71.1, 70.3 (2 C, C-5′, N-Trt *C*_q_), 27.0, 25.2, 21.7 (3 C, Ts CH_3,_*i*-propylidene *C*H_3_), MALDI-ToF MS: *m/z* calcd for C_38_H_37_N_3_NaO_7_S [M + Na]^+^ 702.22, found 702.40.

**5**′**-Bromo-5**′**-deoxy-2**′**,3**′***-O*-isopropylidene-uridine (20)**^51^: Compound **5** (6.6 g, 15.1 mmol), and Bu_4_NBr (6.3 g 19.6 mmol, 1.3 equiv.) were dissolved in acetone (50 mL) and stirred for 2.5 h at 80 °C. The solvent was evaporated under reduced pressure. The crude product was purified by flash column chromatography (hexane: acetone 7:3) to give **20** (5 g, 94%) as white foam. [α]_d_ = + 12.92 (c = 0.48, CHCl_3_), Rf = 0.22 (hexane/acetone 7/3), ^1^H NMR (400 MHz, CDCl_3_) *δ* (ppm) 10.13 (s, 1 H, NH), 7.37 (d, *J* = 8.0 Hz, 1 H, H-6), 5.78 (dd, *J* = 8.0, 1.2 Hz, 1 H, H-5), 5.67 (d, *J* = 2.1 Hz, 1 H, H-1′), 5.04 (dd, *J* = 6.5, 2.1 Hz, 1 H, H-2′), 4.90 (dd, *J* = 6.5, 3.7 Hz, 1 H, H-3′), 4.38 (td, *J* = 5.8, 3.8 Hz, 1 H, H-4′), 3.69 (dd, *J* = 10.6, 6.4 Hz, 1 H, H-5′a), 3.57 (dd, *J* = 10.6, 5.4 Hz, 1 H, H-5′b), 1.58 (s, 3 H, *i*-propylideneCH_3_), 1.37 (s, 3 H, *i*-propylideneCH_3_). ^13^C NMR (100 MHz, CDCl_3_) *δ* (ppm) 164.0, 150.2 (2 C, C-2, C-4), 142.9 (1 C, C-6), 114.8 (1 C, *i*-propylidene*C*_q_), 102.9 (1 C, C-5), 95.3 (1 C, C-1′), 86.8, 84.5, 83.2 (3 C, C-2′, C-3′, C-4′), 32.4 (1 C, C-5′), 27.1, 25.3 (2 C, 2x *i*-propylidene*C*H_3_). MALDI ToF MS: *m/z* calcd for C_12_H_15_BrN_2_NaO_5_ [M + Na]^+^ 369.006, found 369.010.

***S*-(2**′,**3**′-***O*-Isopropylidene-uridine-5**′**-yl)-dithioacetic acid (21): Method A**: CS_2_ (91 µL, mmol, 3.0 equiv.) was dissolved in dry THF (4 mL) under Ar and MeMgBr 3 M solution in diethyl ether (0.5 mL, 1.5 mmol, 3.0 equiv.) was added and stirred overnight. Next day, compound **5** (219 mg, 0.5 mmol) was added and stirred at 80 °C for 5 h. The solvent was evaporated under reduced pressure and the crude product was purified by flash chromatography (hexane: acetone 85:15→7:3) to give 152 mg of the unseparable mixture of **20** and **21** with approximately 1.4:1 ratio, as a yellow foam. **Method B**: CS_2_ (1.0 mL, 17.1 mmol, 3.0 equiv.) was dissolved in dry THF (46 mL) under Ar and MeMgBr (3 M solution, in diethyl ether, 5.7 mL, 17.1 mmol, 3.0 equiv.) was added and stirred overnight. Next day, compound **5** (2.5 g, 5.7 mmol) was added and stirred at 80 °C for 18 h. The solvent was evaporated under reduced pressure and the crude product was purified by flash chromatography (hexane: acetone 85:15→7:3) to give **21** (1.63 g, 80%) as a yellow foam. [α]_D_ = + 33.0 (c = 0.20, CHCl_3_), Rf = 0.28 (hexane/acetone 7/3), ^1^H NMR (400 MHz, CDCl_3_) *δ* (ppm) 9.92 (s, 1 H, N*H*), 7.23 (d, *J* = 8.0 Hz, 1 H, H-6), 5.76 (d, *J* = 8.0 Hz, 1 H, H-5), 5.53 (d, *J* = 1.4 Hz, 1 H, H-1′), 5.09 (dd, *J* = 6.5, 1.6 Hz, 1 H, H-2′), 4.82 (dd, *J* = 6.4, 4.1 Hz, 1 H, H-3′), 4.32 (td, *J* = 6.6, 4.2 Hz, 1 H, H-4′), 3.72–3.60 (m, 2 H, H-5′ab), 2.86 (s, 3 H, thioAc C*H*_3_), 1.54 (s, 3 H, i-propylidene C*H*_3_), 1.35 (s, 3 H, i-propylidene C*H*_3_). ^13^C NMR (100 MHz, CDCl_3_) *δ* (ppm) 232.1 (1 C, *C*S), 163.8, 150.2 (2 C, C-2, C-4), 143.3 (1 C, C-6), 114.7 (1 C, *i*-propylidene *C*_q_), 102.8 (1 C, C-5), 96.1 (1 C, C-1′), 85.6, 84.7, 83.8 (3 C, C-2′, C-3′, C-4′), 39.4 (1 C, thioAc *C*H_3_), 39.1 (1 C, C-5′), 27.2, 25.3 (2 C, 2 x *i*-propylidene *C*H_3_). MALDI ToF MS: *m/z* calcd for C_14_H_18_N_2_NaO_5_S_2_ [M + Na]^+^ 381.055, found 381.065.

***S*-(Uridine-5**′**-yl)-dithioacetic acid (22)**: Compound **21** (2.64 g, 7.36 mmol) was dissolved in 90% aq. TFA (20 mL) and stirred for 2 h at r.t. The solvent was evaporated under reduced pressure, and co-evaporated with toluene. The crude product was purified by flash column chromatography (CH_2_Cl_2_:MeOH 97.5:2.5→96:4→95:5) to give **22** (1.3 g, 57%) as yellow solid. [α]_D_ = + 22.0 (c = 0.10, DMSO), Rf = 0.60 (CH_2_Cl_2_/MeOH 9/1), ^1^H NMR (400 MHz, MeOD) *δ* (ppm) 7.60 (d, *J* = 8.1 Hz, 1 H, H-6), 5.79 (d, *J* = 4.8 Hz, 1 H, H-1′), 5.74 (d, *J* = 8.1 Hz, 1 H, H-5), 4.28 (t, *J* = 5.2 Hz, 1 H, H-2′), 4.15–4.08 (m, 1 H, H-4′), 4.03 (t, *J* = 5.3 Hz, 1 H, H-3′), 3.76 (dd, *J* = 13.9, 5.1 Hz, 1 H, H-5′a), 3.61 (dd, *J* = 13.9, 7.4 Hz, 1 H, H-5′b), 2.85 (s, 3 H, C*H*_3_). ^13^C NMR (100 MHz, MeOD) *δ* (ppm) 234.2 (1 C, *C*S), 166.0, 152.2 (2 C, C-2, C-4), 142.8 (1 C, C-6), 103.1 (1 C, C-5), 91.9 (1 C, C-1′), 82.7 (1 C, C-4′), 74.5 (1 C, C-1′), 74.1 (1 C, C-3′), 40.2 (1 C, C-5′), 39.5 (1 C, *C*H_3_). MALDI ToF MS: *m/z* calcd for C_11_H_14_N_2_NaO_5_S_2_ [M + Na]^+^ 341.0236, found 341.0231 -.

***S*-(2**′,**3**′-***O*-Isopropylidene-*N*-trityl-adenosine-5**′**-yl)-dithioacetic acid (23): Method A**: CS_2_ (257 µL, 4.26 mmol, 3.0 equiv.) was dissolved in dry THF (10 mL) under Ar and MeMgBr 3 M solution in diethyl ether (1.42 mL, 4.26 mmol, 3.0 equiv.) was added and stirred overnight. Next day, compound **9** (1.0 g, 1.42 mmol) was added and stirred at 80 °C for 18 h. The solvent was evaporated under reduced pressure and the crude product was purified by flash chromatography (hexane: acetone 9:1→8:2) to give **23** (251 mg, 72%) as a yellow foam. **Method B**: Compound **9** (100 mg, 0.142 mmol) was dissolved in acetone (5 mL) and potassium-dithioacetate (37 mg, 0.284 mmol, 2.0 equiv.) was added and stirred at r.t. overnight. The solvent was evaporated and the residue was dissolved in CH_2_Cl_2_ (150 mL) and extracted with H_2_O (3 × 50 mL). The organic phase was dried over Na_2_SO_4_, filtered and evaporated under educed pressure. The crude product was purified by flash chromatography (hexane/acetone 85/15) to give **23** (64 mg, 72%) as a yellow foam. [α]_D_ = + 15.8 (c = 0.12, DMSO), Rf = 0.25 (hexane/acetone 8/2), ^1^H NMR (400 MHz, CDCl_3_) *δ* (ppm) 8.03, 7.83 (2 x s, 2 × 1 H, H-2, H-8), 7.34 (d, *J* = 7.0 Hz, 6 H, arom.), 7.30–7.20 (m, 9 H, arom.), 6.98 (s, 1 H, N*H*), 6.02 (s, 1 H, H-1′), 5.51 (d, *J* = 7.6 Hz, 1 H, H-2′), 5.00 (dd, *J* = 6.2, 3.1 Hz, 1 H, H-3′), 4.43 (dt, *J* = 6.8, 3.2 Hz, 1 H, H-4′), 3.68 (dd, *J* = 13.8, 6.6 Hz, 1 H, H-5′a), 3.58 (dd, *J* = 13.8, 7.2 Hz, 1 H, H-5′b), 2.81 (s, 3 H, thioAc C*H*_3_), 1.57 (s, 3 H, *i*-propylidene C*H*_3_), 1.36 (s, 3 H, *i*-propylidene C*H*_3_). ^13^C NMR (100 MHz, CDCl_3_) *δ* (ppm) 231.9 (1 C, *C*S), 154.4, 148.2, 121.7 (3 C, C-4, C-5, -6), 152.5 (1 C, adenine *C*H), 145.0 (3 C, 3 x Trt arom *C*_q_), 129.1, 128.0, 127.1 (15 C, arom.), 114.7 (1 C, *i*-propylidene *C*_q_), 91.1 (1 C, C-1′), 84.8, 84.23, 84.1 (3 C, C-2′, C-3′, C-4′), 39.3 (1 C, thioAc *C*H_3_), 39.2 (1 C, C-5′), 27.2, 25.4 (2 C, 2 x *i*-propylidene *C*H_3_). MALDI ToF MS: *m/z* calcd for C_34_H_33_N_5_NaO_3_S_2_ [M + Na]^+^ 646.192, found 646.204.

***S*-(Adenosine-5**′**-yl)-dithioacetic acid (24)**: Compound **23** (218 mg, 0.35 mmol) was dissolved in 90% aq. TFA (5 mL) Et_3_SiH (167 µL, 1.05 mmol, 3.0 equiv.) was added and stirred for 2 h at r.t. The solvent was evaporated under reduced pressure, and co-evaporated with toluene. The crude product was purified by flash column chromatography (CH_2_Cl_2_:MeOH 98:2→97:3→95:5) to give **24** (76 mg, 64%) as yellow solid. [α]_D_ = -7.14 (c = 0.14, DMSO), Rf = 0.1 (CH_2_Cl_2_/MeOH 95/5), ^1^H NMR (400 MHz, MeOD) *δ* (ppm) 8.23, 8.20 (2 x s, 2 × 1 H, H-2, H-8), 5.97 (d, *J* = 5.3 Hz, 1 H, H-1′), 4.92 (t, *J* = 5.3 Hz, 1 H, H-2′), 4.31 (d, *J* = 4.3 Hz, 1 H, H-3′), 4.28–4.21 (m, 1 H, H-4′), 3.83 (dd, *J* = 13.9, 5.5 Hz, 1 H, H-5′a), 3.72 (dd, *J* = 13.9, 7.3 Hz, 1 H, H-5′b), 2.82 (s, 3 H, thioAc C*H*_3_). ^13^C NMR (100 MHz, MeOD) *δ* (ppm) 234.3 (1 C, *C*S), 153.9 (1 C, adenine *C*H), 90.4 (1 C, C-1′), 83.5 (1 C, C-4′), 74.6 (2 C, C-3′, C-2′), 40.4 (1 C, C-5′), 39.4 (1 C, thioAc *C*H_3_). MALDI ToF MS: *m/z* calcd for C_12_H_16_N_5_O_3_S_2_ [M + H]^+^ 342.0689, found 342.0689.

**5**′**-Bromo-5**′**-deoxy-2**′,**3**′-***O*-isopropylidene-*N*-trityl-guanosine (25)**: CS_2_ (126 µL, 2.08 mmol, 3.0 equiv.) was dissolved in dry THF (5 mL) under Ar and MeMgBr 3 M solution in diethyl ether (126 µL, 2.08 mmol, 3.0 equiv.) was added and stirred overnight. Next day, compound **13** (500 mg, 0.6976 mmol) was added and stirred at 80 °C for 24 h. The solvent was evaporated under reduced pressure and the crude product was purified by flash chromatography (CH_2_Cl_2_:MeOH 98:2→97:3→96:4→95:5) to give **25** (335 mg, 77%) as a yellowish solid. [α]_D_ = + 39.1 (c = 0.32, CHCl_3_), Rf = 0.3 (CH_2_Cl_2_/MeOH 9/1), ^1^H NMR (400 MHz, CDCl_3_) *δ* (ppm) 11.69 (s, 1 H, N*H*), 7.85 (s, 1 H), 7.31 (t, *J* = 7.7 Hz, 8 H, arom.), 7.16 (dt, *J* = 24.6, 7.4 Hz, 12 H, arom.), 7.04 (s, 1 H, arom.), 5.33 (d, *J* = 3.4 Hz, 1 H, H-1′), 4.59 (dd, *J* = 5.9, 3.5 Hz, 1 H), 4.19–4.10 (m, 2 H), 2.94 (dd, *J* = 10.6, 5.9 Hz, 1 H, H-5′a), 2.82 (dd, *J* = 10.5, 7.6 Hz, 1 H, H-5′b), 1.46 (s, 3 H, *i*-propylidene C*H*_3_), 1.23 (s, 3 H, *i*-propylidene C*H*_3_). ^13^C NMR (100 MHz, CDCl_3_) *δ* (ppm) 158.8, 151.4, 149.7, 118.6 (4 C, C-2, C-4′, C-5, C-6), 144.5 (3 C, 3 x Trt arom *C*_q_), 128.9, 127.8, 126.9 (15 C, arom.), 114.2 (1 C, *i*-propylidene *C*_q_), 90.9, 84.3, 82.6, 81.3 (4 C, C-1′, C-2′, C-3′, C-4′), 71.0 (1 C, Trt *C*_q_), 31.5 (1 C, C-5′), 27.4, 25.9 (2 C, 2 x *i*-propylidene *C*H_3_). MALDI ToF MS: *m/z* calcd for C_32_H_30_BrN_5_NaO_4_ [M + Na]^+^ 650.137, found 650.147.

***S*-(2**′,**3**′-***O*-Isopropylidene-*N*-trityl-guanosine-5**′**-yl)-dithioacetic acid (26)**: CS_2_ (173 µL, 2.86 mmol, 6.0 equiv.) was dissolved in dry THF (6 mL) under Ar and MeMgBr (3 M solution in diethyl ether, 0.95 mL, 2.86 mmol, 6.0 equiv.) was added and stirred overnight. Next day, compound **25** (300 mg, 0.477 mmol) was added and stirred at 80 °C for 28 h. The solvent was evaporated under reduced pressure and the crude product was purified by flash chromatography (CH_2_Cl_2_:MeOH 95:5) to give **26** (259 mg, 85%) as a yellow solid. [α]_D_ = + 39.1 (c = 0.32, CHCl_3_), Rf = 0.3 (CH_2_Cl_2_/MeOH 9/1), ^1^H NMR (400 MHz, DMSO) *δ* (ppm) 10.79 (s, 1 H, N*H*), 7.76 (s, 1 H), 7.72 (s, 1 H), 7.36–7.22 (m, 15 H, arom.), 5.51 (d, *J* = 3.3 Hz, 1 H, H-1′), 4.76 (d, *J* = 5.7 Hz, 1 H), 4.06 (s, 1 H), 4.01 (t, *J* = 7.2 Hz, 1 H), 3.14 (dd, *J* = 13.9, 6.3 Hz, 1 H, H-5′b), 2.99 (dd, *J* = 13.7, 8.3 Hz, 1 H, H-5′a), 2.79 (d, *J* = 13.6 Hz, 3 H, thioAc C*H*_3_), 1.36 (s, 3 H, *i*-propylidene C*H*_3_), 1.20 (s, 3 H, *i*-propylidene C*H*_3_). ^13^C NMR (100 MHz, DMSO) δ (ppm) 156.3, 151.2, 148.9, 118.1 (4 C, C-2, C-4, C-5, C-6), 144.5 (3 C, 3 x Trt arom *C*_q_), 128.4, 127.9, 126.9 (15 C, arom.), 113.4 (1 C, *i*-propylidene *C*_q_), 89.3, 82.8, 81.1, 80.6 (4 C, C-1′, C-2′, C-3′, C-4′), 70.3 (1 C, Trt *C*_q_), 39.2 (1 C, thioAc *C*H_3_), 39.1 (1 C, C-5′), 27.0, 25.7 (2 C, 2 x *i*-propylidene *C*H_3_). MALDI ToF MS: *m/z* calcd for C_34_H_33_N_5_NaO_4_S_2_ [M + Na]^+^ 662.1866, found 662.1876.

***S*-(Guanosine-5**′**-yl)-dithioacetic acid (27)**: Compound **26** (221 mg, 0.35 mmol) was dissolved in 90% aq. TFA (5 mL), Et_3_SiH (167 µL, 1.05 mmol, 3.0 equiv.) was added and stirred for 2 h at r.t. The solvent was evaporated under reduced pressure. The crude product was purified by flash column chromatography (CH_2_Cl_2_:MeOH 9:1→85:15→8:2) to give **27** (89 mg, 72%) as yellow solid. [α]_D_ = -5.45 (c = 0.11, DMSO), Rf = 0.44 (CH_2_Cl_2_/MeOH 8/2), ^1^H NMR (400 MHz, DMSO) *δ* (ppm) 10.80 (s, 1 H, N*H*), 7.90 (s, 1 H), 6.61 (s, 2 H), 5.68 (d, *J* = 6.0 Hz, 1 H, H-1′), 5.57 (d, *J* = 6.1 Hz, 1 H, 2′-O*H*), 5.40 (d, *J* = 4.9 Hz, 1 H, 3′-O*H*), 4.64 (dd, *J* = 11.3, 5.8 Hz, 1 H, H-2′), 4.10–4.05 (m, 1 H, H-3′), 4.02–3.97 (m, 1 H, H-4′), 3.71 (dd, *J* = 13.7, 5.5 Hz, 1 H, H-5′a), 3.59 (dd, *J* = 13.7, 8.0 Hz, 1 H, H-5′b), 2.83 (s, 3 H, thioAc C*H*_3_). ^13^C NMR (100 MHz, DMSO) *δ* (ppm) 233.9 (1 C, *C*S), 156.8, 153.8, 151.4, 116.9 (4 C, C-2, C-4, C-5, C-6), 136.63*, 86.8 (1 C, C-1′), 81.2 (1 C, C-4′), 72.9, 72.6 (2 C, C-2′, C-3′). * Can only be seen in HSQC. MALDI ToF MS: *m/z* calcd for C_12_H_15_N_5_NaO_4_S_2_ [M + Na]^+^ 380.0458, found 380.0454.

***S*-(2**′,**3**′-***O*-Isopropylidene-*N*-trityl-cytidine-5**′**-yl)-dithioacetic acid (28)**: CS_2_ (692 µL, 11.4 mmol, 6.0 equiv.) was dissolved in dry THF (24 mL) under Ar and MeMgBr (3 M solution in diethyl ether, 3.82 mL, 11.4 mmol, 6.0 equiv.) was added and stirred overnight. Next day, compound **17** (1.3 mg, 1.91 mmol) was added and stirred at 80 °C for 18 h. The solvent was evaporated under reduced pressure and the crude product was purified by flash chromatography (CH_2_Cl_2_:MeOH 100:0.5→100:1→100:2) to give **28** (1 g, 95%) as a yellow solid. [α]_D_ = + 37.5 (c = 0.16, CHCl_3_), Rf = 0.3 (hexane/acetone 6/4), ^1^H NMR (400 MHz, CDCl_3_) *δ* (ppm) 7.37–7.27 (m, 10 H, arom.), 7.26–7.20 (m, 7 H, arom.), 6.91 (6.92 (d, *J* = 7.6 Hz, 1 H, H-6), 5.27 (s, 1 H, H-1′), 5.25 (s, 1 H, H-2′), 5.01 (d, *J* = 7.5 Hz, 1 H, H-5), 4.93 (dd, *J* = 6.3, 3.5 Hz, 1 H, H-3′), 4.30 (td, *J* = 6.8, 3.6 Hz, 1 H, H-4′), 3.82–3.77 (m, 1 H, H-5′a), 3.72 (dd, *J* = 13.7, 7.3 Hz, 1 H, H-5′b), 2.83 (s, 3 H, thioAc C*H*_3_), 1.49 (s, 3 H, *i*-propylidene C*H*_3_), 1.32 (s, 3 H, *i*-propylidene C*H*_3_). ^13^C NMR (100 MHz, CDCl_3_) *δ* (ppm) 232.3 (1 C, *C*S), 166.1, 154.6 (2 C, C-2, C-4), 144.5 (1 C, C-6), 143.8 (3 C, 3 x Trt arom C_q_), 128.7, 128.5, 127.7 (15 C, arom.), 113.7 (1 C, *i*-propylidene *C*_q_), 98.6 (1 C, C-1′), 94.7 (1 C, C-5), 86.6 (1 C, C-4′), 84.9, 84.6 (2 C, C-2′, C-3′), 71.1 (1 C, Trt *C*_q_), 39.3, 39.2 (1 C, thioAc *C*H_3_), 29.3 (1 C, C-5′), 26.9, 25.1 (2 C, 2 x *i*-propylidene *C*H_3_). MALDI ToF MS: *m/z* calcd for C_33_H_33_N_3_NaO_4_S_2_ [M + Na]^+^ 622.181, found 622.154.

***S*-(Cytidine-5**′**-yl)-dithioacetic acid (29)**: Compound **28** (1 g, 1.69 mmol) was dissolved in 90% aq. TFA (10 mL), Et_3_SiH (808 µL, 5.07 mmol, 3.0 equiv.) was added and stirred for 2 h at r.t. The solvent was evaporated under reduced pressure, and co-evaporated with toluene. The crude product was purified by flash column chromatography (CH_2_Cl_2_:MeOH 9:1) to give **29** (379 mg, 73%) as yellow solid. [α]_D_ = + 40.8 (c = 0.12, CHCl_3_), Rf = 0.18 (CH_2_Cl_2_/MeOH 9/1), ^1^H NMR (400 MHz, MeOD) *δ* (ppm) 7.76 (d, *J* = 7.7 Hz, 1 H, H-6), 6.06 (d, *J* = 7.6 Hz, 1 H, H-5), 5.80 (d, *J* = 3.9 Hz, 1 H, H-1′), 4.27 (d, *J* = 4.6 Hz, 1 H, H-2′), 4.15 (dd, *J* = 12.7, 5.4 Hz, 1 H, H-4′), 4.01 (t, *J* = 5.6 Hz, 1 H, H-3′), 3.80 (dd, *J* = 13.9, 4.9 Hz, 1 H, H-5′a), 3.63 (dd, *J* = 13.9, 7.6 Hz, 1 H, H-5′b), 2.86 (s, 3 H, thioAc C*H*_3_). ^13^C NMR (100 MHz, MeOD) *δ* (ppm) 234.2 (1 C, *C*S), 165.1 (2 C, C-2, C-4), 144.3 (1 C, C-6), 96.3, 92.9 (2 C, C-1′, C-5), 82.8 (1 C, C-4), 75.2, 74.1 (2 C, C-2′, C-3′), 40.2 (1 C, C-5′), 39.4 (1 C, thioAc *C*H_3_). MALDI ToF MS: *m/z* calcd for C_11_H_15_N_3_NaO_4_S_2_ [M + Na]^+^ 340.0396, found 340.0404.

***S*-(Thymidine-5**′**-yl)-dithioacetic acid (30)**: CS_2_ (457 µL, 7.6 mmol, 3.0 equiv.) was dissolved in dry THF (15 mL) under Ar and MeMgBr 3 M solution in diethyl ether (2.53 mL, 7.57 mmol, 3.0 equiv.) was added and stirred overnight. Next day, compound **18** (1.0 g, 2.52 mmol) was added and stirred at 80 °C for 18 h. The solvent was evaporated under reduced pressure and the crude product was purified by flash chromatography (CH_2_Cl_2_:MeOH 96:4→95:5) to give **30** (568 mg, 72%) as a yellow solid. [α]_D_ = + 42.7 (c = 0.36, DMSO), Rf = 0.44 (CH_2_Cl_2_/MeOH 95/5), ^1^H NMR (400 MHz, DMSO) *δ* (ppm) 11.32 (s, 1 H, N*H*), 7.46 (s, 1 H, H-6), 6.17 (t, *J* = 6.9 Hz, 1 H, H-1′), 5.46 (s, 1 H, O*H*), 4.19 (s, 1 H, H-3′), 3.89 (s, 1 H, H-4′), 3.66 (dd, *J* = 13.6, 5.3 Hz, 1 H, H-5′a), 3.53 (dd, *J* = 13.5, 7.8 Hz, 1 H, H-5′b), 2.85 (s, 3 H, thioAc C*H*_3_), 2.29 (dt, *J* = 13.7, 6.9 Hz, 1 H, H-2′a), 2.15–2.05 (m, 1 H, H-2′b), 1.81 (s, 3 H, thymine C*H*_3_). ^13^C NMR (100 MHz, DMSO) *δ* (ppm) 233.9(1 C, *C*S), 163.9, 150.7 (2 C, C-2, C-4), 136.3 (1 C, C-6), 110.1 (1 C, C-5), 84.2 (1 C, C-1′), 83.2 (1 C, C-4′), 73.1 (1 C, C-3′), 39.6 (1 C, thioAc *C*H_3_), 38.2 (1 C, C-2′), 12.3 (1 C, thymine *C*H_3_). MALDI ToF MS: *m/z* calcd for C_12_H_16_N_2_NaO_4_S_2_ [M + Na]^+^ 339.0444, found 339.0438 -.

**5**′-***S*-Acetyl-5**′**-thiouridine (31)**: PPh_3_ (6.5 g, 24.6 mmol, 2.0 equiv.) was dissolved in dry THF (50 mL) and cooled to 0 °C. DIAD (5.0 mL, 24.6 mmol, 2.0 equiv.) was added dropwise and stirred for 30 min at 0 °C. Uridine (3.0 g, 12.3 mmol) and HSAc (1.8 mL, 24.6 mmol, 2.0 equiv.) were dissolved in dry DMF (50 mL) and added dropwisely to the reaction mixture and stirred for 30 min at 0 °C and 30 min at r.t. The solvent was evaporated under reduced pressure and the crude product was purified by flash column chromatography (gradient elution, CH_2_Cl_2_:MeOH 97.5:2.5→9:1→8:2) to give **31** (2.5 g, 70%) as a pale yellowish solid. [α]_D_ = + 17.8 (c = 0.23, DMSO), Rf = 0.16 (CH_2_Cl_2_/MeOH 95/5), ^1^H NMR (400 MHz, DMSO) *δ* (ppm) 11.36 (s, 1 H, N*H*), 7.63 (d, *J* = 8.1 Hz, 1 H, H-6), 5.73 (d, *J* = 5.6 Hz, 1 H, H-1′), 5.67 (dd, *J* = 8.0, 2.1 Hz, 1 H, H-5), 5.43 (d, *J* = 5.6 Hz, 1 H, 2′-O*H*), 5.29 (d, *J* = 4.5 Hz, 1 H, 3′-O*H*), 4.14 (dd, *J* = 10.2, 5.1 Hz, 1 H, H-2′), 3.87–3.78 (m, 2 H, H-4′, H-3′), 3.27 (dd, *J* = 13.8, 5.0 Hz, 1 H, H-5′a), 3.10 (dd, *J* = 13.8, 7.0 Hz, 1 H, H-5′b), 2.36 (s, 3 H, AcC*H*_3_). ^13^C NMR (100 MHz, DMSO) *δ* (ppm) 194.8 (1 C, Ac*C*O), 163.1, 150.7 (2 C, C-2, C-4), 141.2 (1 C, C-6), 102.2 (1 C, C-5), 88.4, 82.3, 72.3, 72.2 (4 C, C-1′, C-2′, C-3′, C-4′), 31.0 (1 C, C-5′), 30.5 (1 C, Ac*C*H_3_). MALDI-ToF MS: *m/z* calcd for C_11_H_14_N_2_NaO_6_S [M + Na]^+^ 325.0465, found 325.0423.

**5**′**-Thiouridine (32)**^52^: Compound **31** (2.4 g, 8.0 mmol) was dissolved in dry MeOH (50 mL) under Ar, and NaOMe (648 mg, 12 mmol, 1.5 equiv.) was added and stirred at r.t. for 1 h. The reaction mixture was neutralized with Amberlite IR 120 H^+^ ion exchanger resine, filtered off, and evaporated under reduced pressure. The crude product was purified by flash column chromatography (CH_2_Cl_2_:MeOH 9:1) to give **32** (1.47 g, 71%) as a white solid. [α]_D_= -40.8 (c = 0.13, DMSO), Rf = 0.24(CH_2_Cl_2_/MeOH 95/5), ^1^H NMR (400 MHz, DMSO) *δ* (ppm) 11.35 (s, 1 H, N*H*), 7.67 (d, *J* = 8.1 Hz, 1 H, H-6), 5.78 (d, *J* = 6.0 Hz, 1 H, H-1′), 5.67 (d, *J* = 8.1 Hz, 1 H, H-5), 5.41 (d, *J* = 5.8 Hz, 1 H, 2′-O*H*), 5.22 (d, *J* = 5.0 Hz, 1 H, 3′-O*H*), 4.13 (dd, *J* = 11.3, 5.7 Hz, 1 H, H-2′), 3.92 (dd, *J* = 8.8, 4.6 Hz, 1 H, H-3′), 3.85 (dd, *J* = 9.5, 5.8 Hz, 1 H, H-4′), 2.81 (dd, *J* = 13.6, 5.5 Hz, 1 H, H-5′a), 2.73 (dd, *J* = 13.8, 6.0 Hz, 1 H, H-5′b), 2.48 (d, *J* = 19.6 Hz, 1 H, S*H*). ^13^C NMR (100 MHz, DMSO) *δ* (ppm) 172.6, 160.3 (2 C, C-2, C-4), 150.6 (1 C, C-6), 111.7 (1 C, C-5), 97.5, 94.1, 81.9, 81.0 (4 C, C-1′, C-2′, C-3′, C-4′), 35.8 (1 C, C-5′). MALDI-ToF MS: *m/z* calcd for C_9_H_12_N_2_NaO_5_S [M + Na]^+^ 283.0359, found 283.0355.

**5**′**-*S*-Acetyl-5**′**-thioadenosine (33)**: PPh_3_ (6.5 g, 24.6 mmol, 2.0 equiv.) was dissolved in dry THF (50 mL) and cooled to 0 °C. DIAD (5.0 mL, 24.6 mmol, 2.0 equiv.) was added dropwise and stirred for 30 min at 0 °C. Adenosine (3.3 g, 12.3 mmol) and HSAc (1.8 mL, 24.6 mmol, 2.0 equiv.) were dissolved in dry DMF (50 mL) and added dropwisely to the reaction mixture and stirred for 30 min at 0 °C and 90 min at r.t. The solvent was evaporated under reduced pressure and the crude product was purified by flash column chromatography (gradient elution, CH_2_Cl_2_MeOH 91→82) to give **33** (3.47 g, 87%) as a pale yellowish solid. [α]_D_= -23.3 (c = 0.39, DMSO), Rf = 0.33 (CH_2_Cl_2_/MeOH 8/2), ^1^H NMR (400 MHz, DMSO) *δ* (ppm) 8.35, 8.17 (2x s, 2 × 1 H, H-2, H-8), 7.33 (s, 2 H, N*H*_2_), 5.89 (d, *J* = 5.7 Hz, 1 H, H-1′), 5.55 (d, *J* = 6.1 Hz, 1 H, 2′-O*H*), 5.41 (d, *J* = 5.0 Hz, 1 H, 3′-O*H*), 4.80 (dd, *J* = 11.2, 5.7 Hz, 1 H, H-2′), 4.12 (dd, *J* = 8.9, 4.6 Hz, 1 H, H-3′), 3.94 (dd, *J* = 9.2, 7.2 Hz, 1 H, H-4′), 3.36 (dd, *J* = 13.8, 5.7 Hz, 1 H, H-5′a), 3.18 (dd, *J* = 13.5, 7.8 Hz, 1 H, H-5′b), 2.33 (s, 3 H, AcC*H*_3_) ^13^C NMR (100 MHz, DMSO) *δ* (ppm) 195.3 (1 C, Ac*C*O), 153.1 (2 C, C-2, C-8), 156.6, 149.9 (2 C, C-4, C-5), 119.7 (1 C, C-6), 88.0, 83.3, 73.1 (4 C, C-1′, C-2′, C-3′, C-4′), 31.7 (1 C, C-5′), 30.9 (1 C, AcC*H*_3_). MALDI-ToF MS: *m/z* calcd for C_12_H_15_N_5_NaO_4_S [M + Na]^+^ 348.074, found 348.067.

**5**′**-Deoxy-5**′**-thioadenosine (34)**^52^: Compound **33** (3.16 g, 9.7 mmol) was dissolved in dry MeOH (50 mL) under Ar, and NaOMe (787 mg, 14.5 mmol, 1.5 equiv.) was added and stirred at r.t. for 1 h. The reaction mixture was neutralized with AcOH and evaporated under reduced pressure. The crude product was purified by flash column chromatography (CH_2_Cl_2_:MeOH 9:1→8:2) to give **34** (2.11 g, 77%) as a white solid. [α]_D_= -18.3 (c = 0.4, DMSO), Rf = 0.17 (CH_2_Cl_2_/MeOH 9/1), ^1^H NMR (400 MHz, DMSO) *δ* (ppm) 8.37, 8.17 (2 x s, 2 × 1 H, H-2, H-8), 7.32 (s, 2 H, N*H*_2_), 5.91 (d, *J* = 6.1 Hz, 1 H, H-1′), 4.79 (t, *J* = 5.6 Hz, 1 H, H-2′), 4.25–4.19 (m, 1 H, H-3′), 3.99 (td, *J* = 6.1, 3.3 Hz, 1 H, H-4′), 2.89 (dd, *J* = 13.7, 6.0 Hz, 1 H, H-5′a), 2.78 (dd, *J* = 13.8, 6.3 Hz, 1 H, H-5′b) ^13^C NMR (100 MHz, DMSO) *δ* (ppm) 156.1, 149.5 (2 C, C-4, C-5), 152.7 (2 C, C-2, C-8), 119.3 (1 C, C-6), 87.4, 85.5, 72.7, 71.9 (4 C, C-1′, C-2′, C-3′, C-4′), 26.6 (1 C, C-5′). MALDI-ToF MS: *m/z* calcd for C_10_H_14_N_5_O_3_S [M + H]^+^ 284.0811, found 284.0804.

### Detection of H_2_S release of 5′-dithioacetyl nucleosides

The H_2_S-donating ability of compound **22**, **24**, **27**, **29** and **30** (0.5 mg dissolved in 0.1 mL of DMSO) was detected in Dulbecco’s modified Eagle’s medium (DMEM) (4.9 mL) supplemented with 10% fetal bovine serum and 1% penicillin-streptomycin. The final concentrations were 0.31 mM for compound **22**, 0.29 mM for compound **24**, 0.28 mM for compound **27**, 0.32 mM for compound **29** and 0.32 mM for compound **30**, respectively. The released H_2_S was detected by an amperometric, H_2_S specific sensor (ISO-H2S-100, World Precision Instruments, Saratosa, FL, USA) connected to a WPI TBR 1025 One-Channel Free Radical Analyzer. The sensor was set to 10 nA range and poise voltage to + 150 mV. Before the measurements the sensor was polarized in phosphate buffered saline (PBS) for 12 h^19^. The H_2_S-release of NaHS was detected in distilled water (0.1 mg in 5 mL of H_2_O; 0.36 mM) and the H_2_S-release of GYY4137 was measured in DMEM (0.5 mg of GYY4137 in 5 mL of medium; 0.27 mM).

### Antioxidant assays

#### Determination of antioxidant activity by 2,2-diphenyl-1-picrylhydrazyl (DPPH) assay

DPPH is a stable free radical with a deep violet color that changes to yellow when neutralized by an electron or hydrogen radical, indicating the antioxidant activity of the sample being tested. The DPPH radical scavenging abilities were investigated following the method outlined by Shimamura et al., with minor adjustments^53^. In this study, a cardiovascular tissue homogenate was used to induce H_2_S release from the dithioacetate derivatives **22** and **24**, as well as GYY4137. A mixed solution of 1.2 mL of methanol and 800 µL of 0.1 M Tris-HCl buffer (pH 7.4) served as the blank. 1 mL DPPH solution was added to a mixture of methanol and 0.1 M Tris-HCl buffer (200:800 µL). After mixing, the absorbance was recorded at 517 nm on a UV-1600 PC VWR spectrophotometer (VWR, China). To avoid any potential interference from the heart extract in the assay, we measured the absorbance after adding different volumes of the extract into the mixture. These measurements were taken both immediately and after 30 min of incubation. Ultimately, the highest volume tested, 20 µL, which showed no activity at either time point, was selected for further tests.

The DPPH antioxidant assay was performed according to the following procedures. 1 mL of DPPH solution was added to a mixture of 200 µL of the methanolic sample solution and 800 µL of 0.1 M Tris-HCl buffer (pH = 7.4). For the groups containing the extract, the extract was introduced into a mixture of the sample solution in PBS buffer, 0.1 M Tris-HCl buffer, and DPPH (200:800:1000 µL). Considering that GYY4137 produces H_2_S upon hydrolysis, it was introduced last following the addition of the extract into a solution of PBS buffer, 0.1 M Tris-HCl buffer, and DPPH maintained at the same ratio. The same protocol was employed for NaHS without the addition of the extract. After mixing, the absorbance was promptly recorded, after which the solutions were incubated in the dark at room temperature for 30 min before the absorbance was measured again. The test samples were prepared and assayed at varying concentrations using a 2-fold serial dilution method, starting from a concentration of 2 mM. Each investigation was performed in triplicate, and the antioxidant activity was based on the absorbance recorded at 517 nm. Ascorbic acid (AA), GYY4137, and NaHS served as positive controls, while the unmodified nucleosides were used as reference compounds.

The inhibition ratio (IR) was calculated as follows:

$$\:Inhibition\:Ratio\:\left(\%\right)=\:\left(\frac{Ac-\:As}{Ac}\right)*100\:$$

where *Ac* is the absorbance when methanol is added instead of the sample and *As* is the absorbance when the testing sample solution is added.

The antioxidant capacity was expressed as the concentration needed to achieve a 50% inhibition of the free radical (IC_50_). The IC_50_ values were calculated by plotting the percent of inhibition as a function of sample concentration.

### ABTS decolorization assay

The ABTS decolorization assay was carried out as described by Szoke et al.^54^ Briefly, the method is based on the scavenging of (2,2′-azinobis-(3-ethylbenzothiazoline-6-sulfonic acid) ABTS^•+^ radical cation. The decolorization caused by the compound is compared to the decolorization induced by Trolox (6-hydroxy-2,5,7,8-tetramethylchroman-2-carboxylic acid), and expressed as Trolox equivalent antioxidant capacity (TEAC) value. TEAC values were expressed as Trolox equivalents calculated from (Ai − Af)antioxidant/(Ai − Af) Trolox at 0.1 mM concentrations. Briefly, a total of 10 µL sample, 20 µL of Myoglobin Working Solution, and 150 µL of ABTS Substrate Working Solution were mixed and incubated for 5 min at room temperature. At the end of incubation 100 mL of Stop Solution was added and endpoint absorbance at 405 nm was read. All experiments were performed in triplicates and repeated three times.

### MTT assay

H9c2 viability was measured by MTT assay (3-(4,5-dimethylthiazol-2-yl)-2,5- (diphenyltetrazolium bromide)) on 96-well plates, according to the method described in the literature^55^. Briefly, H9c2 cells were plated at density of (3 × 10^3^ cells/well) and grown overnight in a humidified incubator with 5% CO_2_ at 37 °C for 24 h. For the determination of IC_50_ values, cells were treated with the following conditions: fresh culture medium alone (control), fresh culture medium with compound **22**, **24**, **32** and **34** at concentrations ranging from 1 µM to 1000 µM for 24 h. After that, 20 µL of 5 mg/mL MTT solution was added to each well. The plate was again incubated for 3 h at 37 °C. During incubation, a conversion of the water-soluble yellow dye MTT into an insoluble purple formazan by the action of mitochondrial reductase occurred. Formazan crystals were solubilized by adding 200 µL of isopropanol to each well. The concentrations were determined by optical density at 570 nm. The background absorbance was also recorded at 690 nm, which was subtracted. Cell viability was expressed as a percent of the cell viability of the untreated control cells. After 24 h, cells were treated with compound **22**, **24**, **32** and **34**. On the next day, cell viability was measured by MTT assay as described previously^55^.

### Antiviral assay

#### Anti-SARS-CoV-2 activity and cytotoxicity determination in Calu-3 cells

The anti-SARS-CoV-2 activity was measured by determining the extent to which the compounds inhibited virus-induced cytopathic effect (CPE) in Calu-3 cells (ATCC). Briefly, 40,000 Calu-3 cells in a 96-well plate in DMEM medium with 2% FBS, 100 U of penicillin/mL and 100 µg of streptomycin/mL (all Merck) were infected with SARS-CoV-2 (strain hCoV-19/Czech Republic/NRL_6632_2/2020) at multiplicity of infection 0.3. After 2 h, the medium was removed, cells were washed two times with 1X DMEM and two-fold fold serial dilutions of compounds from 1000 µM were added in triplicate. Following two days incubation at 37 °C in 5% CO_2_ the cell viability was determined by adding CellTiter-Glo luminescent cell reagent (Promega) using Tempest liquid dispenser (Formulatrix). The luminescent signal produced by ATP-dependent oxidation of luciferin by viable cells was measured using EnVision plate reader (Perkin Elmer). Drug concentrations required to reduce viral cytopathic effect by 50% (EC50) were calculated using nonlinear regression from plots of percentage cell viability versus log_10_ drug concentration using GraphPad Prism v.9.5.1 (GraphPad Software).

Cytotoxicity was evaluated by incubating the same two-fold serial dilutions of each compound with Calu-3 cells as above in the absence of the virus. Following two days incubation at 37 °C in 5% CO_2_, the cell viability was determined as above. The compound concentrations resulting in 50% reduction of cell viability (CC_50_) were calculated from plots of percentage of luminescence versus log_10_ drug concentration as above. Remdesivir was used as control in both assays.

### Virus yield reduction assay

The reduction in virus titer was assessed in medium after 2 days infection of Calu-3 in the Vero E6 cells by plaque assay. Briefly, 50 µl of medium from Calu-3 cells infected in the presence of the highest compound concentration was mixed with DMEM medium followed by 10-fold dilution, mixed with Vero E6 cells, added to 24-well plate and incubated for 4 h at 37 °C in 5% CO_2_. Next, the mixture was overlaid with 500 µl of 3% carboxymethyl cellulose (Merck) and incubated for 5 days at 37 °C in 5% CO_2_. Remdesivir was included as control. After incubation, the cells were washed once with 1 × PBS, stained with naphthalene black (Merck), washed with water and air-dried. Plaques were counted and expressed as log_10_ plaque forming units (PFU) per mL.

### In vivo studies

The cardiac effects of adenosine, compound **22**, **24** and **34** were assessed with electrocardiography (ECG) and echocardiography on anaesthetized Sprague-Dawley rats (weighing 600–800 g, Charles River Laboratories) as described previously^56^. The animals received humane care, and all experimental procedures were carried out in accordance with the “Principles of Laboratory Animal Care” by EU Directive 2010/63/EU. All experimental protocols were approved by the local Ethics Committee of University of Debrecen (25/2023/DEMÁB). Animal experiments are reported in compliance with the ARRIVE guidelines^57^. Anesthesia was carried out using ketamine/xylazine mixture (100/10 mg/kg, i.m.). The chest was shaved and the animal was placed onto a heated pad (VisualSonics SR200, VisualSonics, Amsterdam, The Netherlands). The lateral tail vein was cannulated using a heparinized 26G i.v. cannula (BD Neoflon, BD Medical, NJ, USA). B-mode and M-mode echocardiography was carried out before and 30 s after the intravenous administration of the compounds. A high-resolution ultrasound machine (Vevo 3100 Imaging Station, VisualSonics, Amsterdam, The Netherlands) equipped with a 21 Hz transducer (MX201) was used for assessing cardiac parameters. Traces were obtained from the parasternal long axis view (PLAX). A B-mode video and an M-mode trace was recorded, and the following parameters were then offline analysed: left ventricle (LV) end-systolic and end-diastolic volume (LVESV, LVEDV, respectively) fractional shortening (FS) and ejection fraction (EF) and stroke volume (SV).

A 3-channel ECG was continuously recorded during the experiments (Vevo monitor ECG, VisualSonics, Amsterdam, The Netherlands). On the recorded ECG data, RR, PR and QT intervals, and heart rate (HR) were determined before and 30 s after the administration of the drugs, and 6 consecutive cardiac cycles were averaged for each parameter.

Statistical analyses for the in vivo studies were carried out using the GraphPad Prism 9.0 software (GraphPad Software, San Diego, CA, USA). Data distribution was assessed by the Shapiro-Wilk normality test, and paired t-test were used to determine the differences between the baseline (drug free) and endpoint (treated) parameters in each group. A multiple comparison was also carried out between the treatment groups by using one-way ANOVA with Tukey multiple comparison test. Data was shown as mean ± SD, and differences with *p* < 0.05 were considered significant.

## Electronic supplementary material

Below is the link to the electronic supplementary material.

Supplementary Material 1

## Data Availability

The datasets used and/or analysed during the current study available from the corresponding author (A.B.) on reasonable request.

## Abbreviations

A: Adenine
ABTS: 2,2′-azinobis-(3-ethylbenzothiazoline-6-sulfonic acid)
Ac: Acetyl
Bu_4_NBr: Tetrabutyl ammonium bromide
C: cytosine
DIAD: Diisopropyl azodicarboxylate
DMF: Dimethylformamide
DPPH: 2,2-Diphenyl-1-picrylhydrazyl
G: Guanine
HFIP: Hexafluoro isopropanol
HSAc: Thioacetic acid
MeOH: Methanol
MTT: 3-(4,5-dimethylthiazol-2-yl)-2,5- (diphenyltetrazolium bromide)
NaOMe: Sodium methylate
PPh_3_: Triphenyl phosphine
TFA: Trifluoroacetic acid
THF: Tetrahydrofuran
TLC: Thin layer chromatography
Trt: Triphenylmethyl
TrtCl: Triphenylmethyl chlorid
Ts: *p*-toluenesulfonyl
TsCl: *p*-toluenesulfonyl chloride
TsOH: *p*-toluenesulfonic acid
U: Uridine
