# Supplementary material for: Synthesis, H2S releasing properties, antiviral and antioxidant activities and acute cardiac effects of nucleoside 5′-dithioacetates
Source: Sci Rep. 2025 Jan 22;15:2876. doi: 10.1038/s41598-025-85351-1 (PMC11754443; doi:10.1038/s41598-025-85351-1)

Synthesis, H_2_S releasing properties, antiviral and antioxidant activities and acute cardiac effects of nucleoside 5’-dithioacetates

Miklós Bege,^1,2,3^* Miklós Lovas,^1^ Dániel Priksz,^4^ Brigitta Bernát,^4^ Ilona Bereczki,^1,5,6^ Rasha Ghanem Kattoub,^1,7^ Richárd Kajtár,^7,8^ Simon Eskeif, ^7,8^ Levente Novák,^9^ Jan Hodek,^10^ Jan Weber,^10^ Pál Herczegh,^1^ István Lekli,^8^ Anikó Borbás^1,2,6^*

^1^Department of Pharmaceutical Chemistry, Faculty of Pharmacy, University of Debrecen, Egyetem tér 1, 4032 Debrecen, Hungary; ^2^HUN-REN-UD Molecular Recognition and Interaction Research Group, University of Debrecen, Egyetem tér 1, 4032 Debrecen, Hungary; ^3^Institute of Healthcare Industry, University of Debrecen, 4032, Debrecen, Nagyerdei krt. 98, Hungary; ^4^Department of Pharmacology and Pharmacotherapy, Faculty of Medicine, University of Debrecen, Nagyerdei krt. 98, 4032 Debrecen, Hungary; ^5^HUN-REN-UD Pharmamodul Research Group, University of Debrecen, Nagyerdei krt. 98, 4032 Debrecen, Hungary; ^6^National Laboratory of Virology, University of Pécs, Ifjúság útja 20, H-7624 Pécs, Hungary; ^7^Doctoral School of Pharmaceutical Sciences, Faculty of Pharmacy, University of Debrecen, Nagyerdei krt. 98., 4032 Debrecen, Hungary; ^8^Department of Pharmacodynamics, Faculty of Pharmacy, University of Debrecen, Debrecen, Hungary, ^9^Department of Physical Chemistry, Faculty of Science and Technology, University of Debrecen, Debrecen, Hungary; ^10^Institute of Organic Chemistry and Biochemistry of the Czech Academy of Science, Prague, 166 10, Czech Republic

Supporting Information

Table of contents

[Antioxidant activity of heart extract in different volumes determined by DPPH assay S3](#_Toc184029620)

[The effect of the compounds on the viability of H9c2 cells measured by MTT assay S4](#_Toc184029621)

[Anti-SARS-CoV-2 activity and cytotoxicity determination in Calu-3 cells S5](#_Toc184029622)

[HPLC measurments S6](#_Toc184029623)

[NMR spectra of the compounds S9](#_Toc184029624)

# Antioxidant activity of heart extract in different volumes determined by DPPH assay


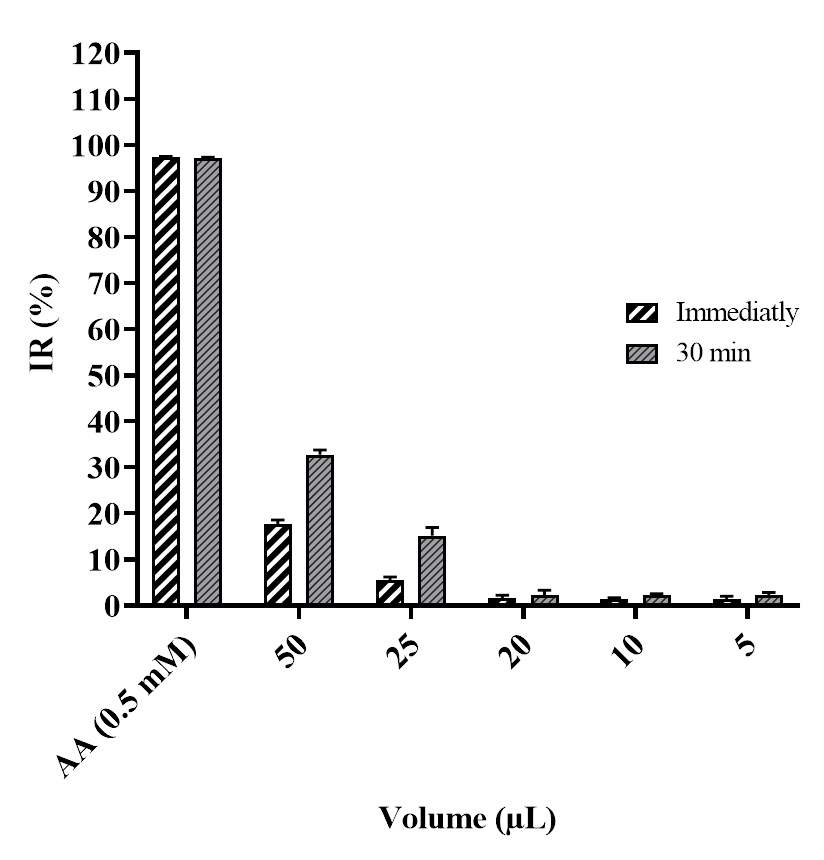


**Figure S1**: DPPH inhibition ratio upon the addition of different volumes of the extract compared to the positive control (Ascorbic acid (AA), IR = 97.25% and 97.13% immediately and after 30 min of incubation, respectively).

# The effect of the compounds on the viability of H9c2 cells measured by MTT assay


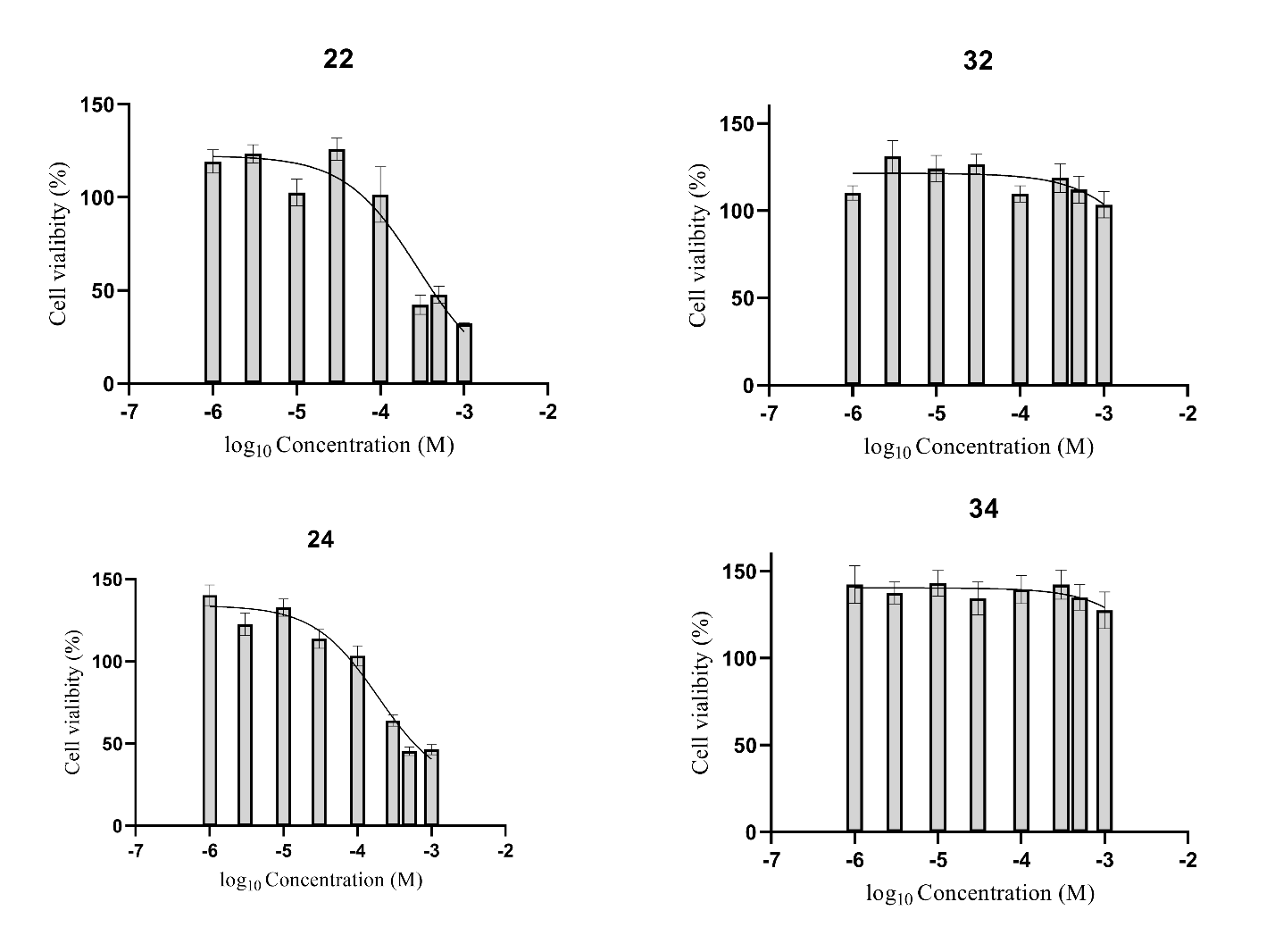


**Figure S2**. Cell viability assay using the MTT method. H9c2 cells were treated with different concentrations (1 µM, 3 µM, 10 µM, 30 µM, 100 µM, 300 µM, 500 µM 1000 µM) of nucleoside analogues or their metabolites and vehicle treated cells were used as control. The measurements were carried out in duplicates. Columns represent the mean ± SEM, (n = 8).

# Anti-SARS-CoV-2 activity and cytotoxicity determination in Calu-3 cells


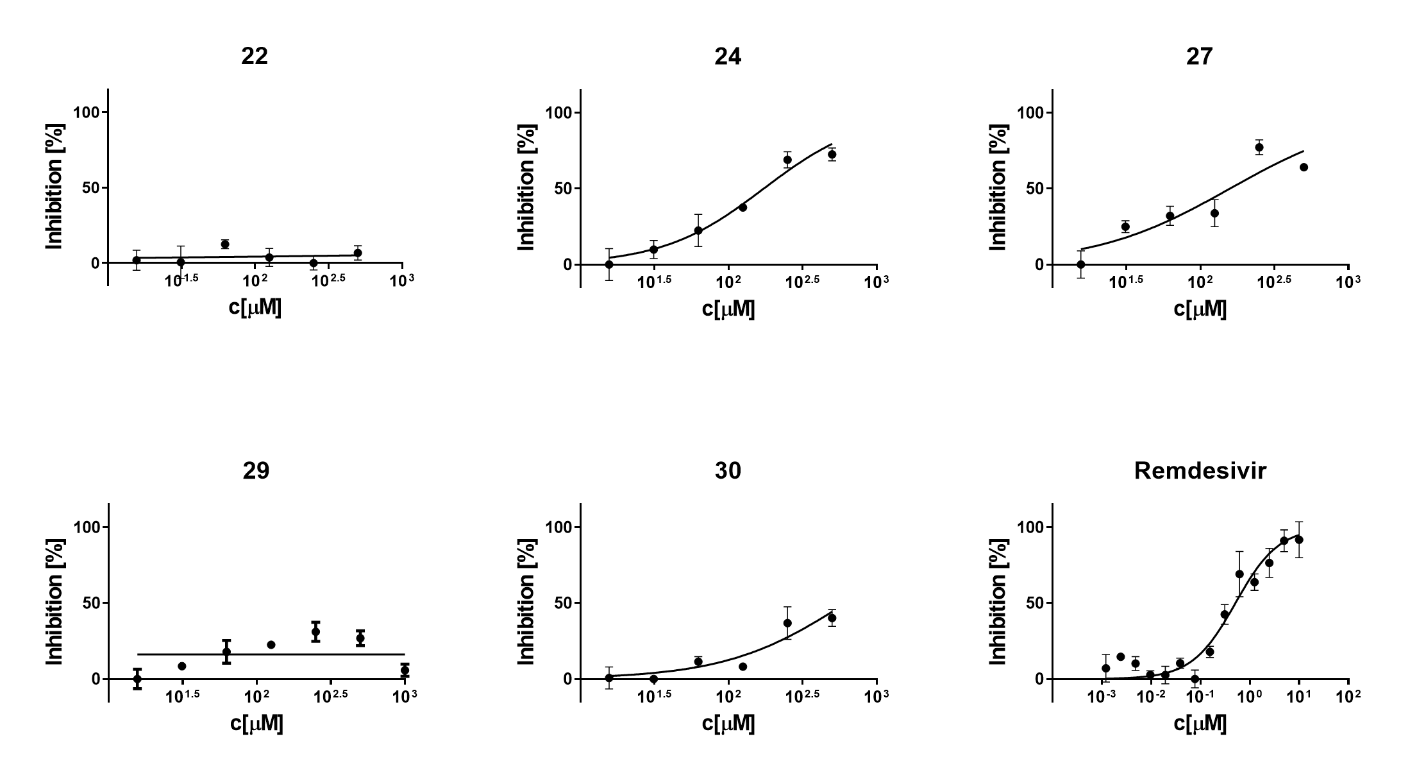


Figure S2. Inhibitory activity of compounds on virus-induced cytopathic effect (CPE) in Calu-3 cells (antiviral EC_50_ graphs)


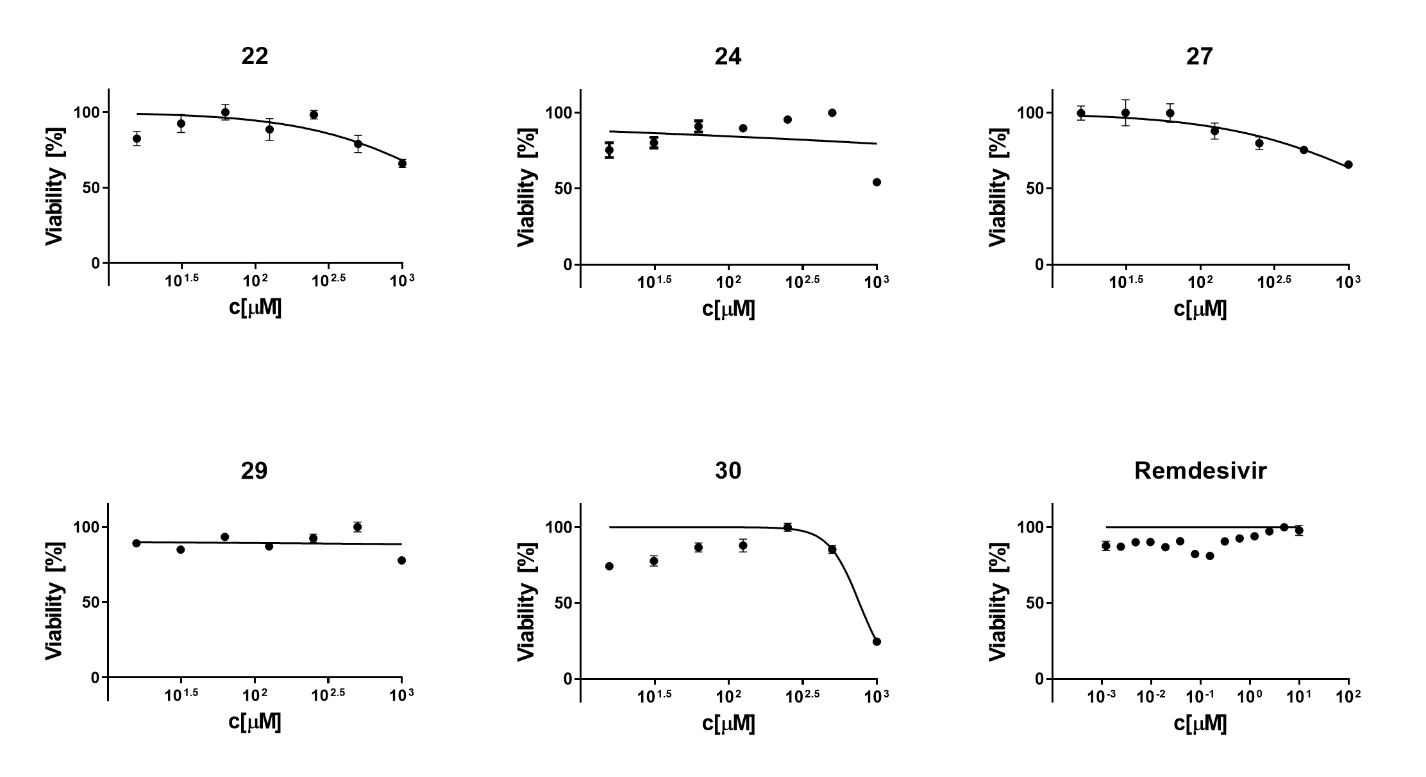


Figure S3. Effect of compounds on viability of Calu-3 cells(CC_50_ graphs)

# HPLC measurments

The purity of compounds **22, 32** and **34** was assessed via high-perfomance liquid chromatography (HPLC) measurements, using a LaChrom L-7200 device equipped with a X-Terra MS C18 3,5 µm 3,0×150 column, a L-7400 UV detector and L7100 pump. Samples were eluted with an gradient elution using eluent A (0.05% phosphoric acid in water) and eluent B (0.05% phosphoric acid in acetonitrile). The flow rate was set to 0.5 ml/min. The initial condition was 0% B eluent, followed by a linear gradient to 100% B eluent by 6 min; from 6 to 11.2 min 100% B eluent was retained and then from 11.2 to 13.5 min it was returned to the initial condition and retained to 3.5 min. The column temperature was kept at 30 ^o^C and the injection volume was 4.0 µL.

For RP-HPLC measurements of compound **22** a Waters 2695 Separations Module (Waters Corp., Milford, USA) was used. The separations were carried out on a VDSphere PUR 100 C18-M-SE, 5 μm, 150 x 4.6 mm column at an injection volume of 10 μL, using a flow rate of 1.0 mL/min with a Waters 2996 DAD as detector set at 254 nm. The following system was used for the elution: Solvent A: Water : MeCN 9 : 1 + 0.0025 V/V% TFA and Solvent B: MeCN. Gradient elution: from 20% of B to 80% from 0 to 40 min and 80% of B from 40-50 min.


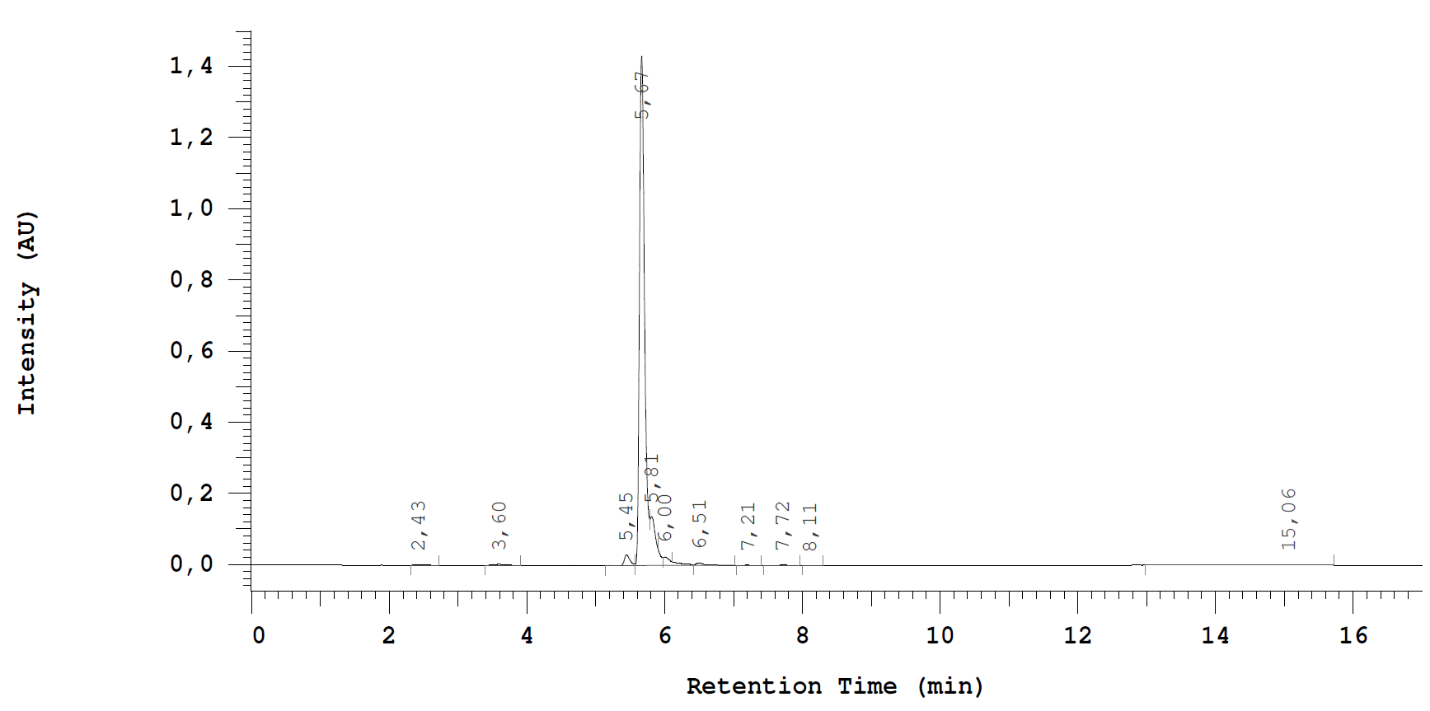


Figure S4. HPLC chromatogram of compound **32**

**
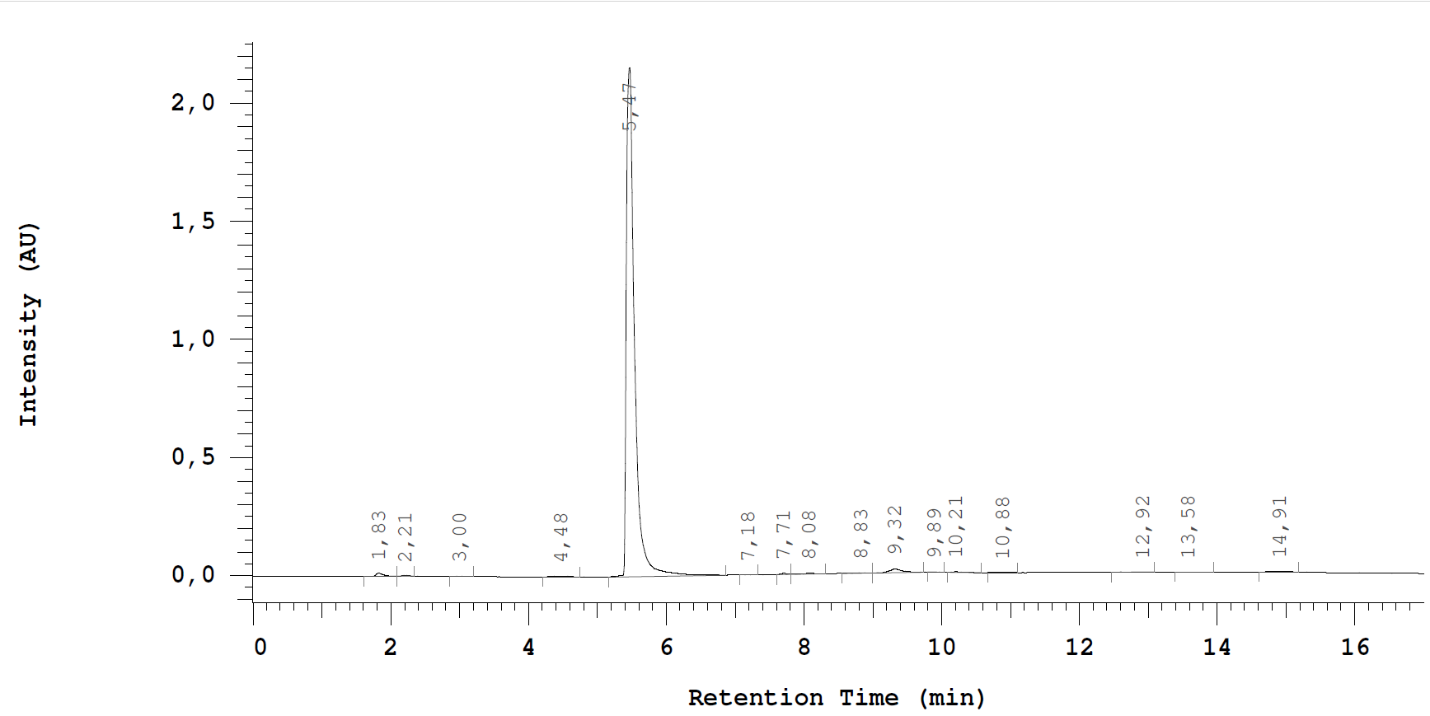
**

Figure S5. HPLC chromatogram of compound **34**

**
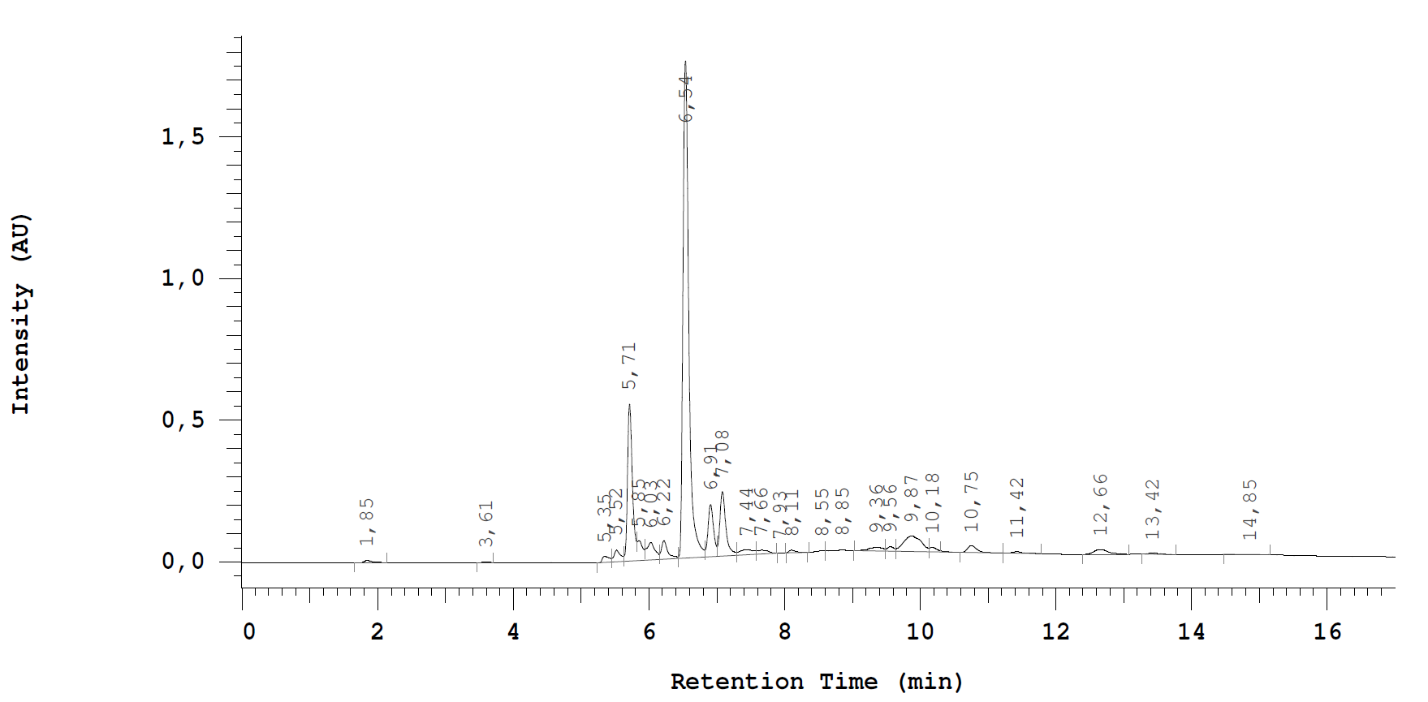
**

Figure S6. HPLC chromatogram of compound **22**

Figure S7: RP-HPLC chromatogram of compound **22**

# NMR spectra of the compounds

Compound **13**


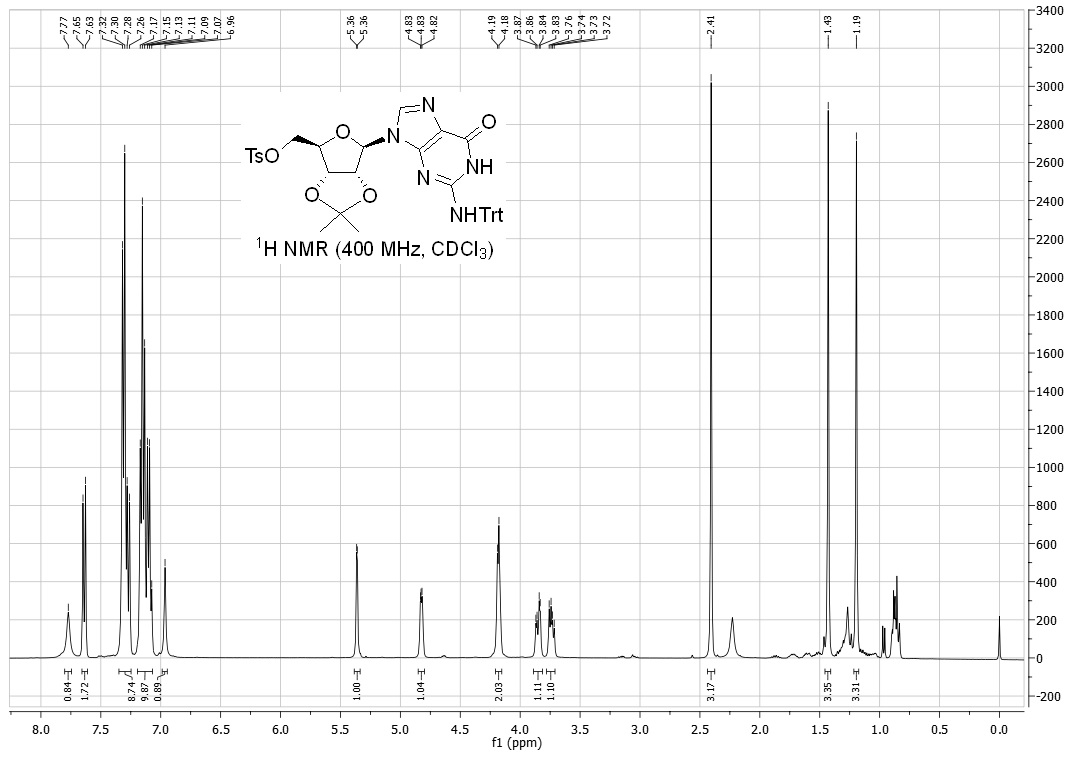


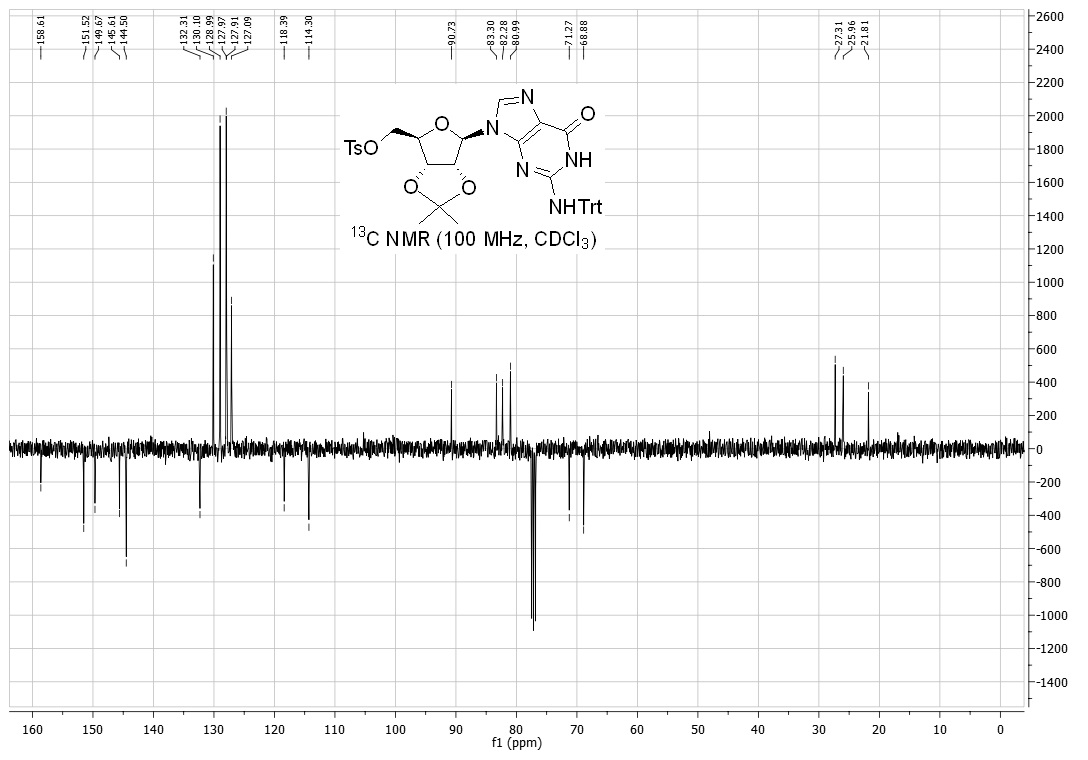


Compound **15**

**
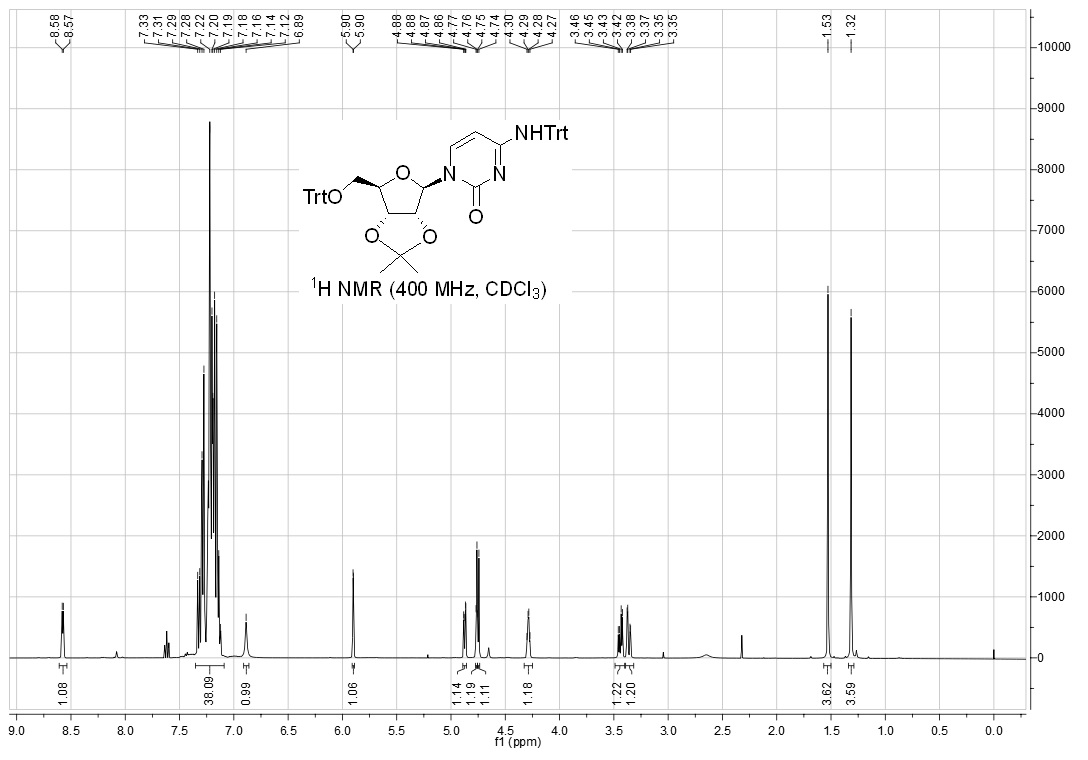
**


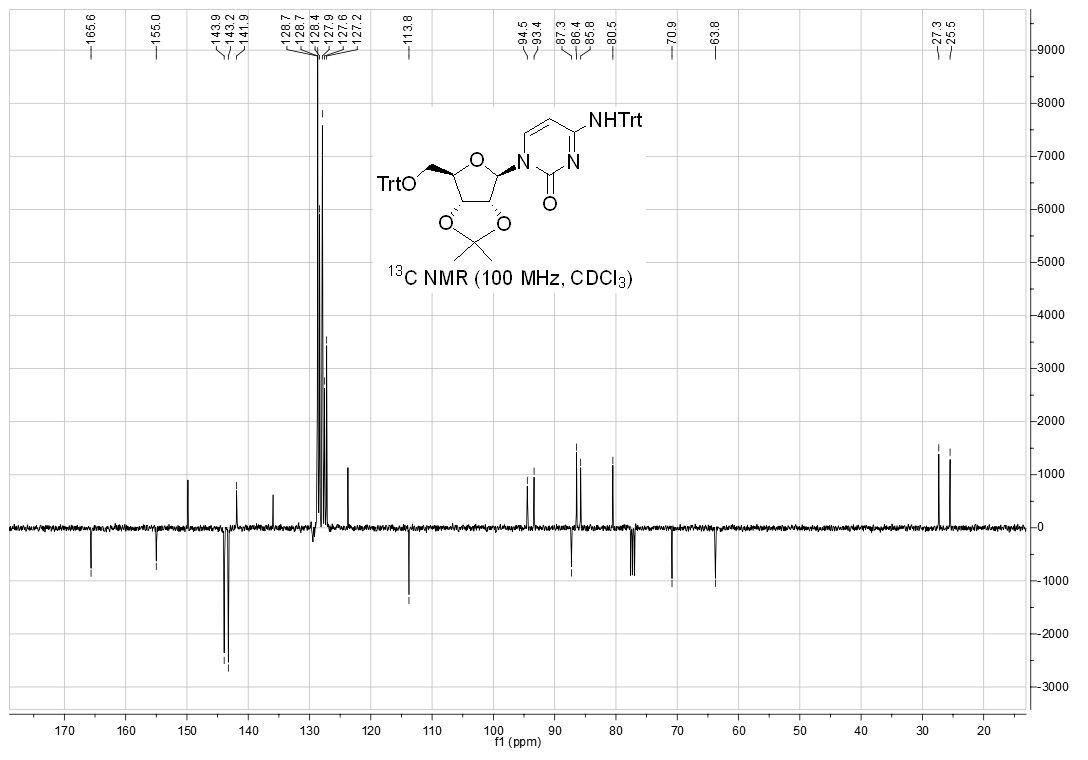


Compound **16**


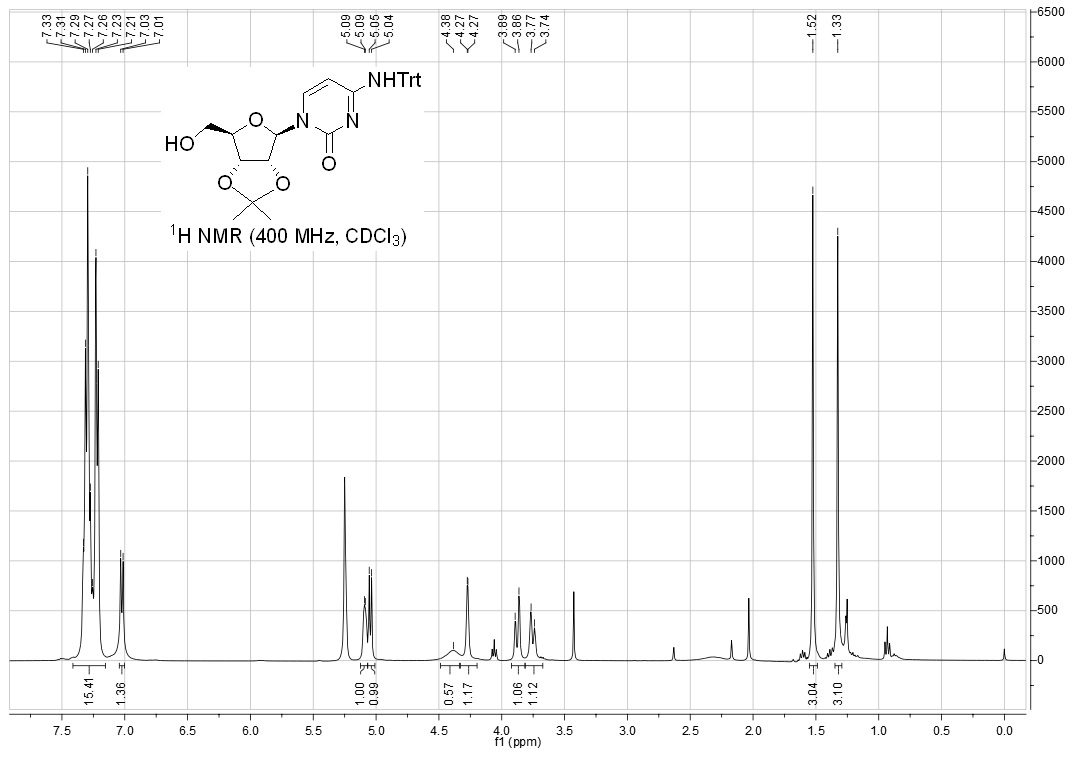


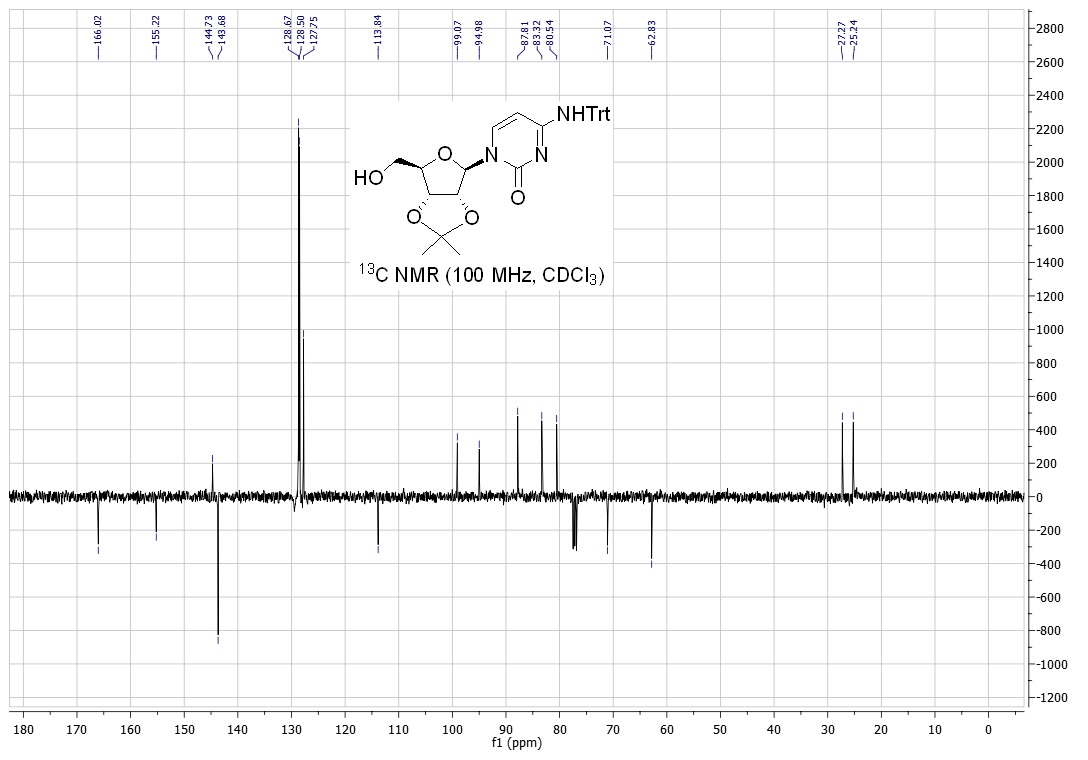


Compound **17**


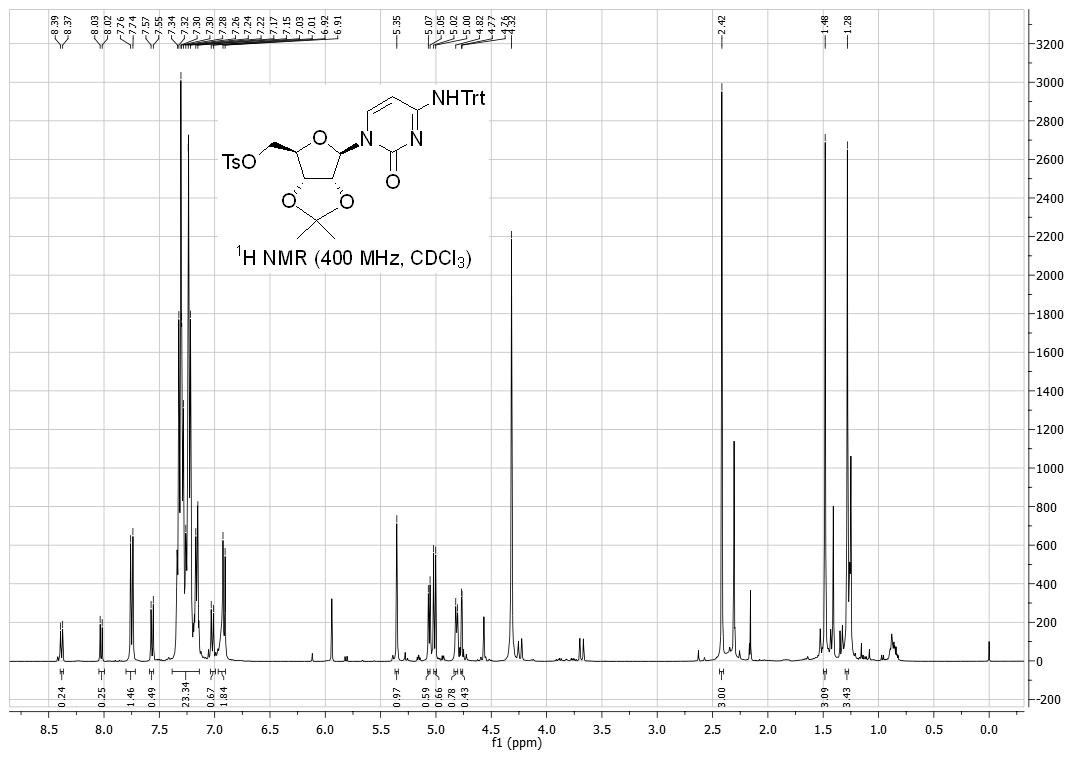


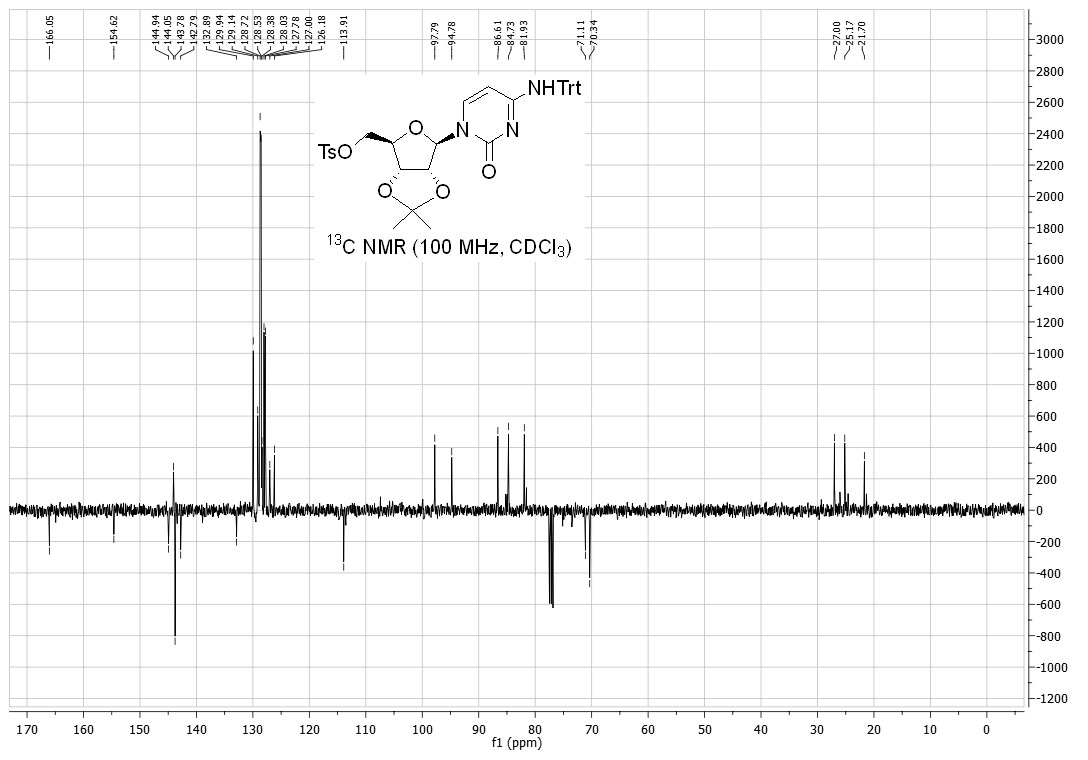


Compound **20**


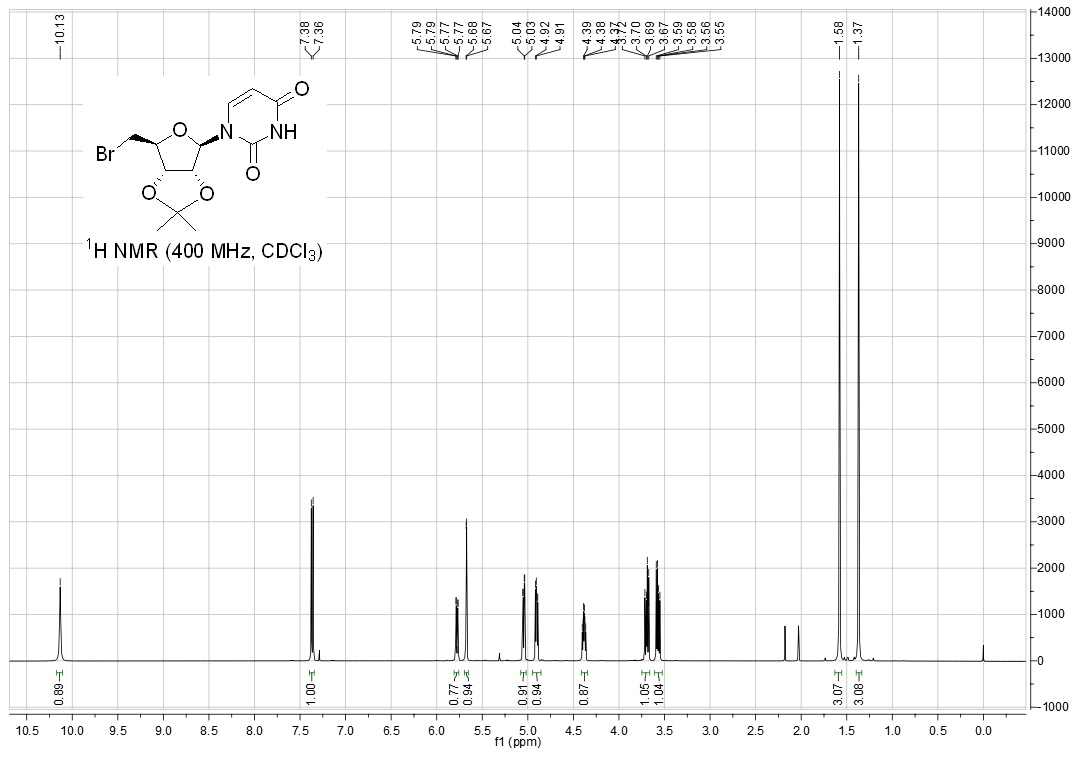


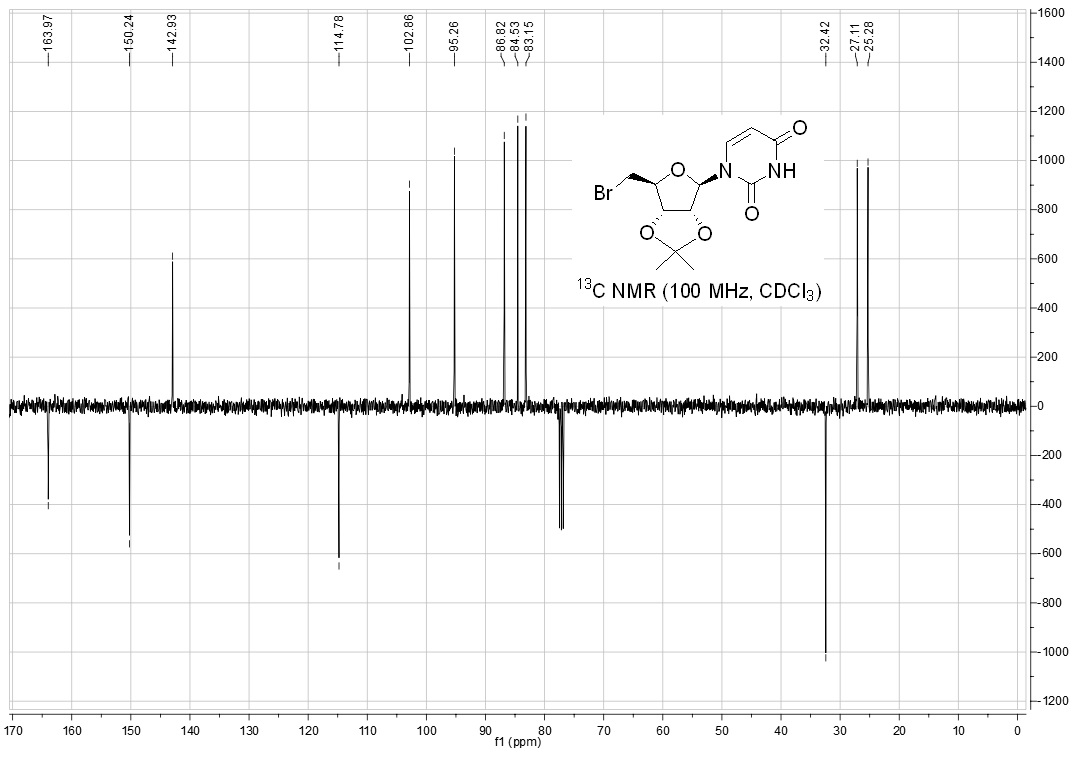


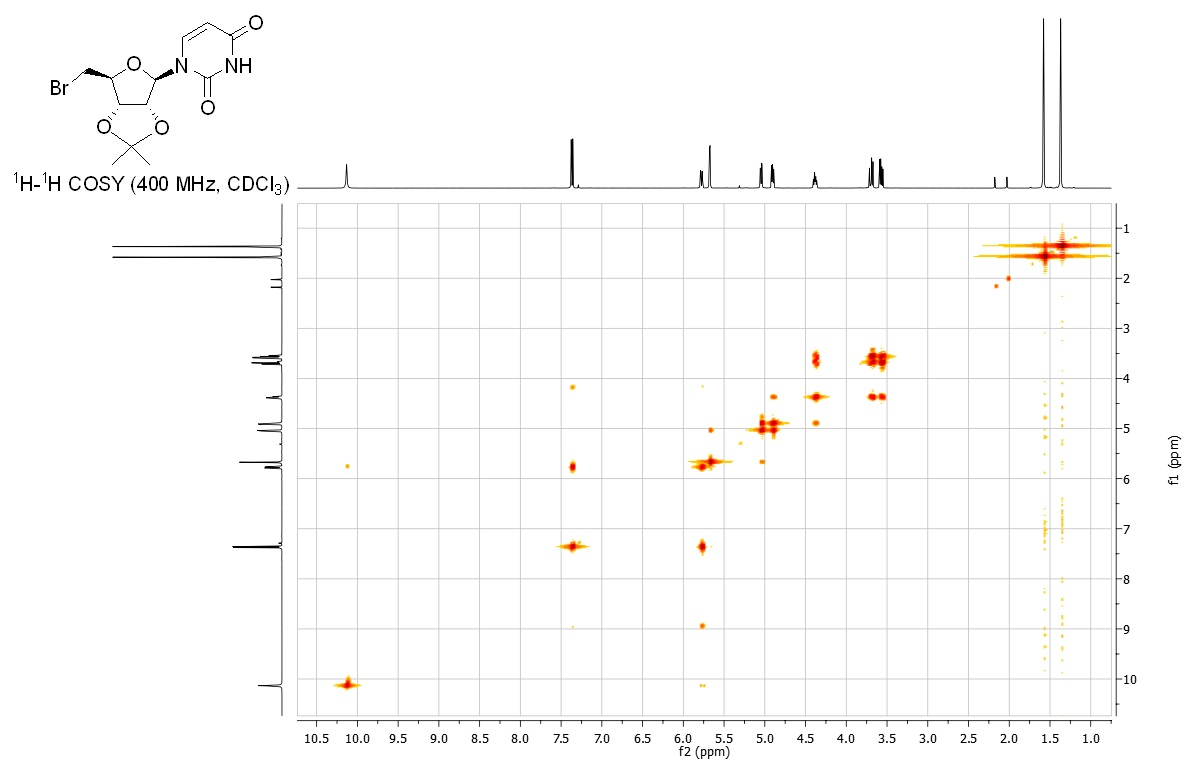


Compound **21**

**
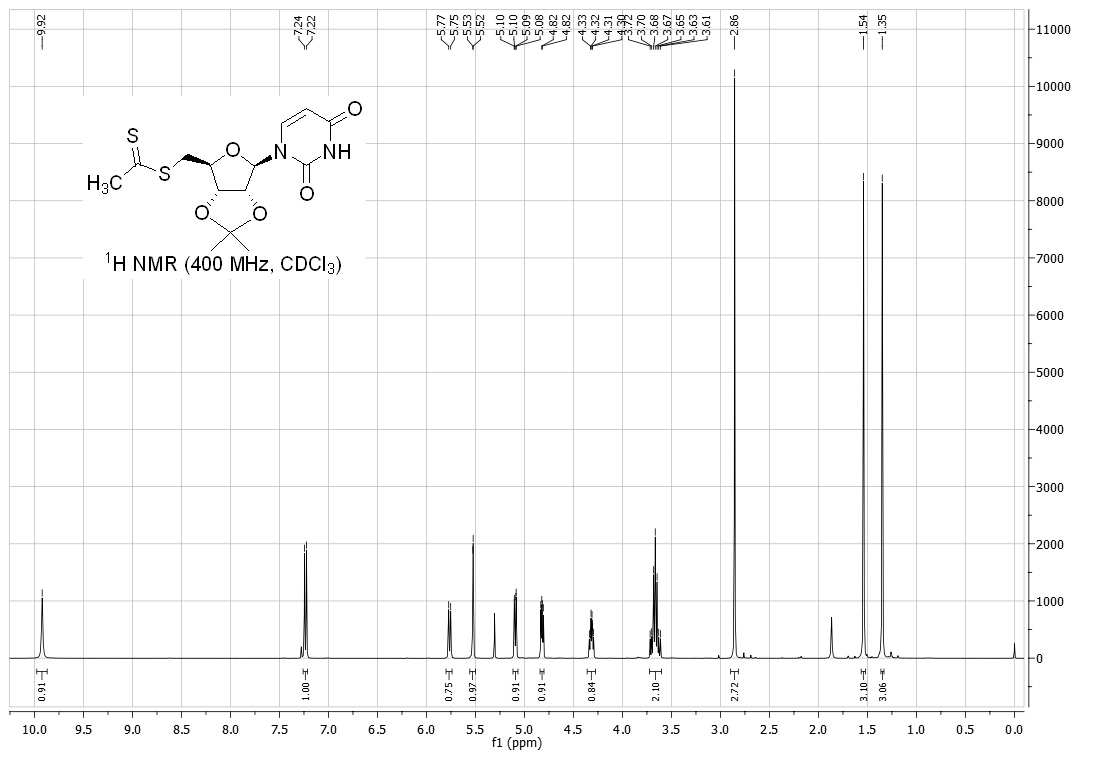
**


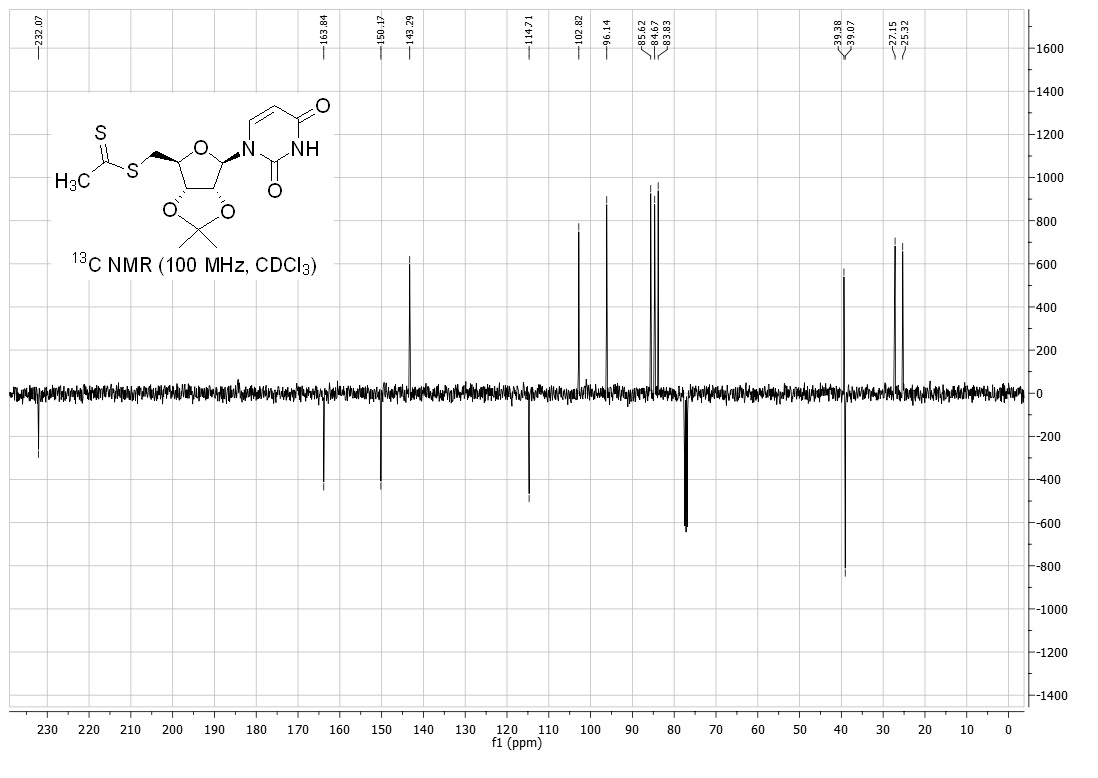


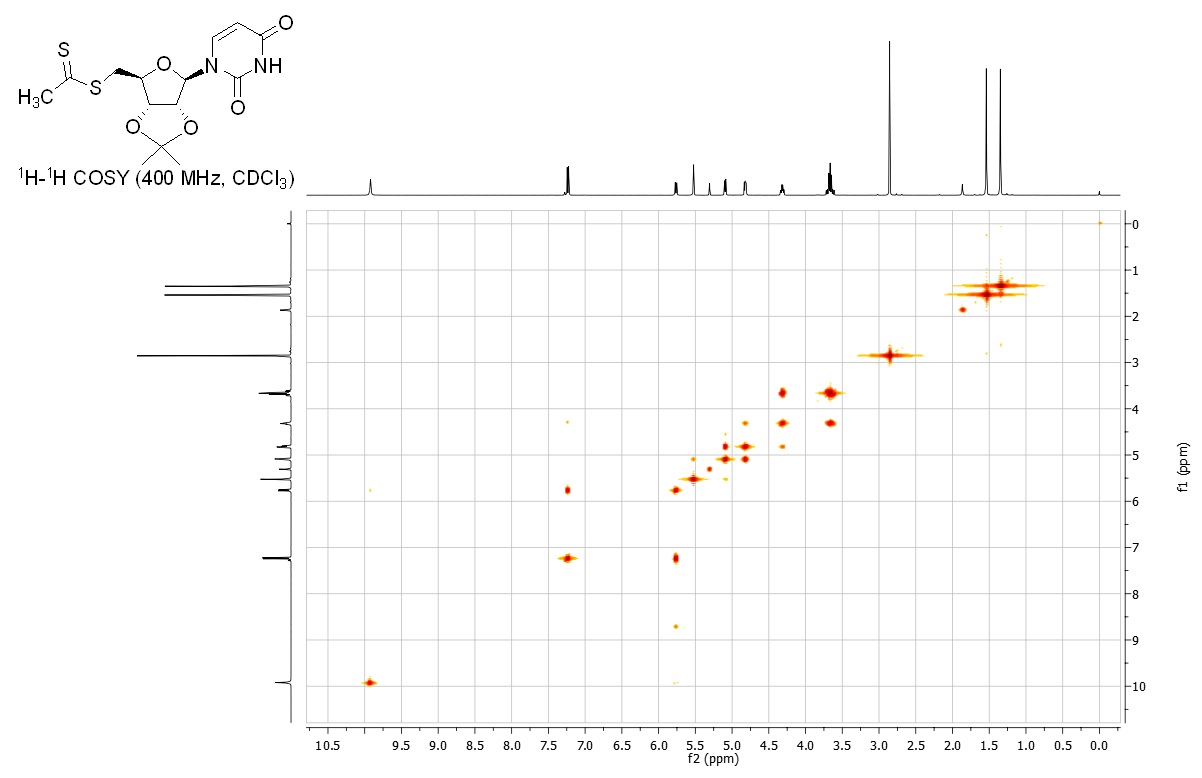


Compound **22**


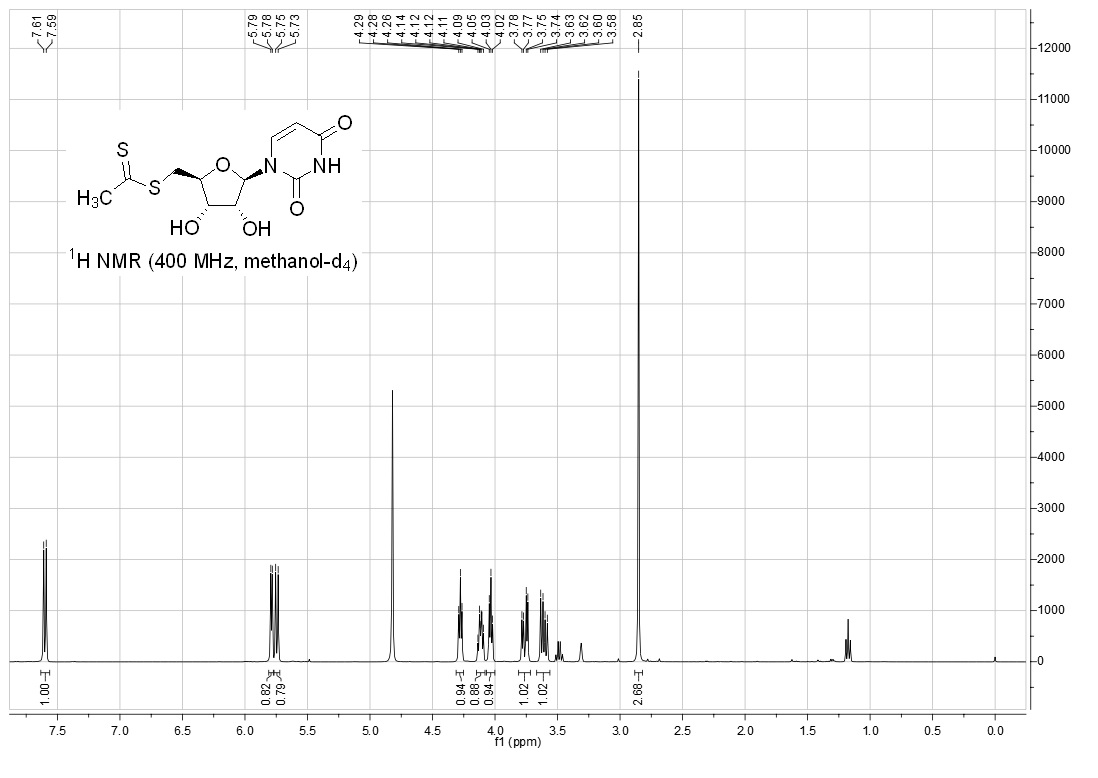


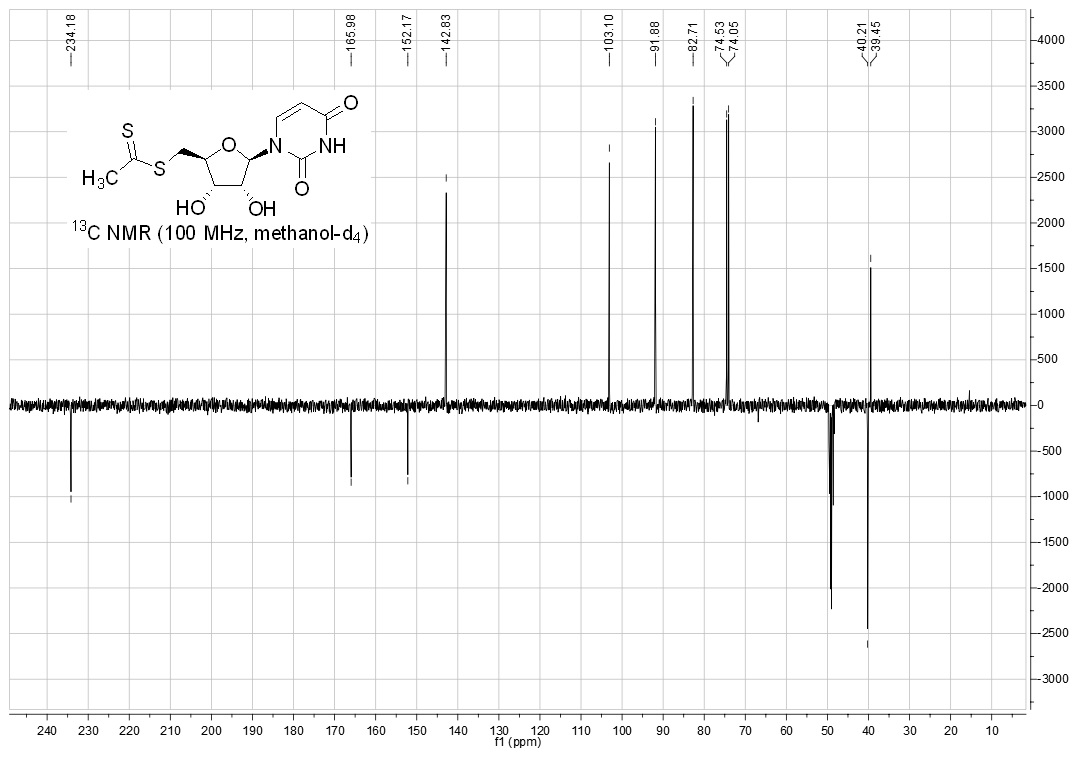


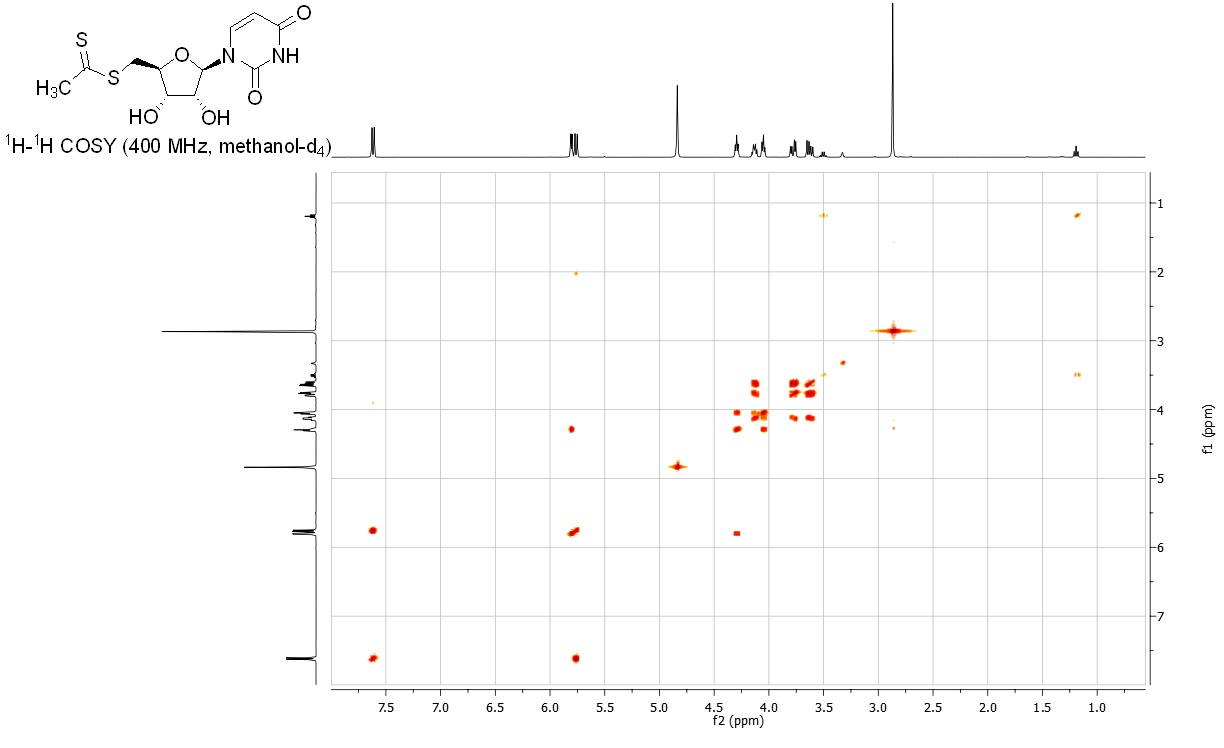


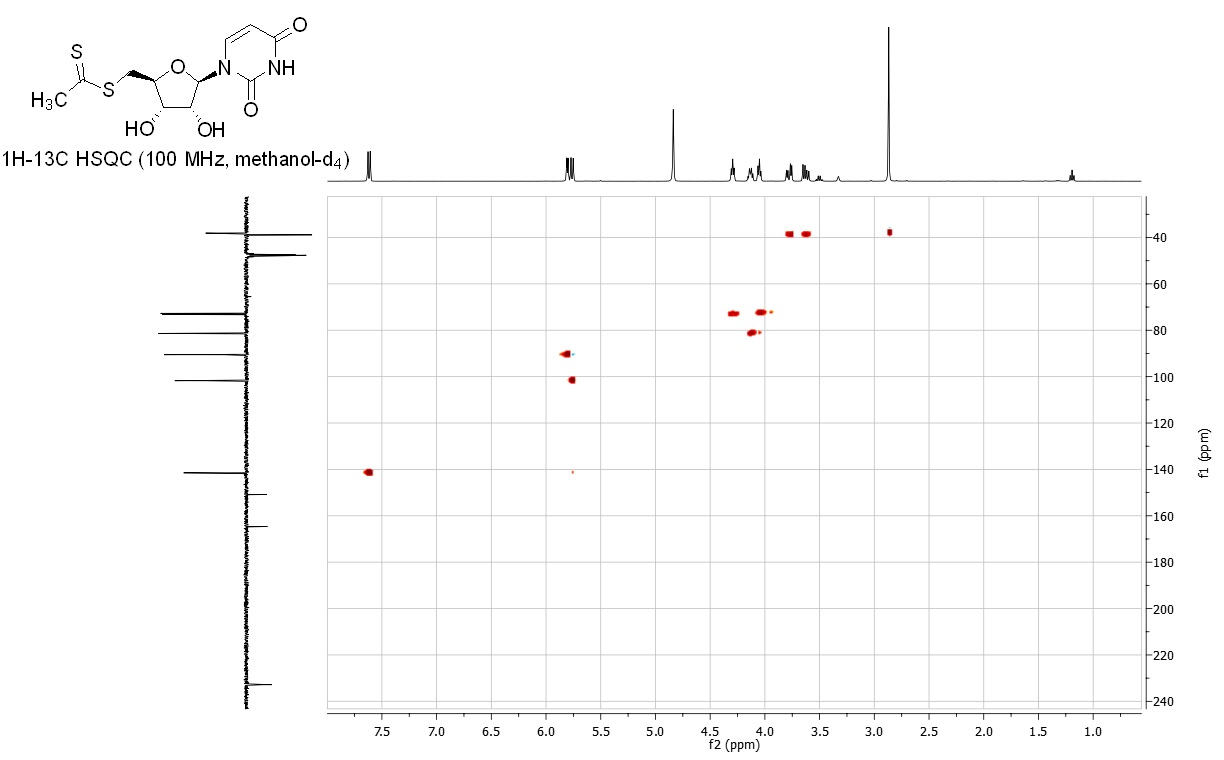


Compound **23**


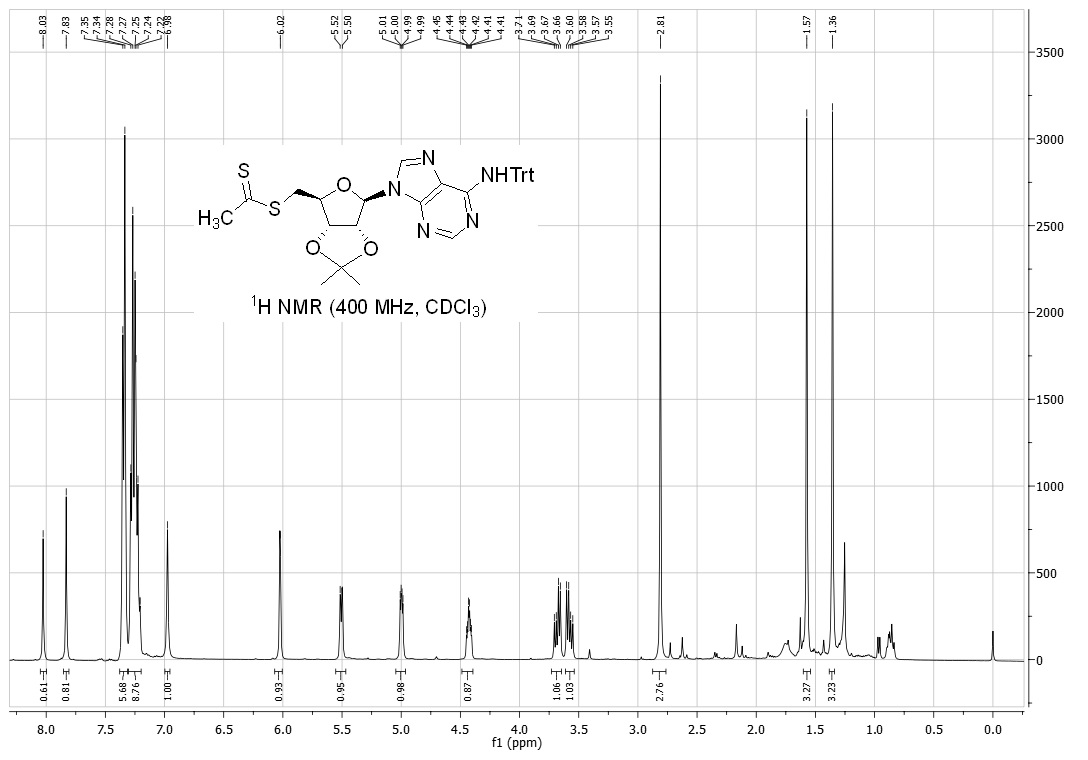


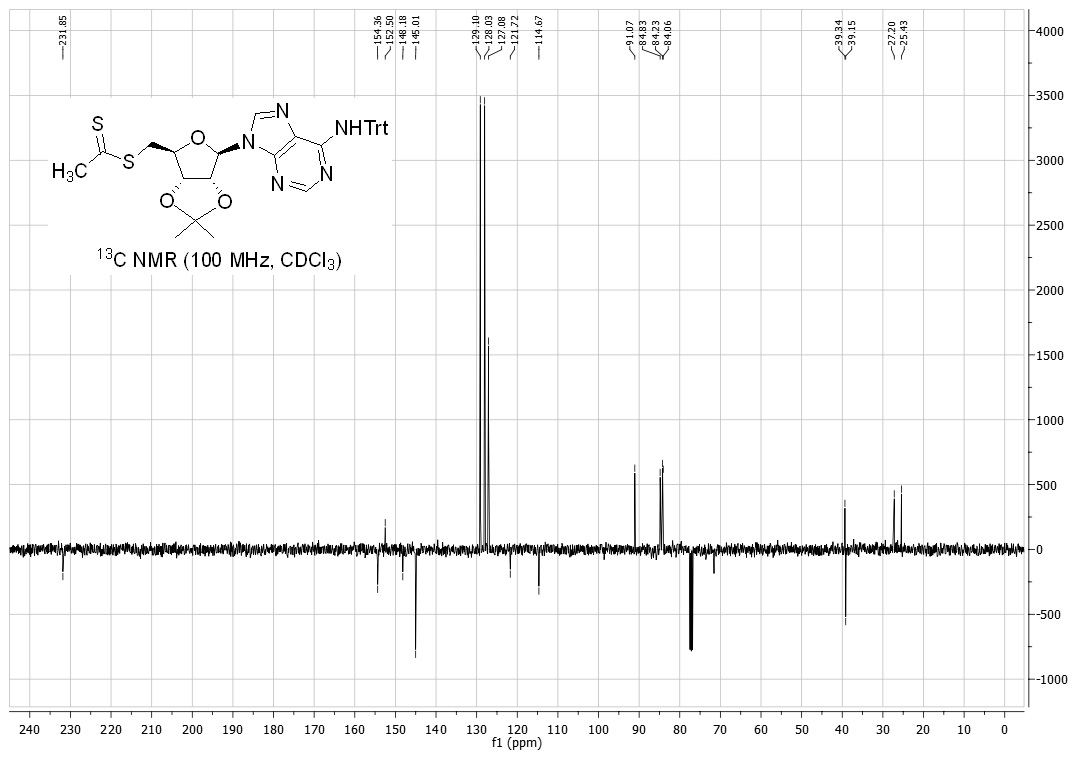


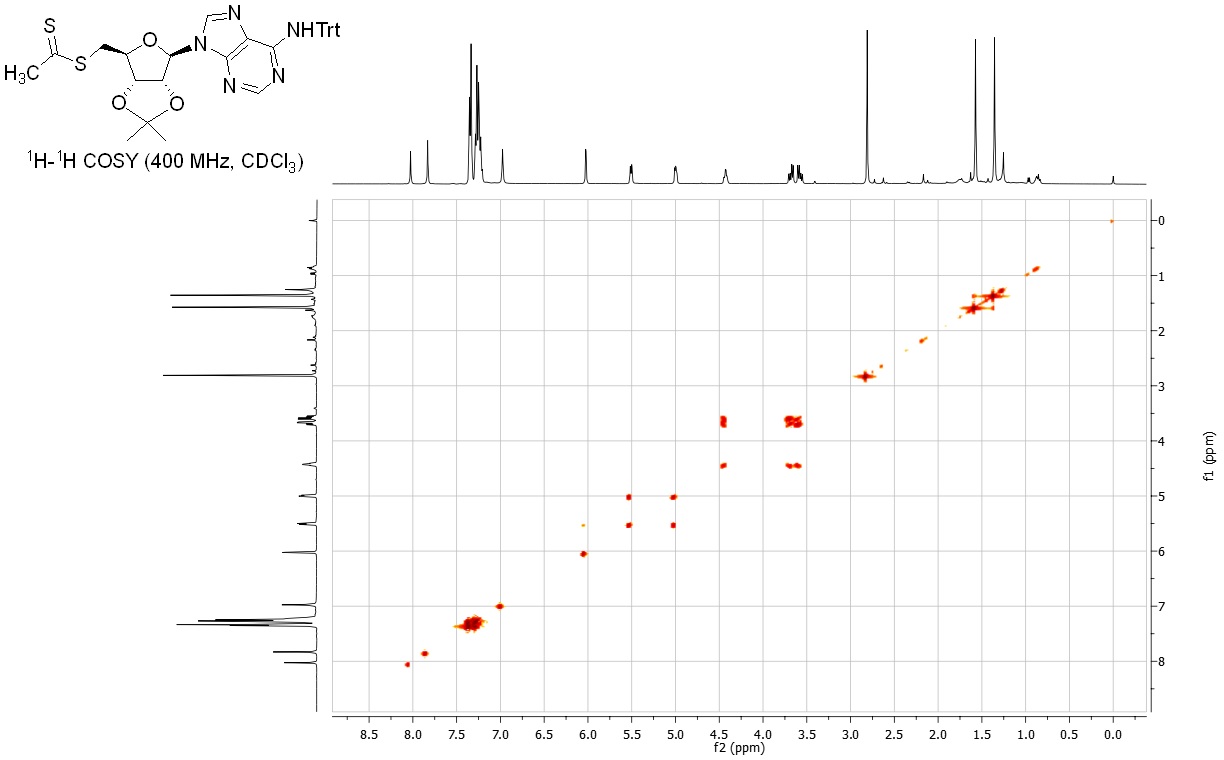


Compound **24**


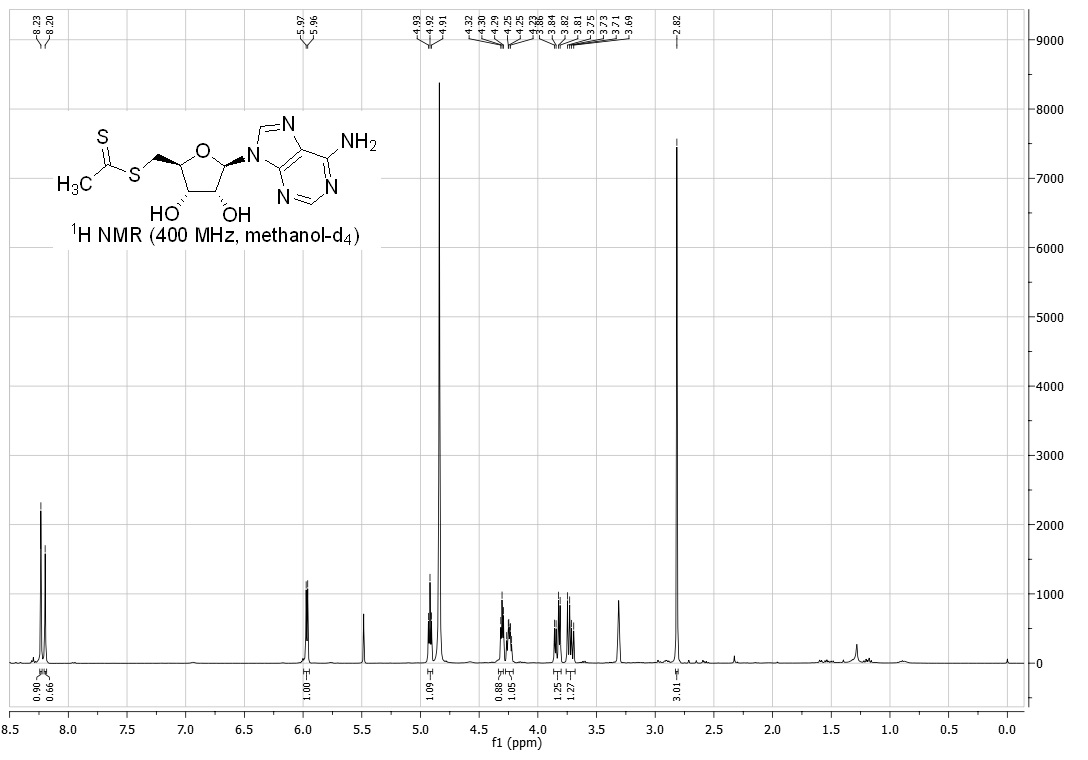


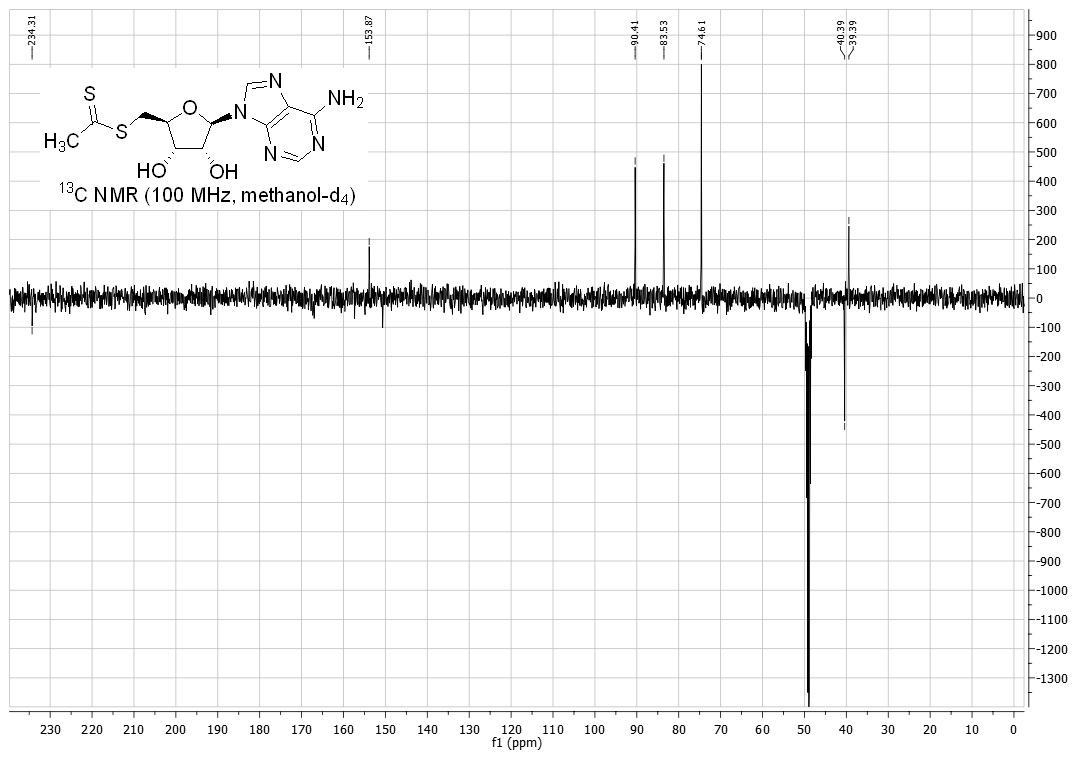


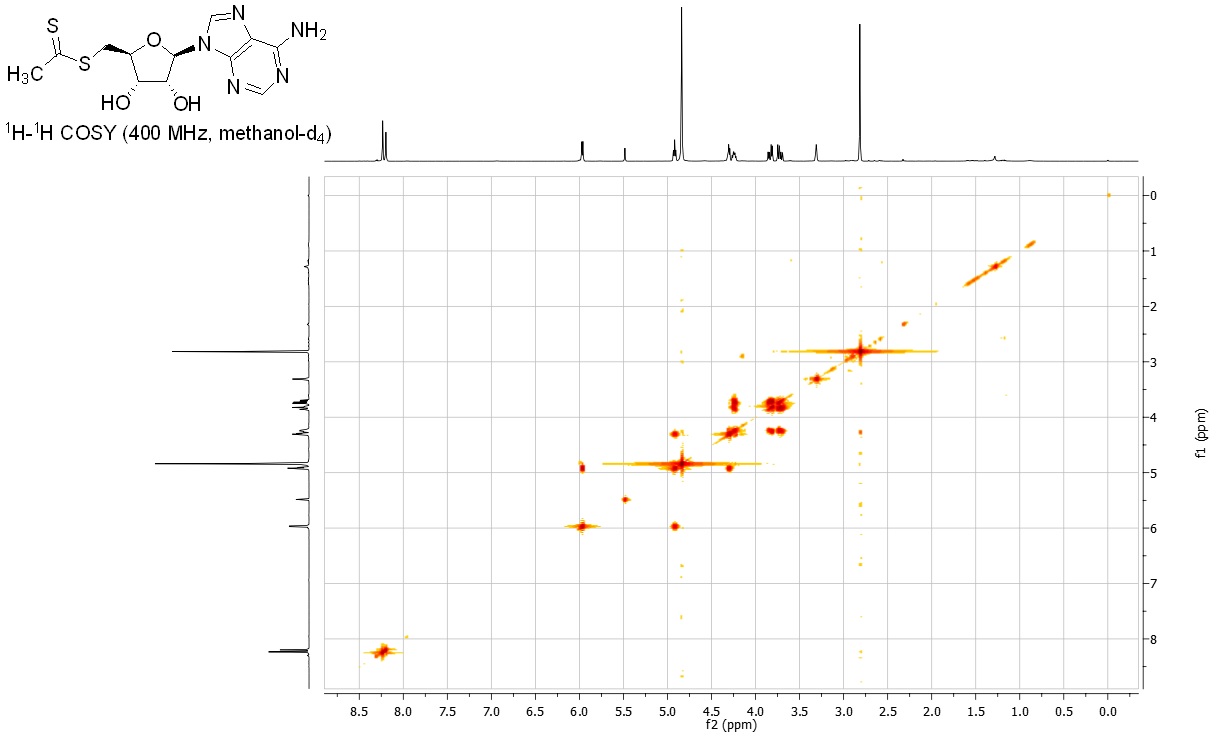


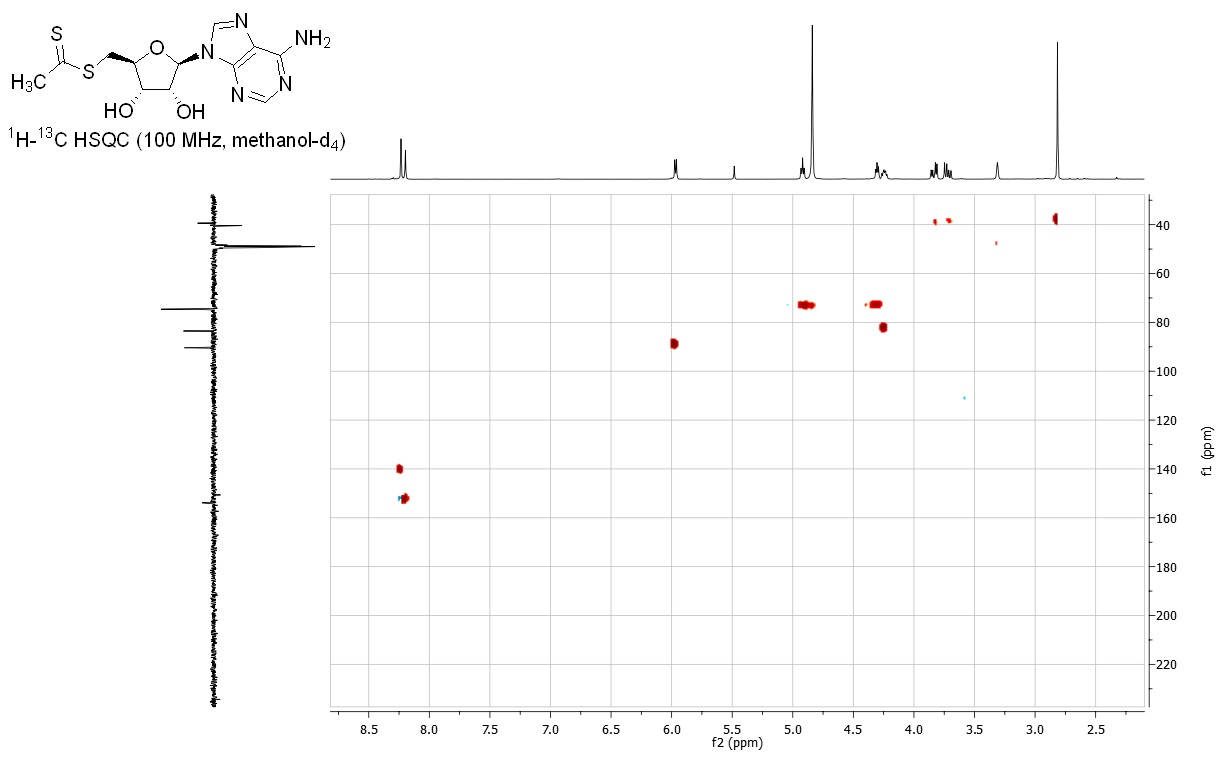


Compound **25**


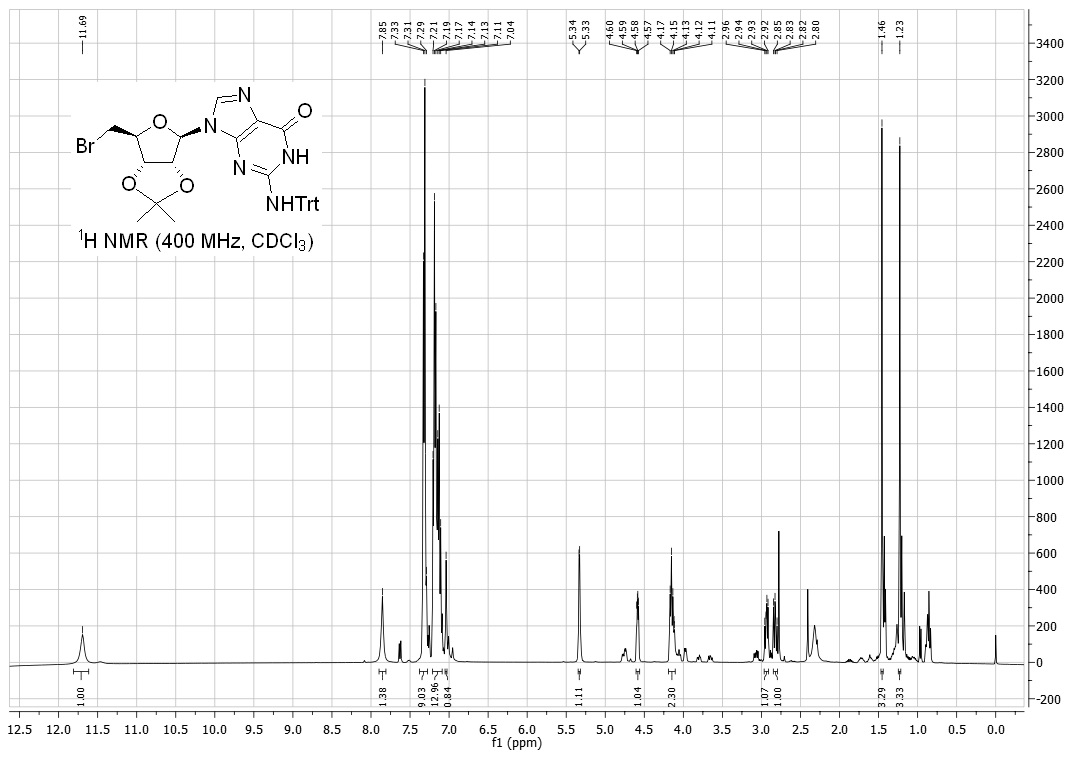


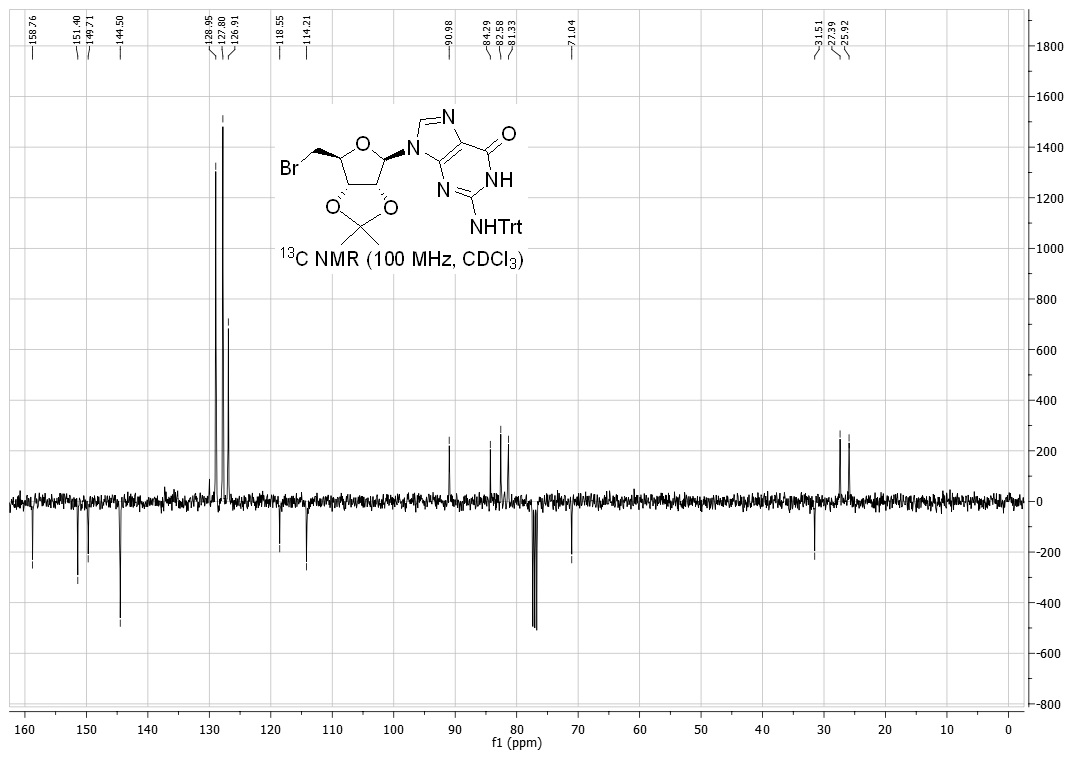


Compound **26**


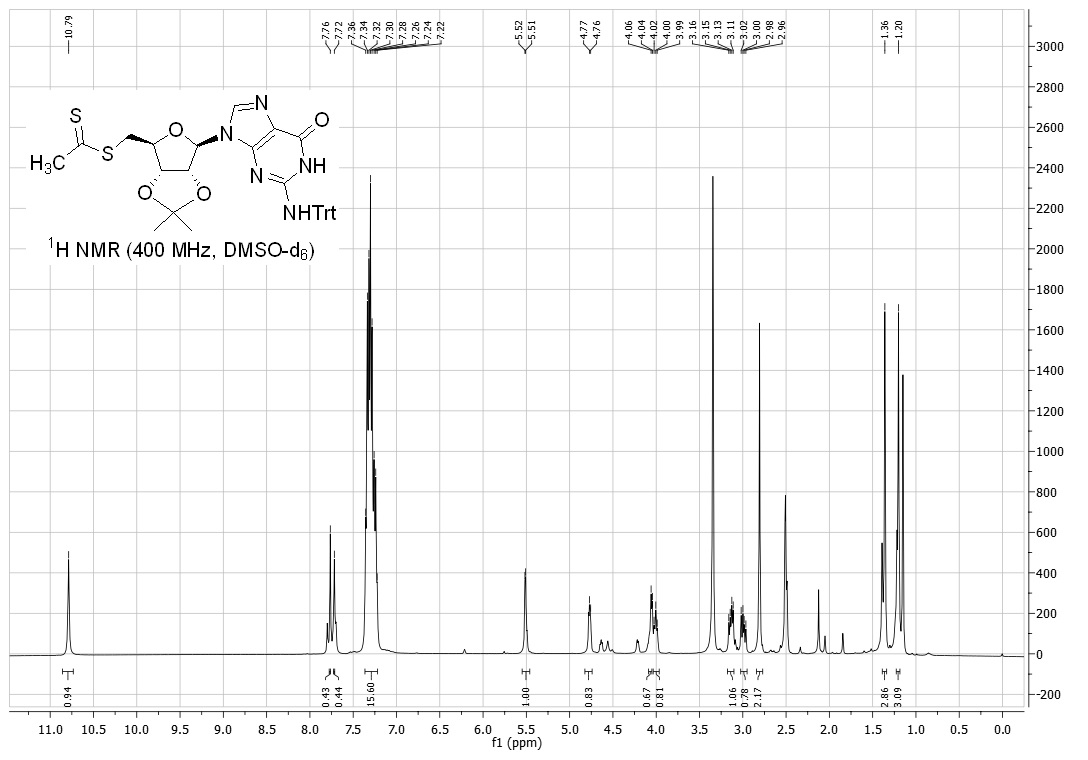


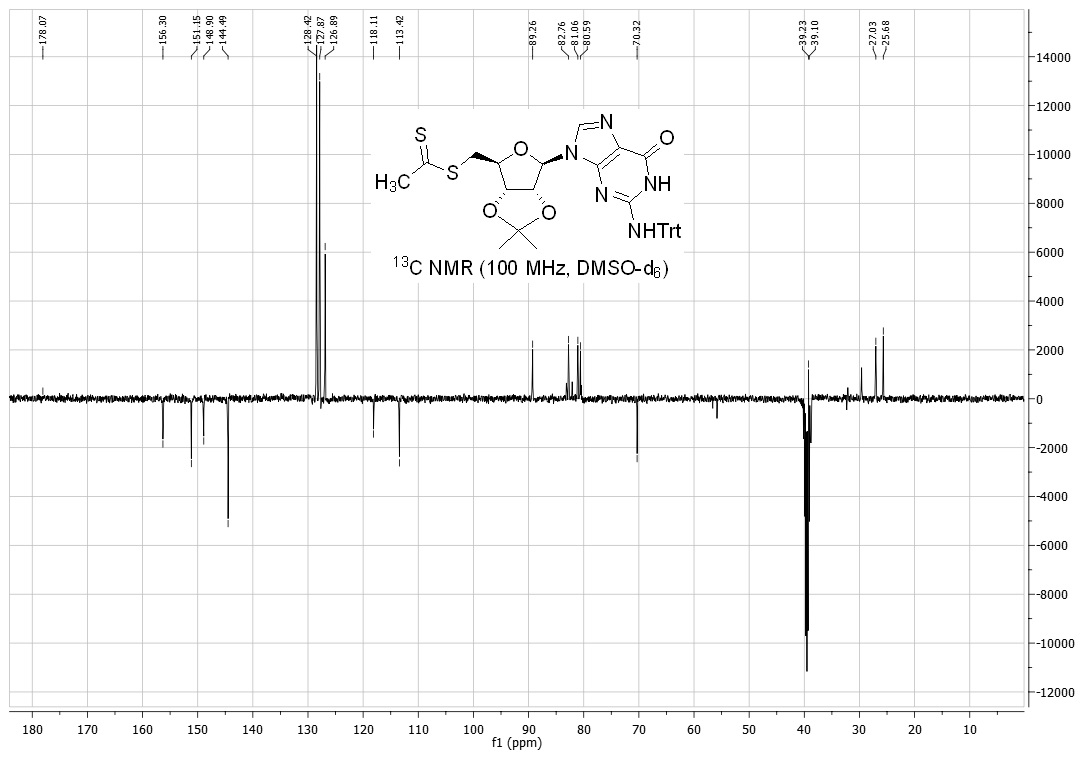


Compound **27**


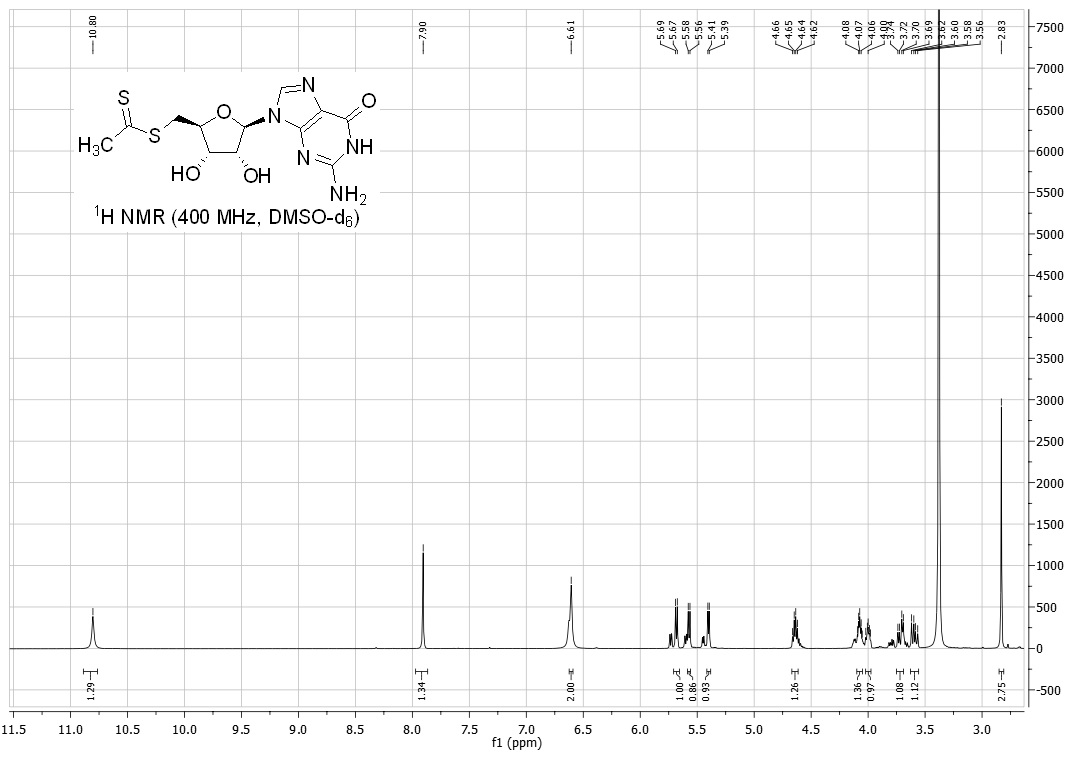


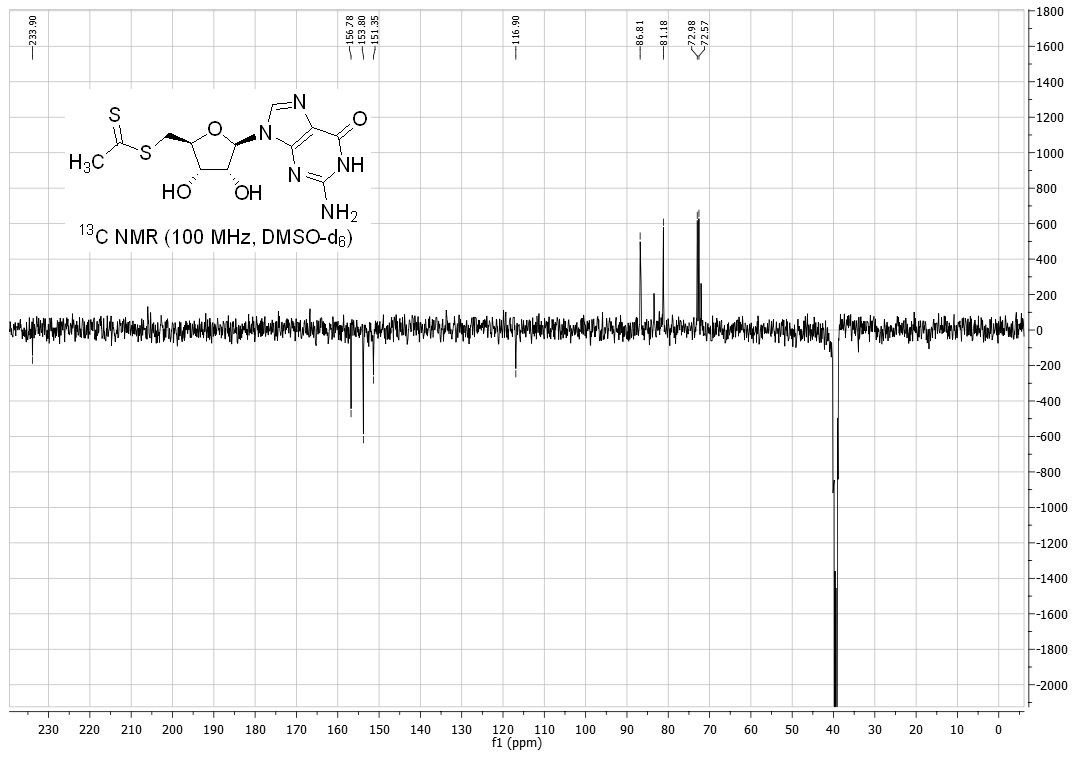


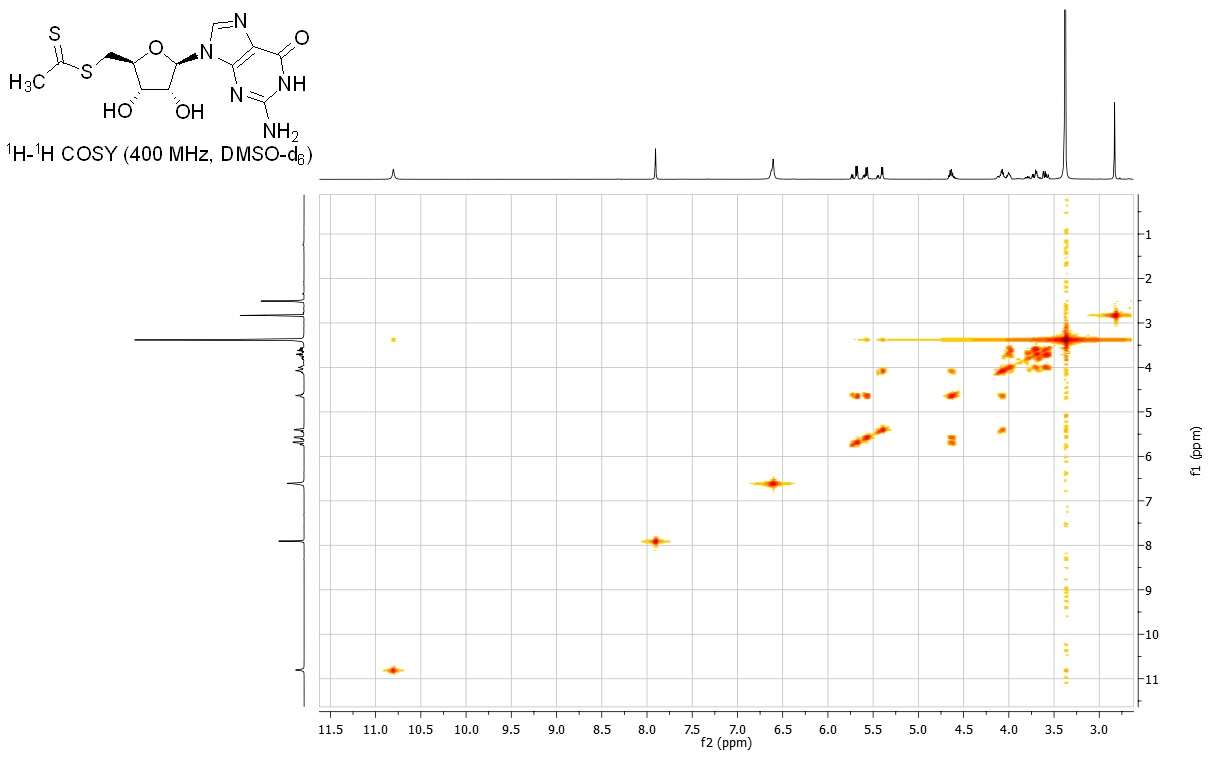


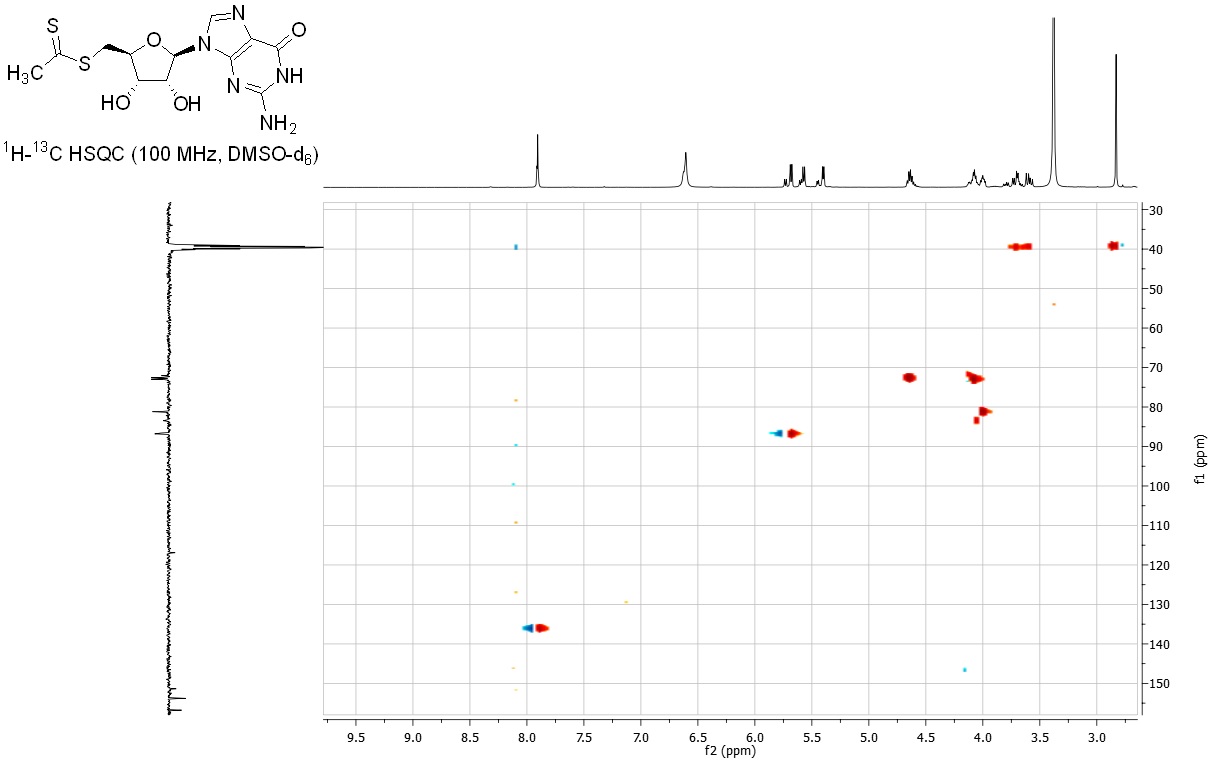


Compound **28**

**
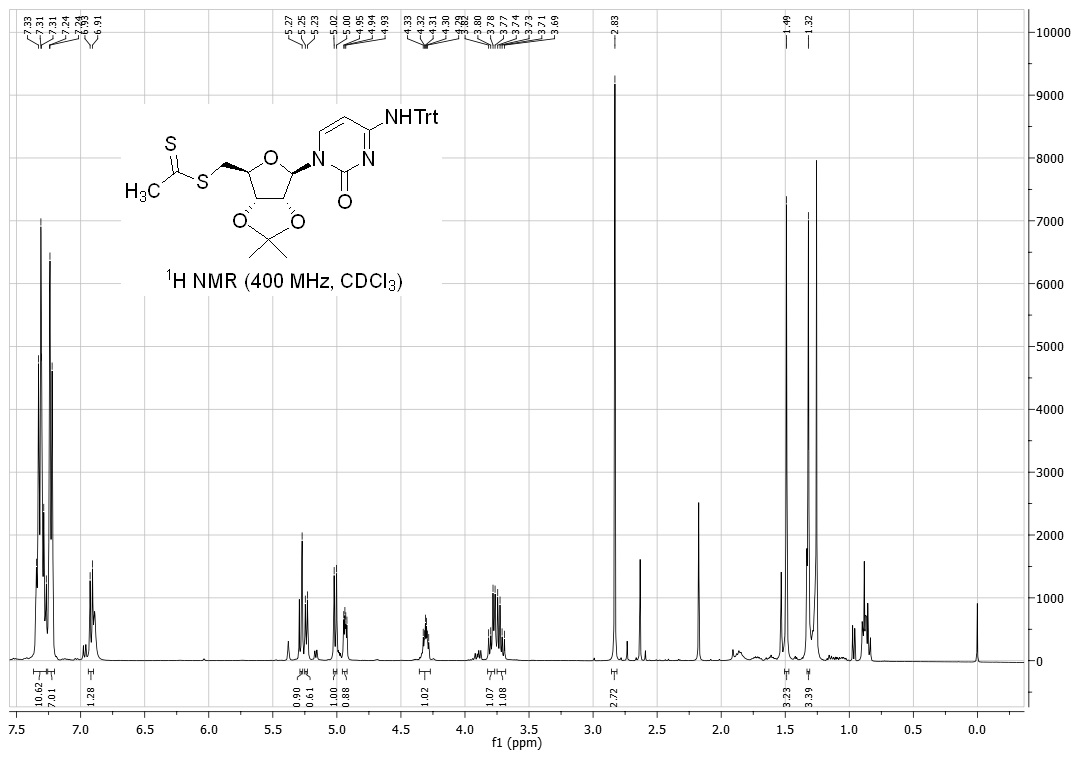
**


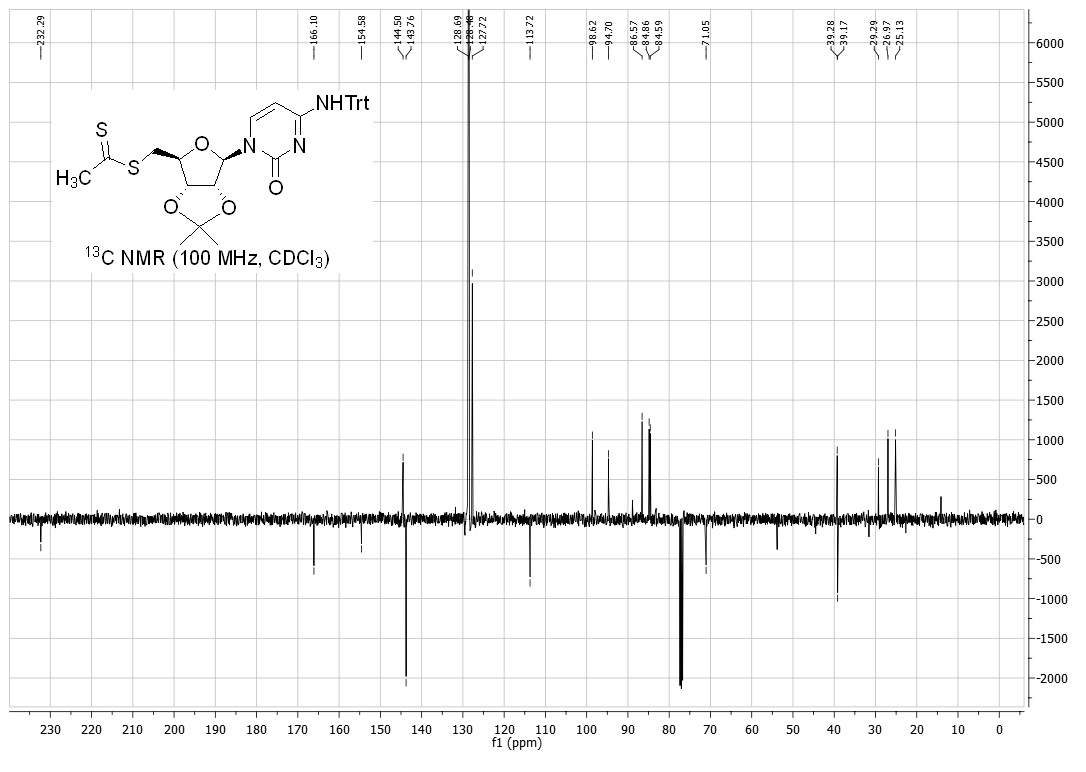


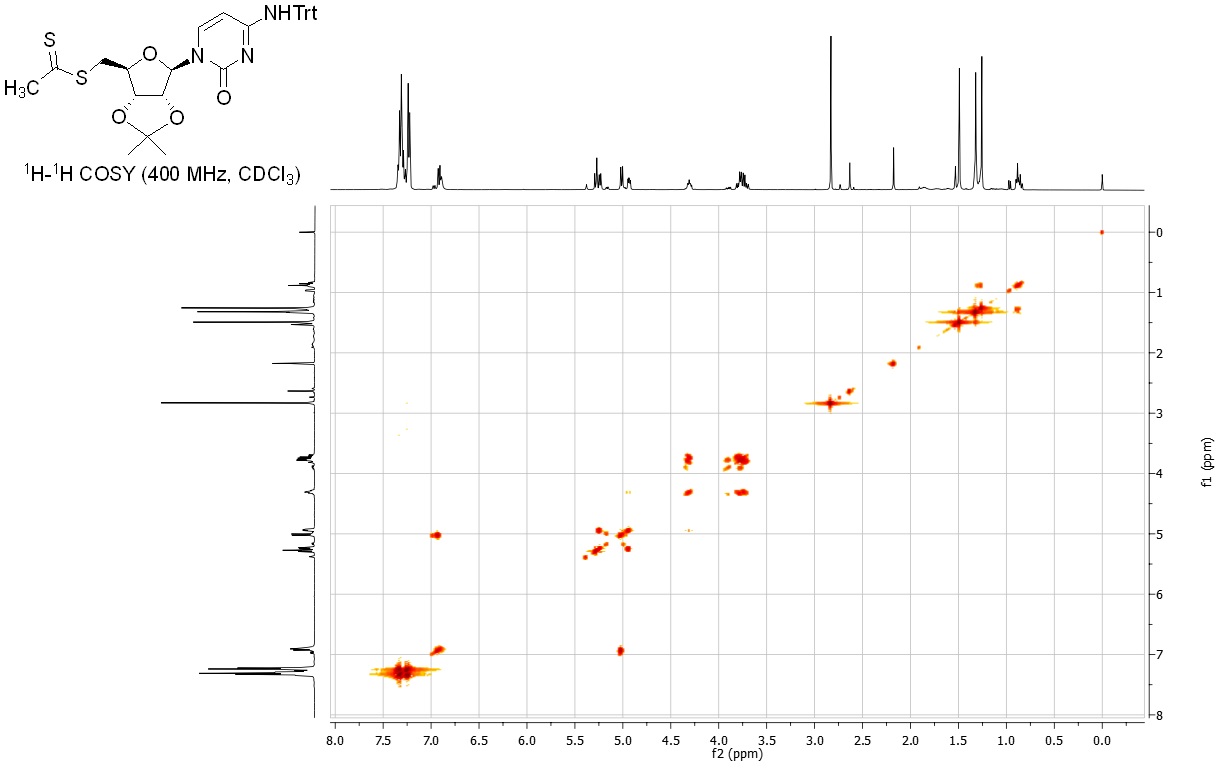


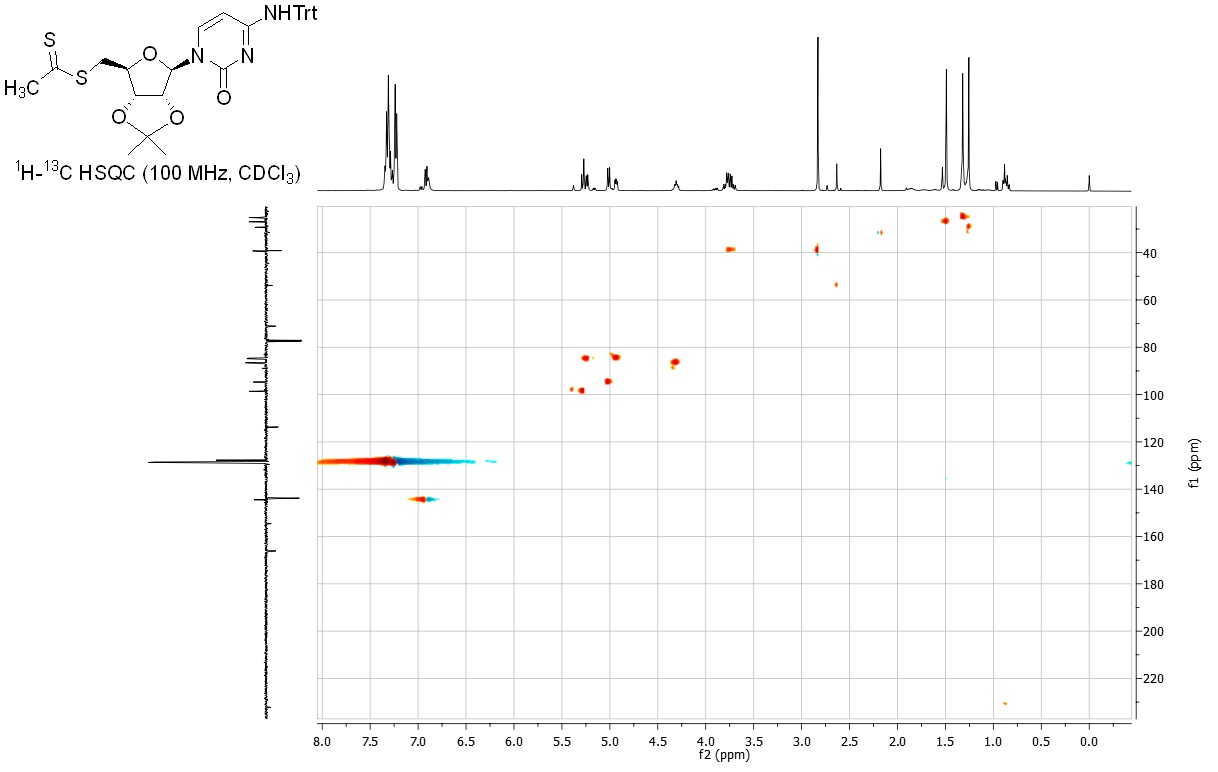


Compound **29**

**
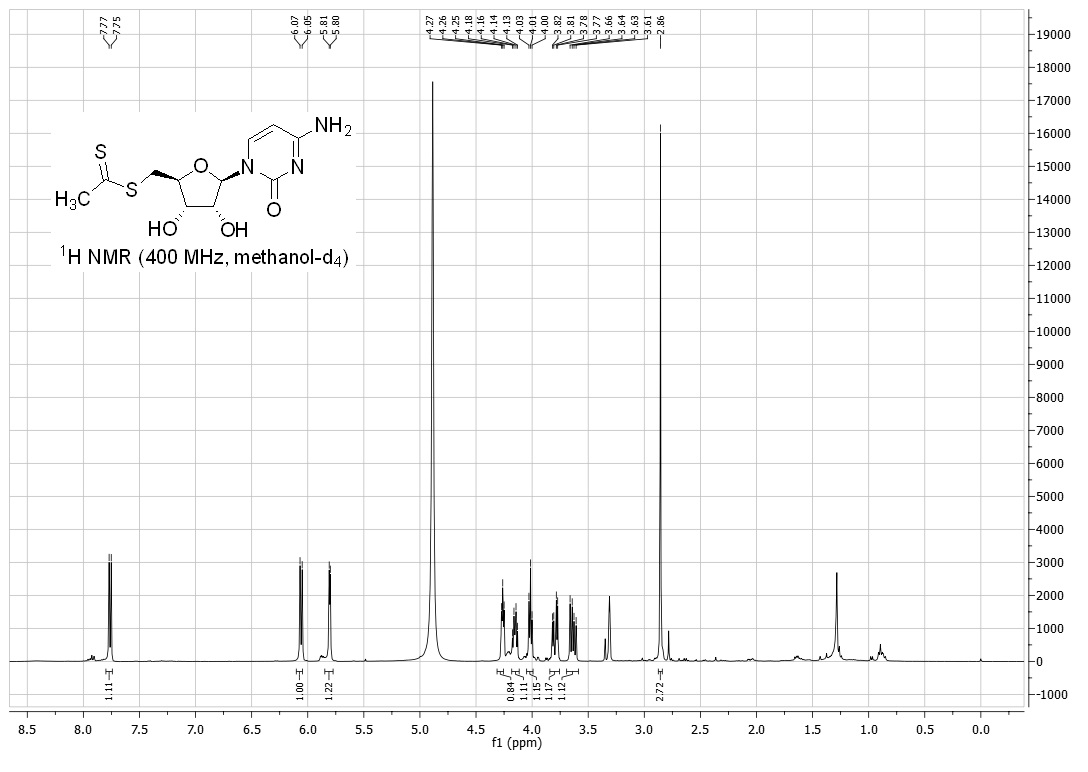
**

**
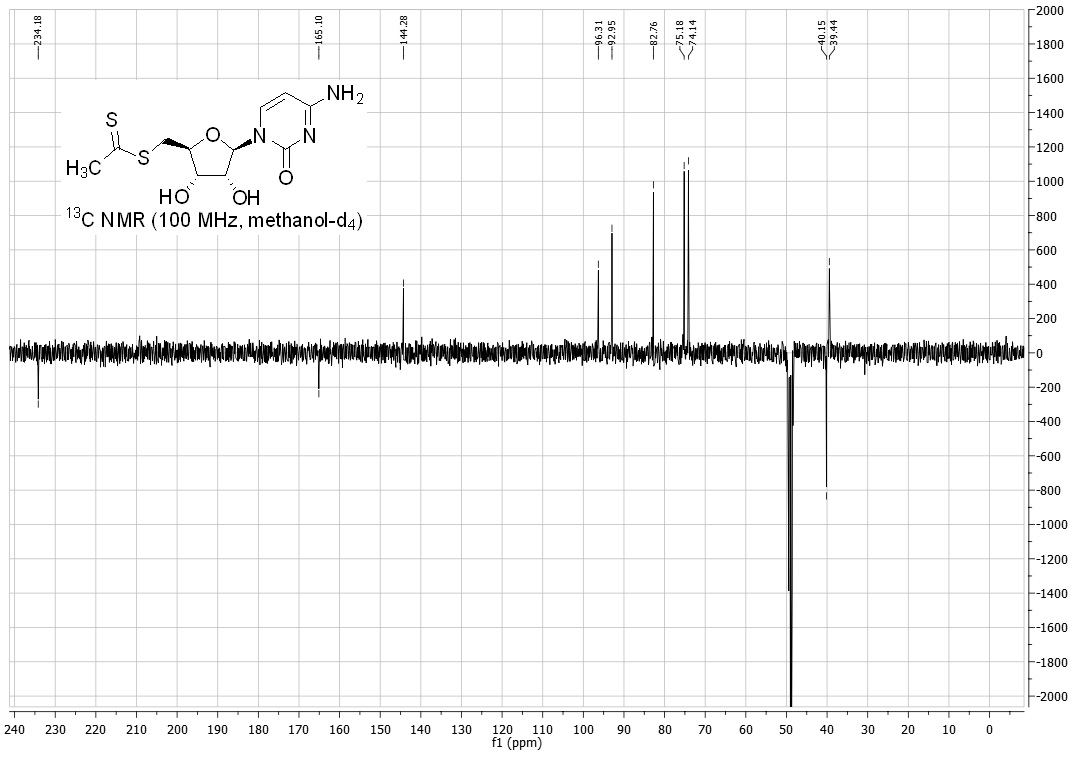
**

**
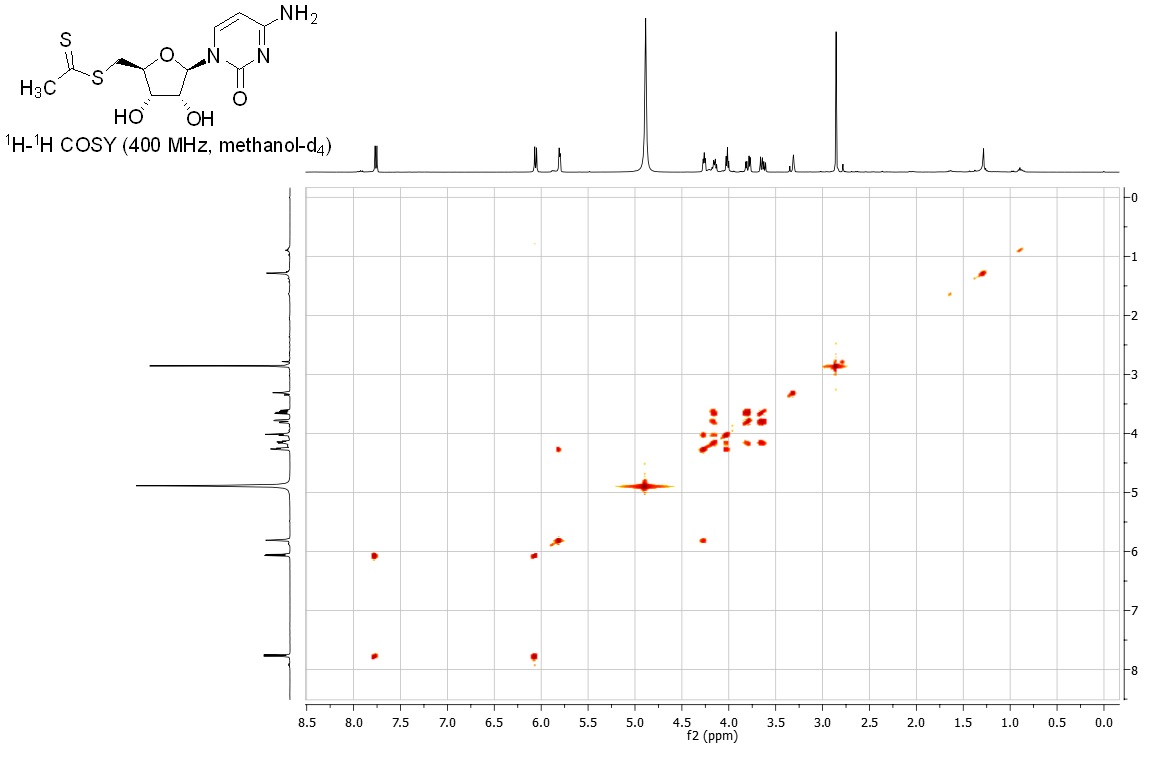
**

**
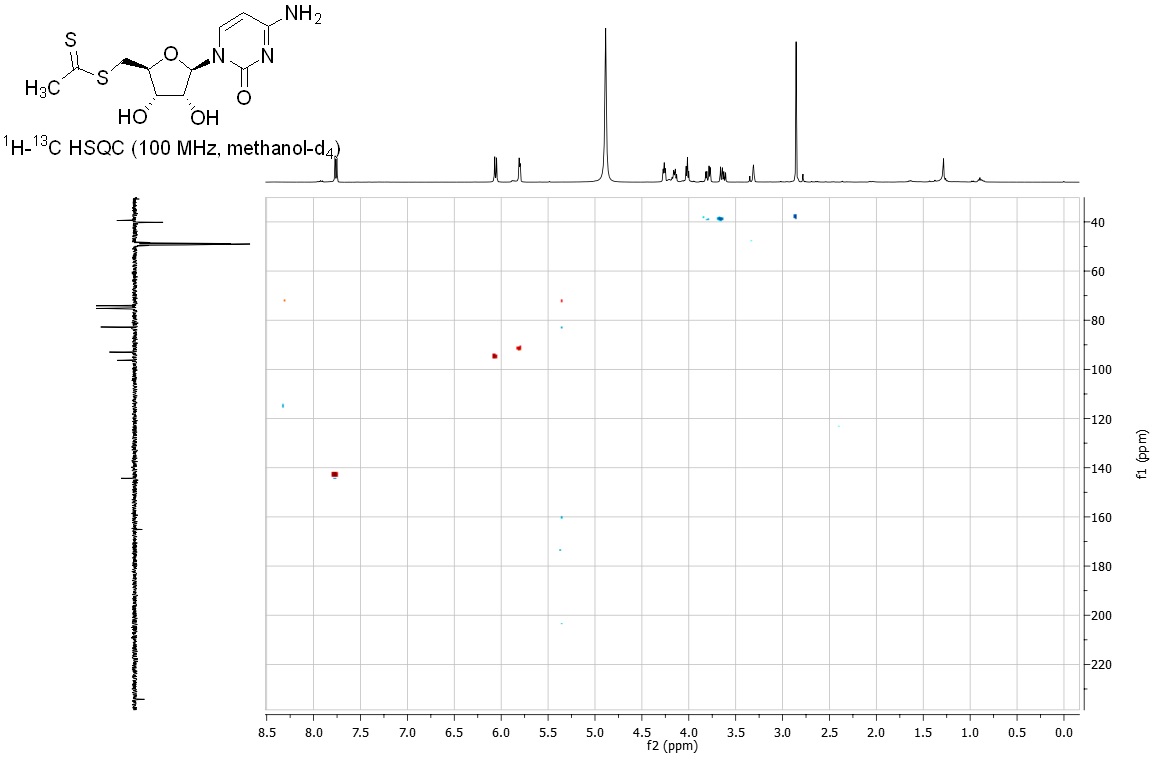
**

Compound **30**


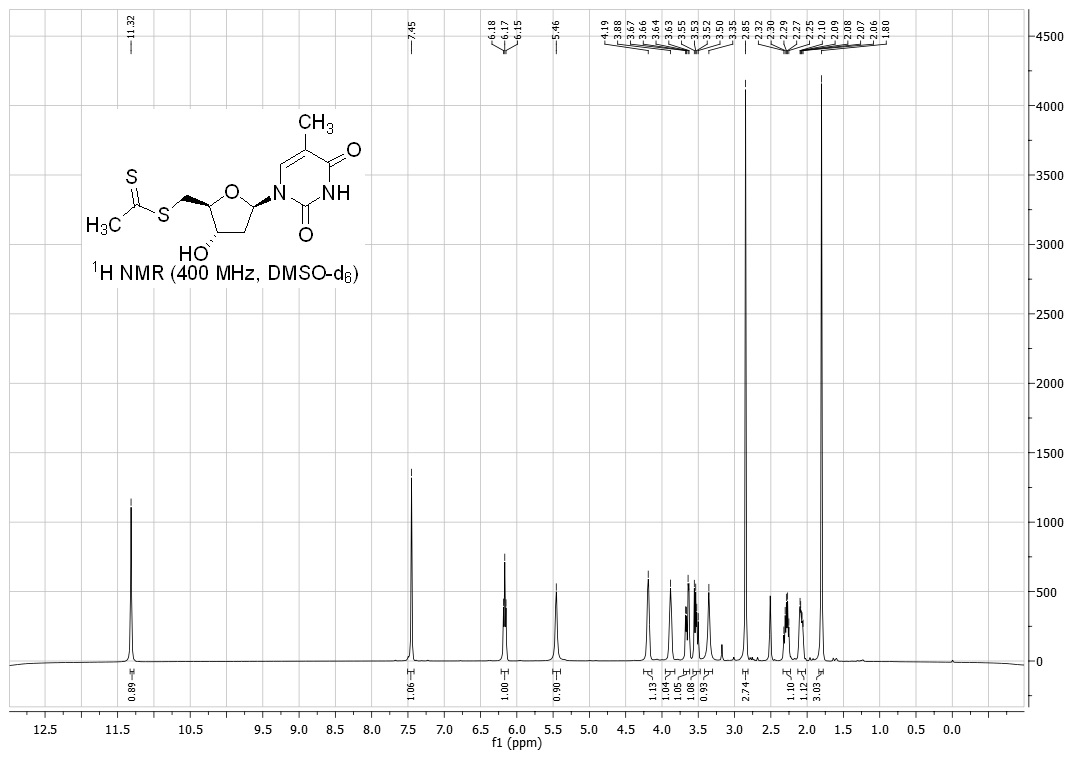


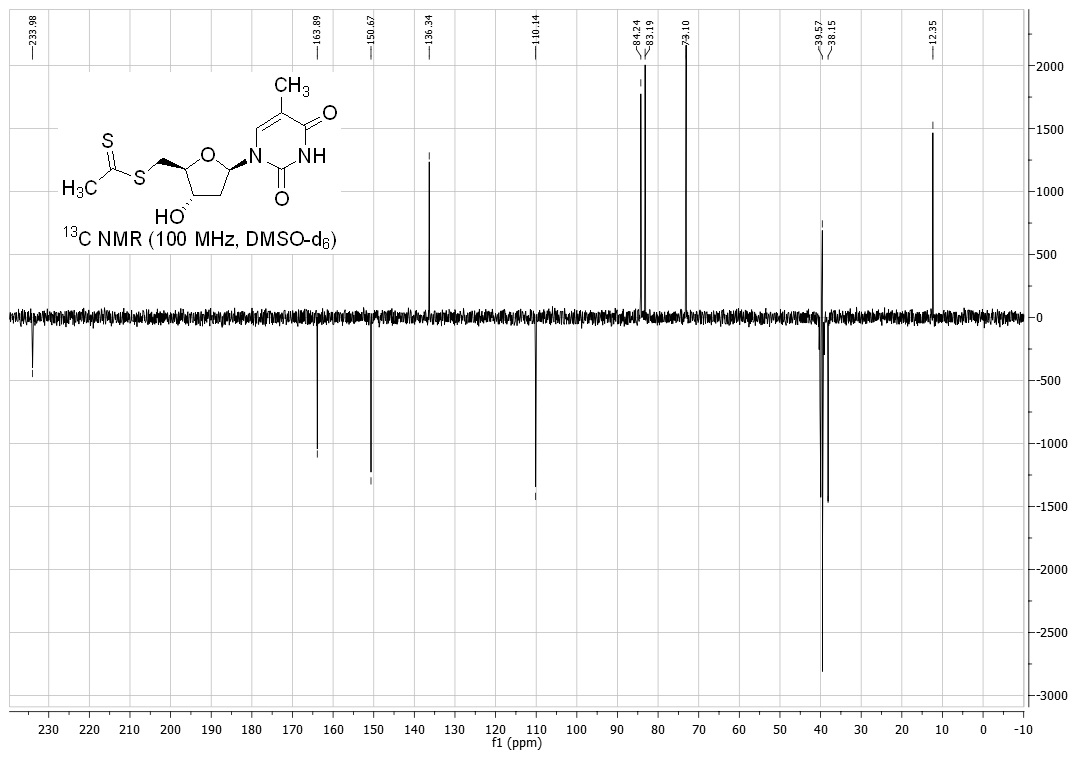


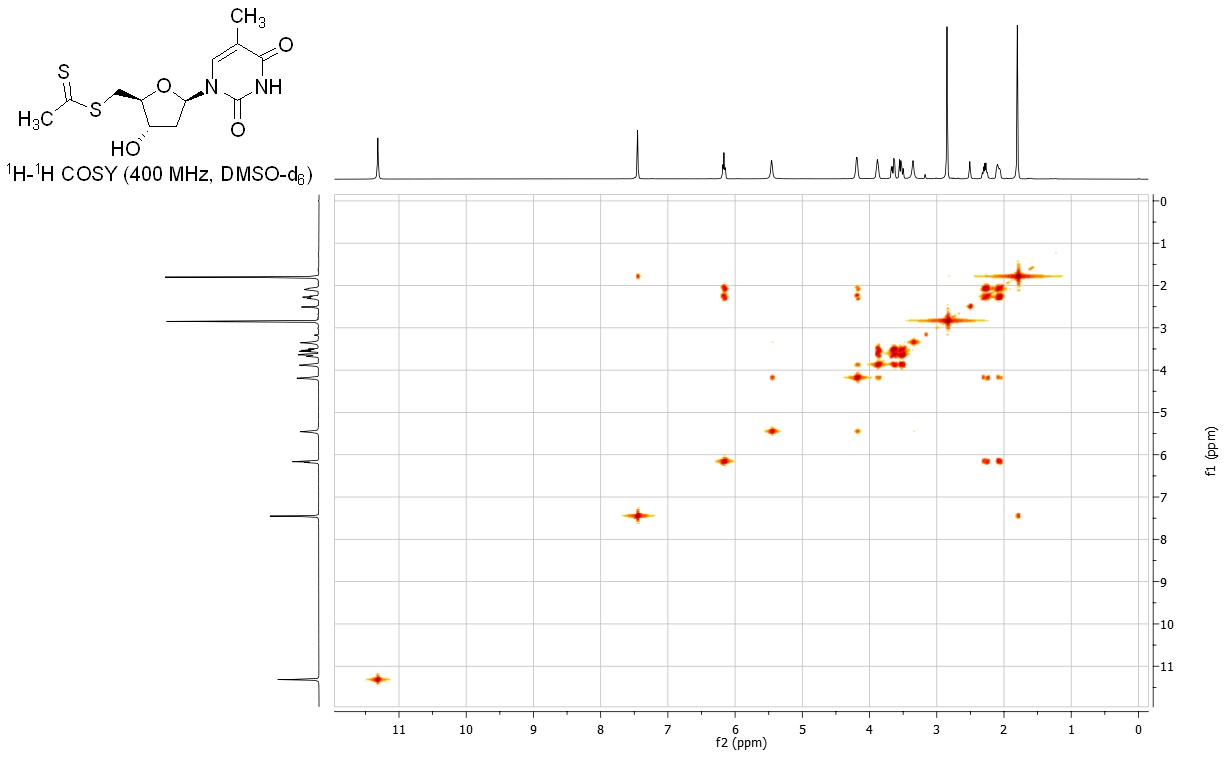


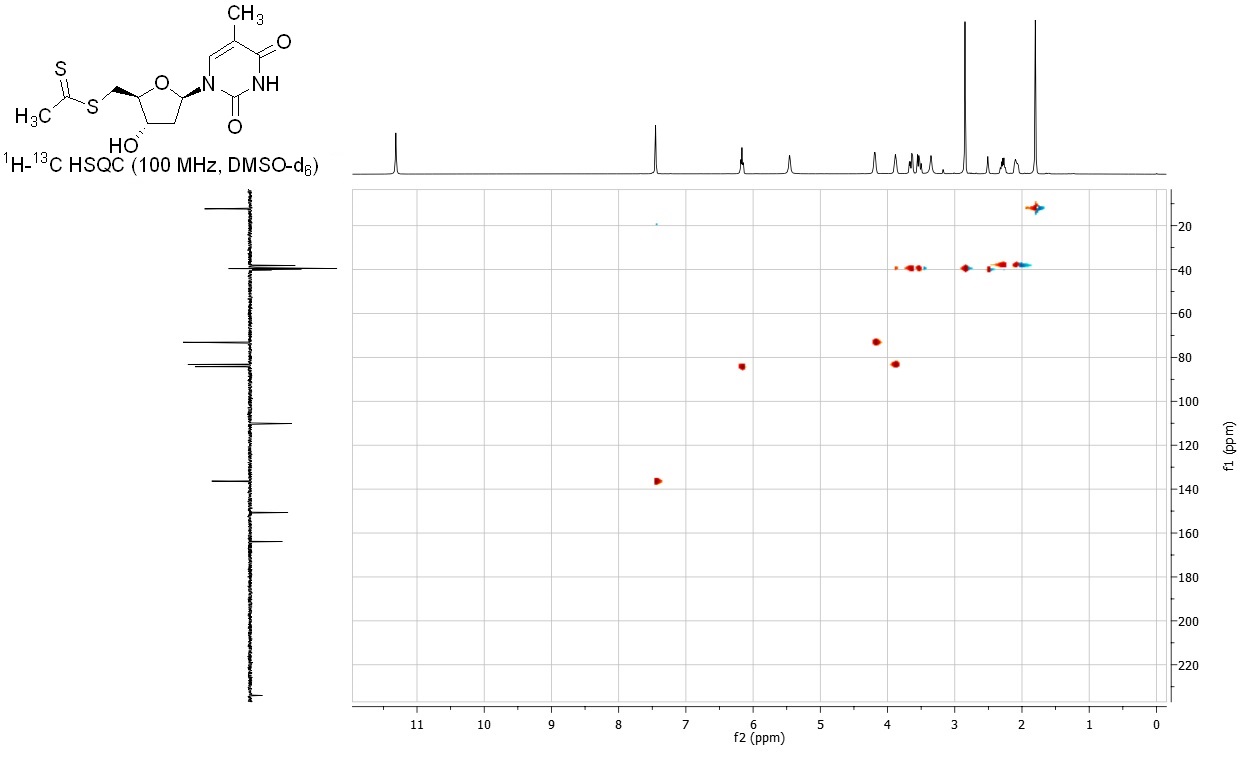


Compound **31**


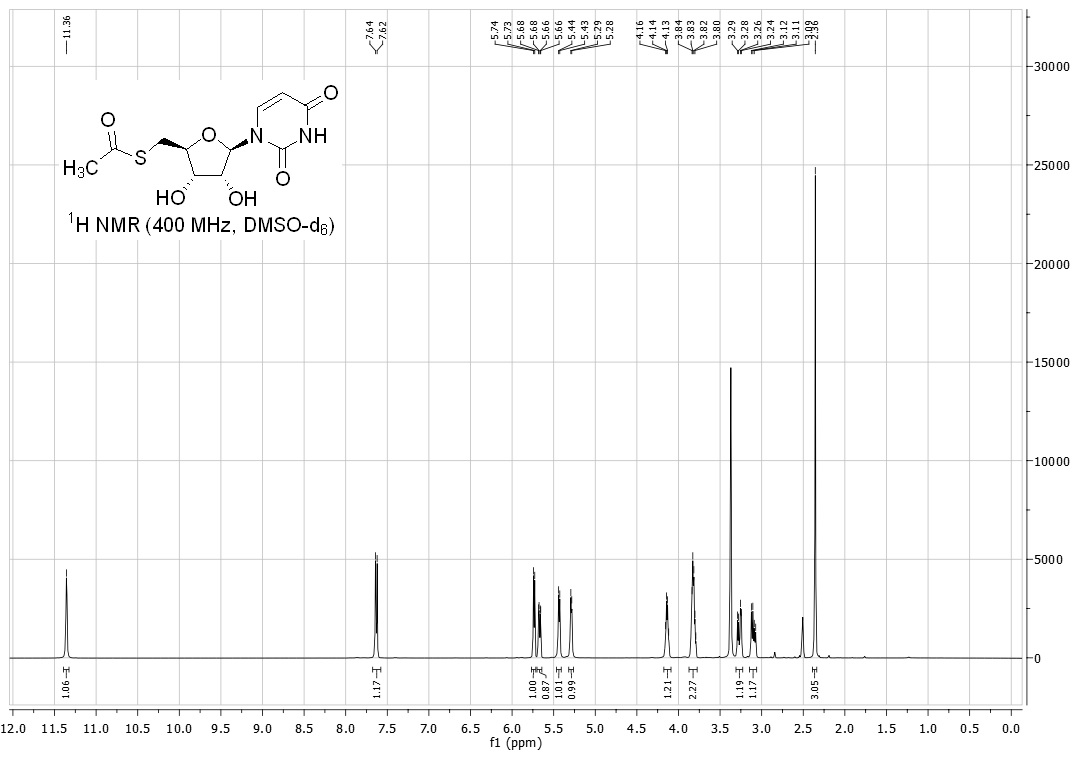


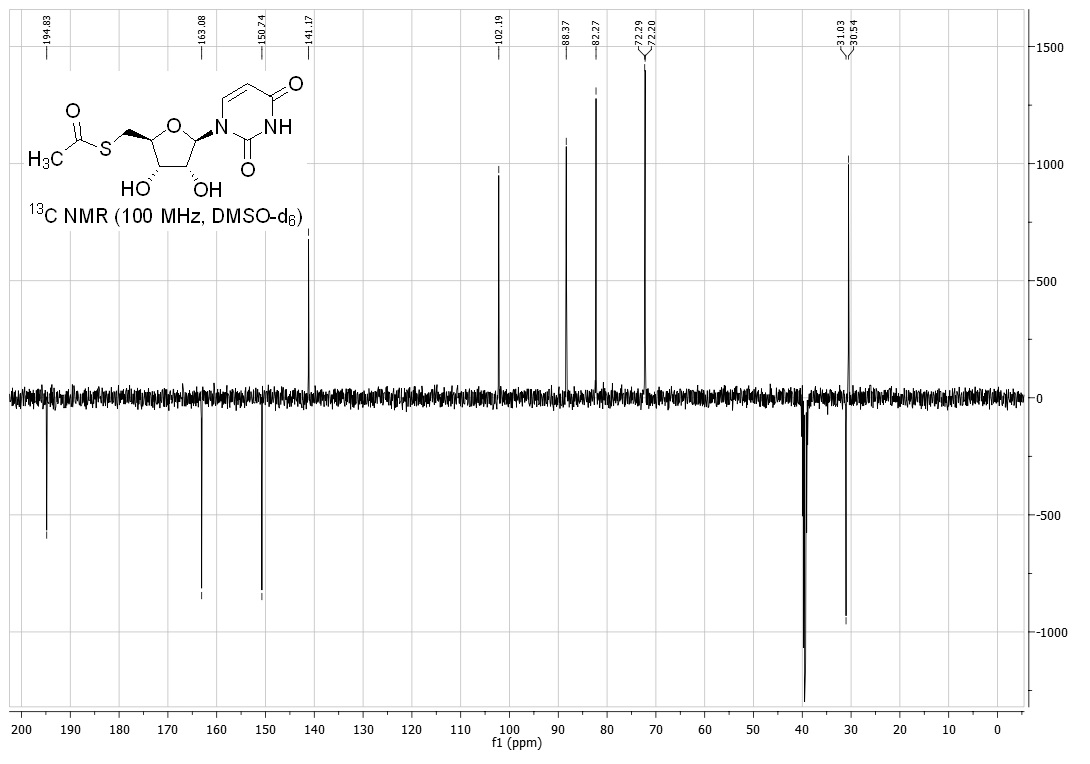


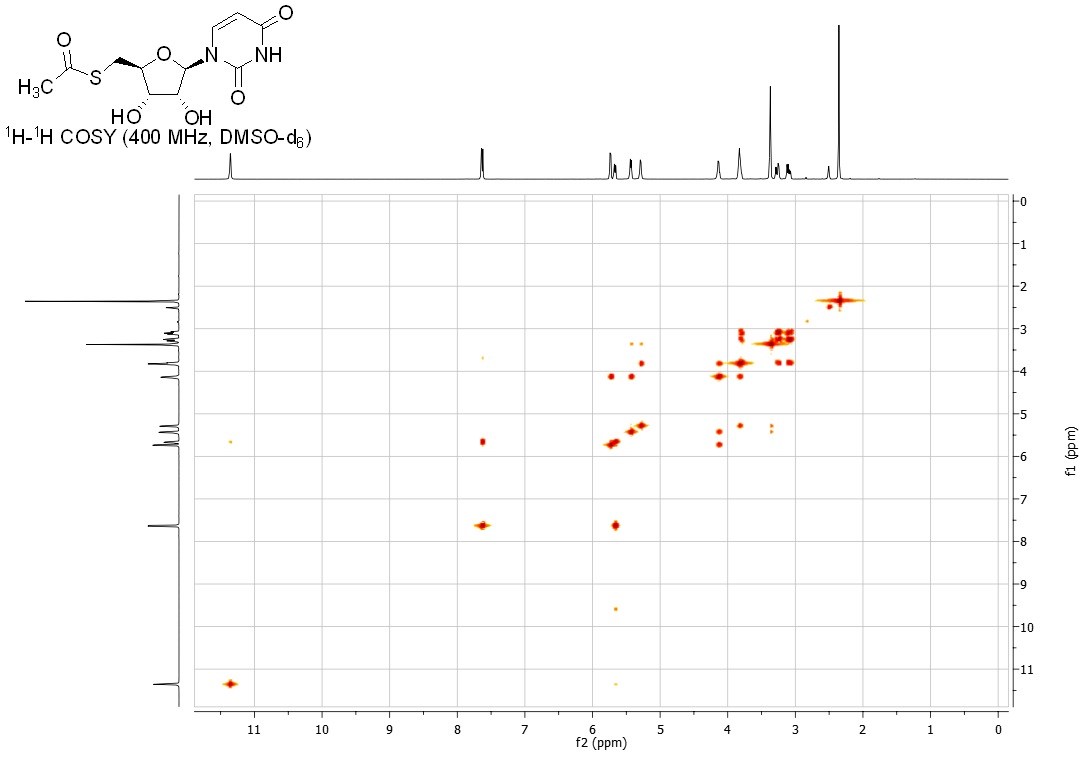


Compound **32**


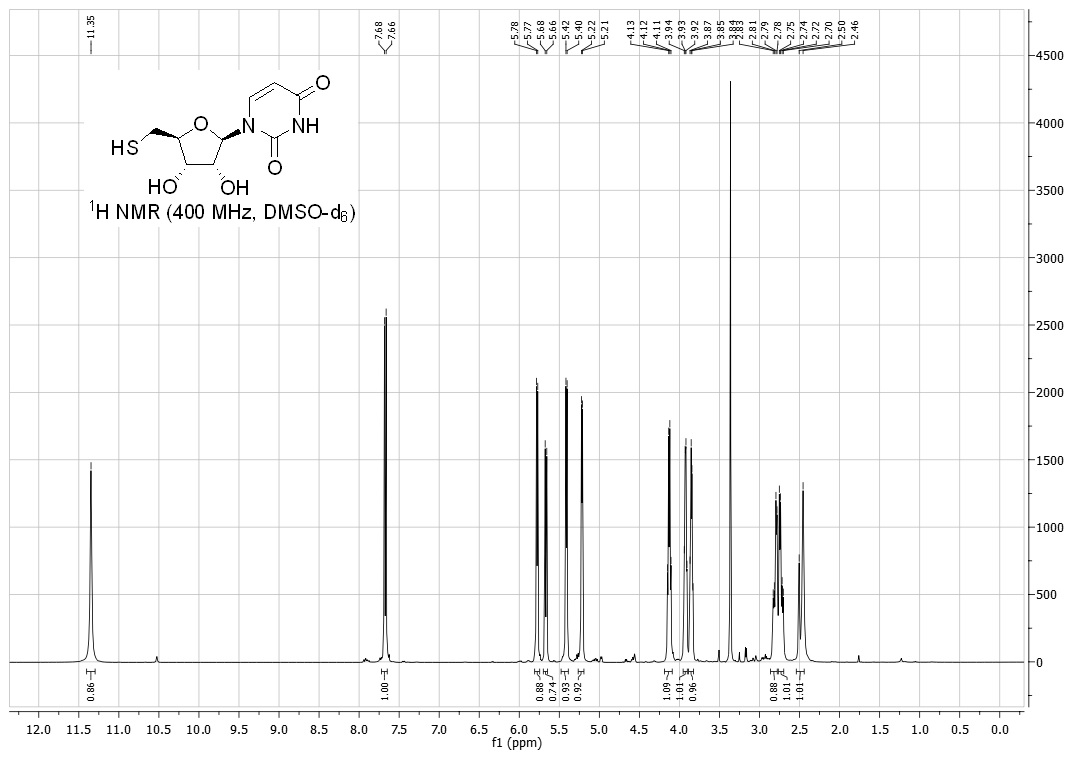


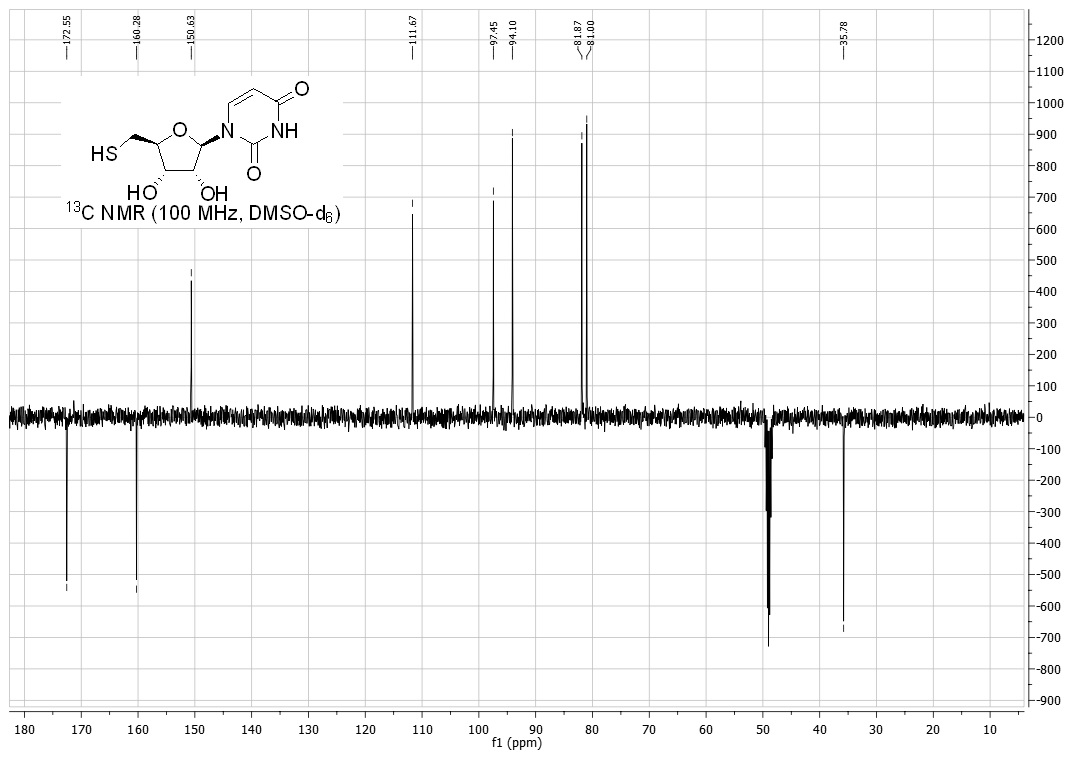


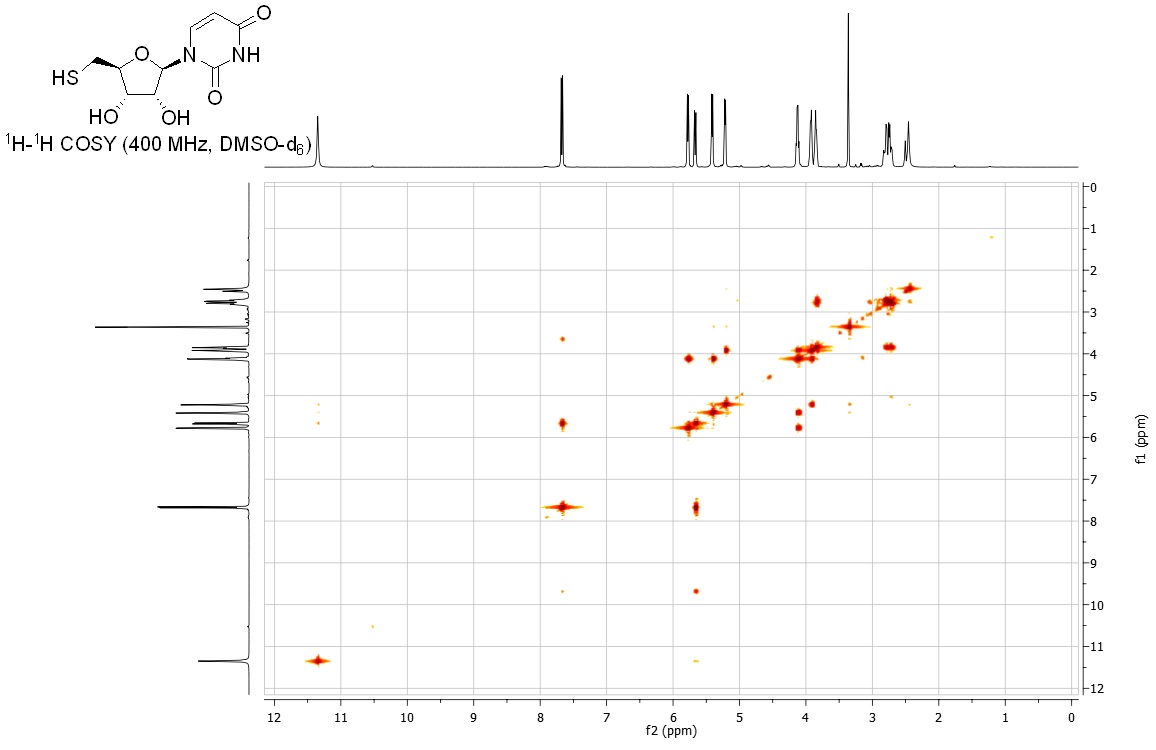


Compound **33**


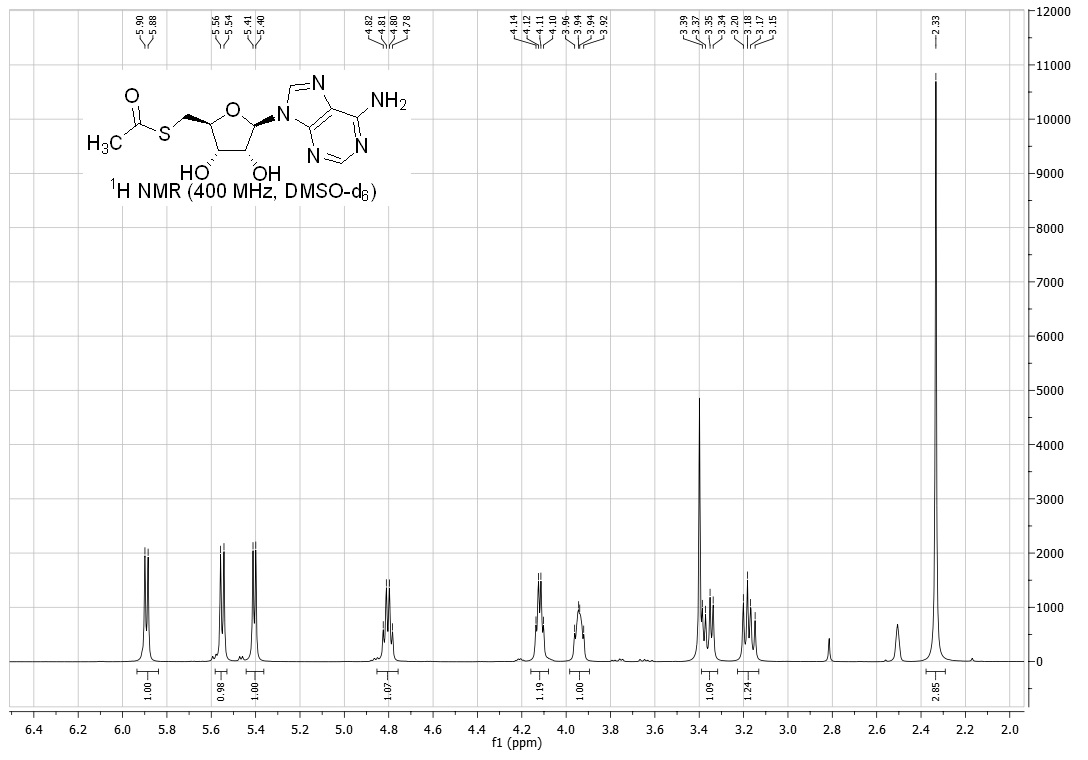


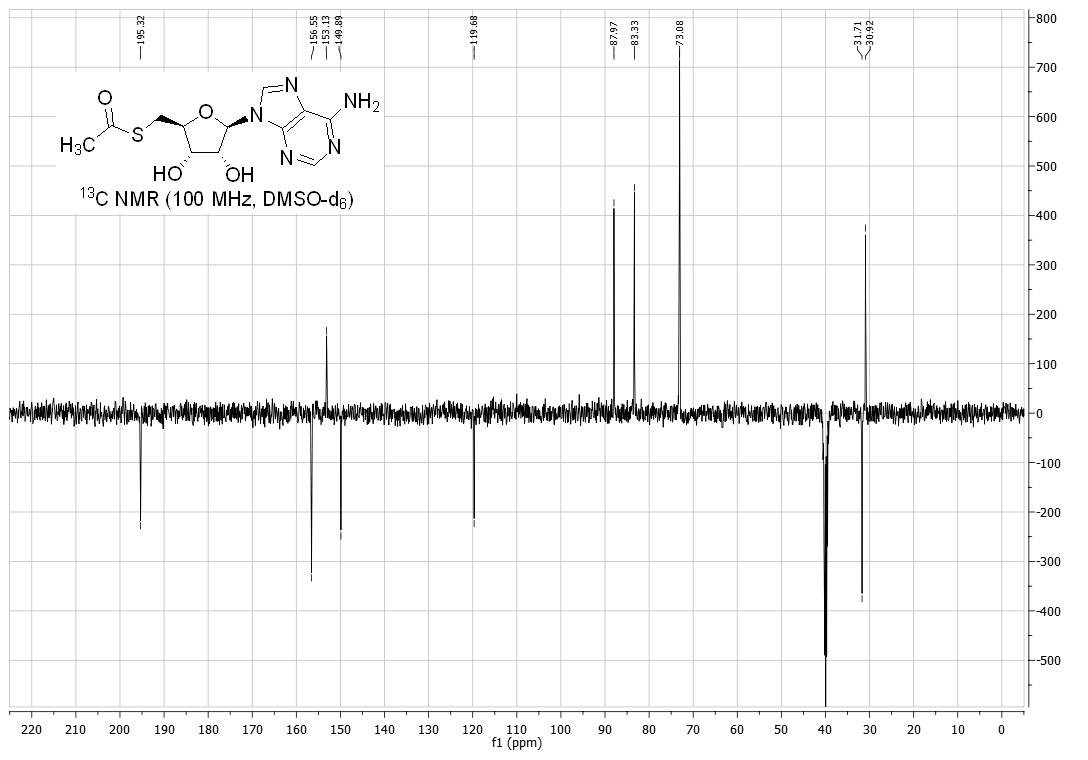


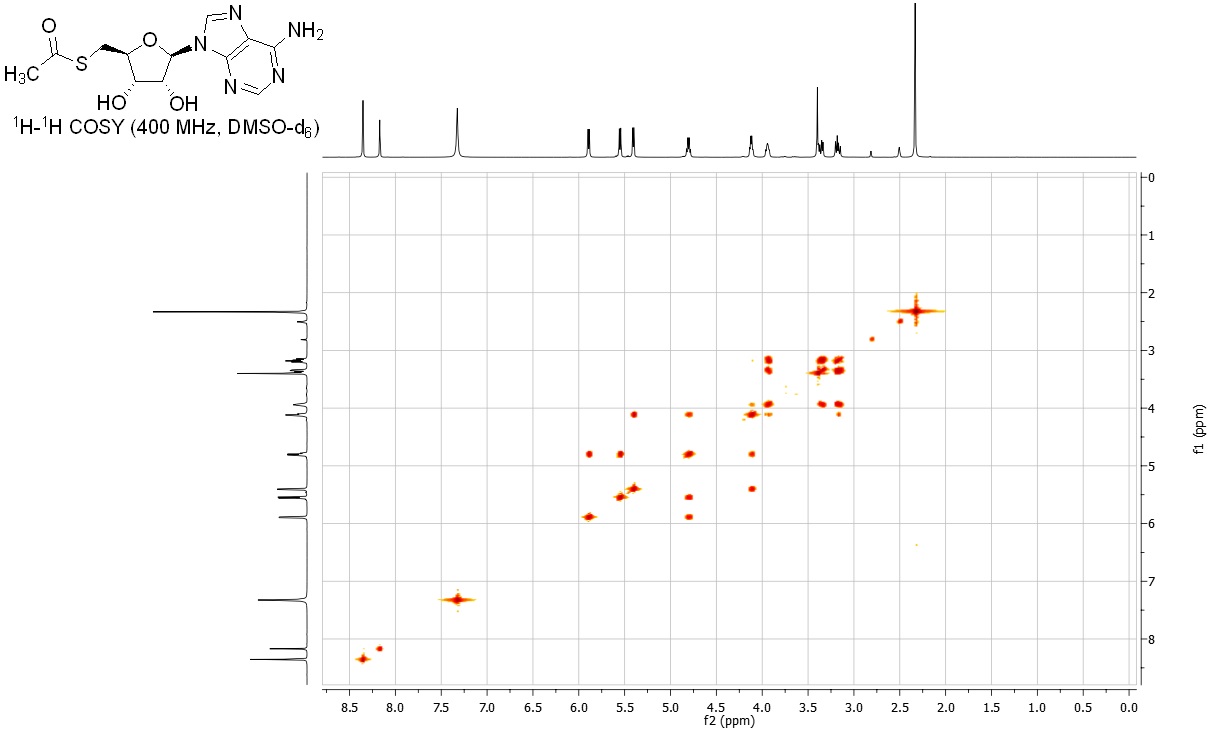


Compound **34**


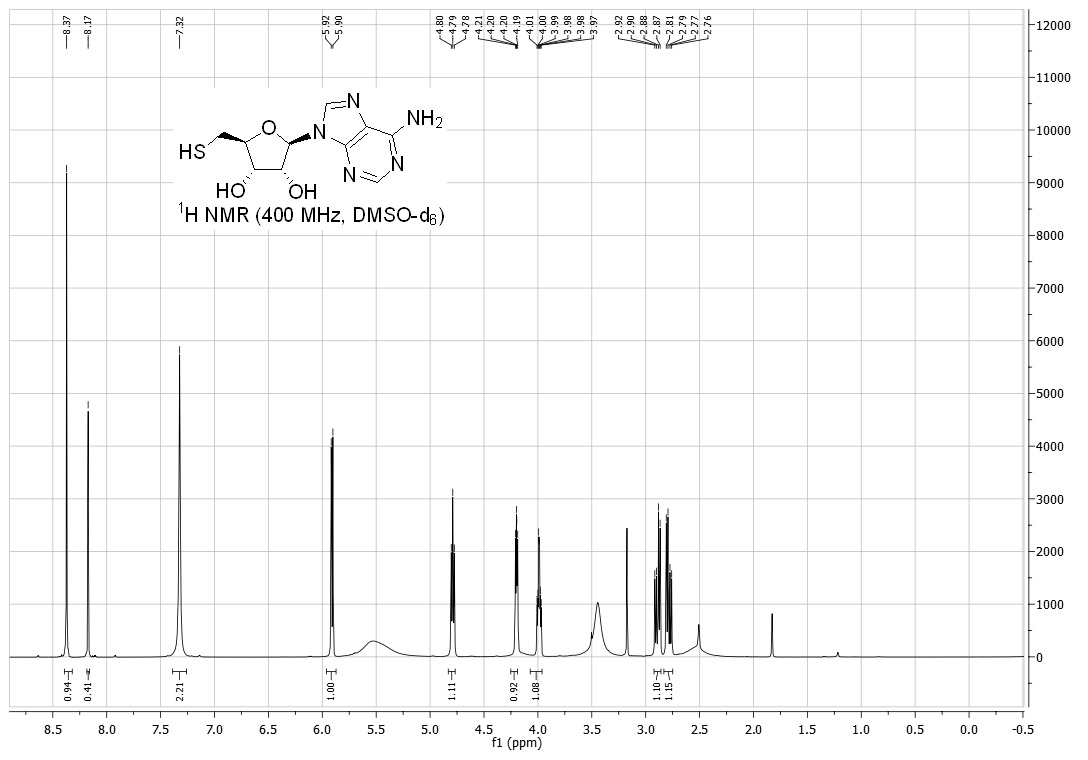


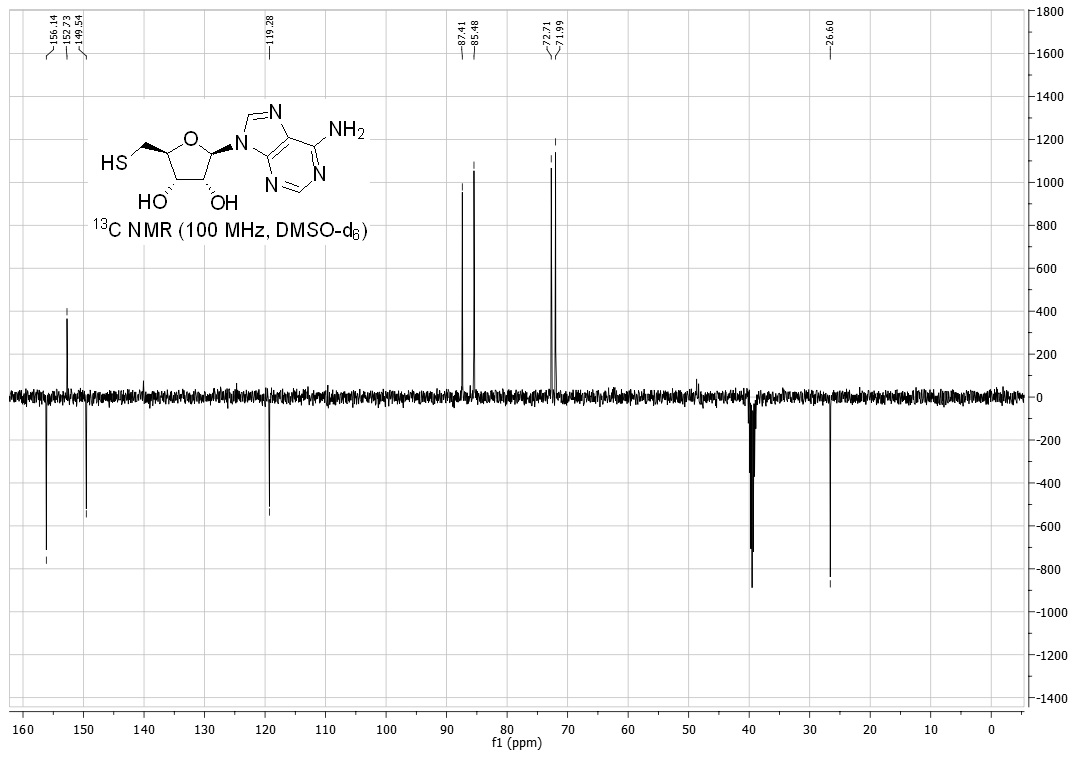


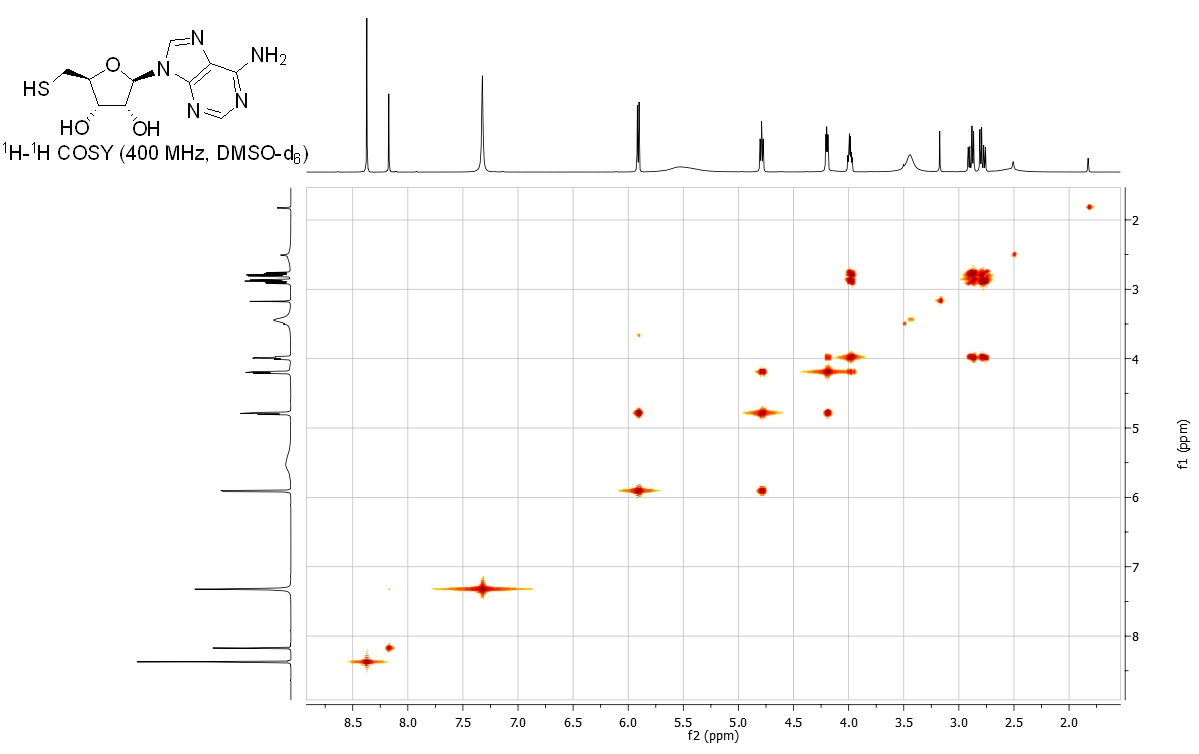

Supplement: Supplementary file 1 — Supplementary Material 1 [file 41598_2025_85351_MOESM1_ESM.docx]
